# Effect of biannual azithromycin on respiratory pathogens among symptomatic children: results from the randomised Macrolides Oraux pour Réduire les Décès avec un Oeil sur la Résistance (MORDOR) I trial

**DOI:** 10.1136/bmjgh-2024-016043

**Published:** 2025-02-10

**Authors:** Jie Liu, Stephanie A Brennhofer, Jixian Zhang, Suzanne Stroup, Suporn Pholwat, Ahmed M Arzika, Ramatou Maliki, Amza Abdou, Elodie Lebas, Kieran S O'Brien, Benjamin F Arnold, Jeremy D Keenan, Thomas M Lietman, James A Platts-Mills, Elizabeth T Rogawski McQuade, Eric R Houpt

**Affiliations:** 1 School of Public Health, Qingdao University, Qingdao, Shandong, China; 2 Division of Infectious Diseases & International Health, University of Virginia School of Medicine, Charlottesville, Virginia, USA; 3 Centre de Recherche et Interventions en Sante Publique, Niamey, Niger; 4 Carter Center, Niamey, Niger; 5 Programme Nationale de Santé Oculaire, Niamey, Niger; 6 Francis I Proctor Foundation, University of California San Francisco, San Francisco, California, USA; 7 Department of Ophthalmology, University of California San Francisco, San Francisco, California, USA; 8 Department of Epidemiology, Emory University, Atlanta, Georgia, USA

**Keywords:** Pneumonia, Pertussis

## Abstract

**Introduction:**

Biannual (ie, every 6 months) mass drug administration of azithromycin has reduced childhood mortality in Niger, but its effects on specific respiratory pathogens are not fully elucidated.

**Methods:**

Across 2 years of the Macrolides Oraux pour Réduire les Décès avec un Oeil sur la Résistance (MORDOR) study in Niger, we evaluated 1468 nasopharyngeal swabs from children who presented for care with respiratory symptoms. Swabs were tested by quantitative PCR using a customised TaqMan Array Card that included assays for 19 respiratory pathogens.

**Results:**

Nasopharyngeal detection of *Haemophilus influenzae*, *Moraxella catarrhalis* and *Streptococcus pneumoniae* was common in both azithromycin and placebo communities. The prevalence was reduced in children from azithromycin communities for just two pathogens: *Bordetella pertussis* and *H. influenzae* type b (Hib). These children had a 49% and 65% reduction in the prevalence of *B. pertussis* and Hib in nasopharyngeal swabs, respectively, compared with children from the control communities (prevalence ratios 0.51, 95% CI 0.35, 0.75; and 0.35, 95% CI 0.17, 0.71).

**Conclusions:**

Biannual administration of azithromycin to communities in Niger was associated with lower prevalence of *B. pertussis* and Hib compared with placebo. These reductions may explain some of the childhood mortality benefit of azithromycin.

**Trial registration number:**

NCT02048007.

**WHAT IS ALREADY KNOWN ON THIS TOPIC**

- Biannual azithromycin to children can reduce childhood mortality in high-mortality settings such as Niger; however, the effect of this antibiotic on specific pathogens is not clear.

**WHAT THIS STUDY ADDS**

- In symptomatic children from azithromycin versus placebo treated communities, there was a lower prevalence of *Bordetella pertussis* and *Haemophilus influenzae* type b.

**HOW THIS STUDY MIGHT AFFECT RESEARCH, PRACTICE OR POLICY**

- It is possible that azithromycin treatment serves as an additional layer of protection against vaccine-preventable bacterial infections such as *B. pertussis* and *H. influenzae* type b.

## Introduction

 The Macrolides Oraux pour Réduire les Décès avec un Oeil sur la Résistance (MORDOR) study was a cluster-randomised trial that demonstrated an 18.1% reduction in childhood mortality among Niger communities that received mass distribution of azithromycin two times a year.^1^ This study and other data have led to a conditional recommendation by the WHO for the mass drug administration of azithromycin in infants in high infant and child mortality settings that carry a heavy burden of malaria, pneumonia and diarrhoea.^2^ However, azithromycin’s effect on different infectious causes of death are unknown. It was anticipated that the broad-spectrum activity of azithromycin could reduce respiratory infections, diarrhoea and/or malaria.^3^ Verbal autopsy data from Niger suggested that azithromycin communities experienced a third fewer deaths from meningitis and dysentery and a fifth fewer deaths from malaria and pneumonia.^4^ Sample collection has been performed on a subset of children from azithromycin and placebo communities and has demonstrated reductions in malaria parasitaemia, *Shigella* intestinal carriage and *Campylobacter* positivity in azithromycin communities, suggesting broad effects of azithromycin on multiple important pathogens.^5^,^8^

By contrast, the aetiologies of respiratory illness have not been examined. In this work, we tested nasopharyngeal swabs from children in the MORDOR Niger study area presenting with respiratory symptoms for a broad panel of 19 pathogens that could cause upper or lower respiratory tract infection. A number of recent studies^9 10^ have measured the quantities of respiratory pathogens in the nasopharynx to better ascribe detections with disease. We leveraged these approaches to examine whether there were reductions in specific aetiologies of respiratory illness in MORDOR communities.

## Methods

This was a secondary data analysis of the Niger site of the MORDOR I Morbidity study, which has been previously described.^17 11^,^13^ Briefly, this study enrolled communities from the Loga and Boboye departments in the Dosso Region of Niger. There were five 6-month dosing periods where a house-to-house census was conducted. Communities were eligible for enrolment if they had a population between 200 and 2000 people at the time of census. Children within those communities were eligible to receive the assigned community-level intervention in their homes at the time of the census if they were between 1 and 59 months of age and weighed at least 3800 grams. Direct observation ensured compliance and actual azithromycin coverage was 90.4±10.4% in all sites. Although monitoring for the primary outcomes of the trial was performed in the community, nasopharyngeal swabbing was also performed at a random sample of 15 centres de santé integrés (CSIs) (ie, government-run health centres) once per year (ie, months 0, 12 and 24). During each of the annual monitoring periods, a consecutive series of 40 children aged 1–59 months with respiratory symptoms, defined as cough or difficulty breathing according to the WHO integrated management of childhood illness (ICMI) paradigm, were enrolled from each CSI and offered nasopharyngeal swabbing by clinic staff using flocked swabs (Copan, Murrieta, CA). Consecutive children were enrolled, regardless of whether they resided in a MORDOR study community. Swabs were stored in DNA/RNA Shield (Zymo, Irvine, CA) at 35°C while at the CSI for 2–4 weeks and subsequently at −20°C or colder. Data were collected at approximately 1-year intervals from 2015 to 2017, referred to in this report as: year 0 (near the first dose of biannual azithromycin), year 1 (near the third dose of biannual azithromycin) and year 2 (near the fifth dose of biannual azithromycin). Collections could have occurred before or after the most recent azithromycin distribution. MORDOR I was a community-based study, and nasopharyngeal swabs from this clinic-based secondary data analysis could not be linked to specific children who were known to receive or not receive azithromycin in the main study database. Therefore, it is not known for certain when or whether an individual child received azithromycin or placebo. Ethical approval was obtained by the UCSF Committee on Human Research and Comité d’Ethique du Niger, and informed consent procedures were performed as detailed in the original manuscript.^1^ This substudy tested research samples that did not contain patient identifiers and was therefore determined by the University of Virginia Institutional Review Board to not meet criteria of Human Subjects Research. Patients or the public were not involved in the design, conduct, reporting or dissemination plans of our research. The original study can be found at ClinicalTrials.gov: NCT02048007.

### Patient and public involvement

This substudy’s research design, methodology, reporting and dissemination plans did not involve input from participants or the general public.

Total nucleic acid was extracted from swab samples preserved in DNA/RNA shield (Zymo) with QIAamp MinElute Virus Spin kit on QIAcube (Qiagen) following the manufacturer’s instruction. External controls MS2 and PhHV were spiked into each sample to monitor inhibition. One extraction blank was incorporated for up to 36 samples to rule out lab contamination. Each 100 µL of mixture consisting of 46 µL of the nucleic acid extract, 50 µL of AgPath One Step reverse-transcription-PCR (RT-PCR) buffer and 4 µL of enzyme mix was loaded onto each port of a customised TaqMan Array Card. The quantitative PCR assays used are shown in online supplemental table 1. The real-time RT-PCR was performed with the cycling condition of 20 min at 45°C, 10 min at 95°C, 40 cycles of 15 s at 95°C and 1 min at 60°C, with ViiA 7 or QuantStudio 7 Real-Time PCR systems. Assay linearity was calculated for each target by testing a serial dilution of the positive control with known concentration, based on which the conversion between cycle threshold (Ct) values and copy numbers was performed. Ct values for all pathogens on all specimens is included as a online supplemental file.

To capture pathogen burden, we calculated the proportion of positive swabs based on Ct value <35. The average quantity of 19 respiratory pathogens, which were selected a priori, were: adenovirus, bocavirus, cytomegalovirus (CMV), human metapneumovirus, influenza A virus, influenza B virus, human parainfluenza 1–4, rhinovirus, respiratory syncytial virus (RSV), *Bordetella pertussis*, *Haemophilus influenzae*, *Haemophilus influenzae* type B (Hib), *Moraxella catarrhalis*, *Mycoplasma pneumoniae*, *Neisseria meningitidis*, *Staphylococcus aureus*, *Streptococcus pneumoniae*, *Streptococcus pyogenes* and *Pneumocystis jirovecii* at years 0, 1 and 2 that was calculated as (35-pathogen Ct value)/3.322. For detections that converted to 0 (ie, Ct values ≥35 or no amplification), we further converted to log10(0.5) as 0 does not fit on the log scale.

Excluding non-study communities, we calculated relative differences in prevalence between the treatment and control groups at years 0, 1 and 2, by estimating prevalence ratios via the Poisson approximation of log-binomial regression with generalised estimating equations (GEE) to account for community clustering, adjusted for clinic visit and month of swab collection. Further, we calculated absolute reductions of pathogen burden between the treatment and control groups, by estimating the prevalence difference, adjusted for clinic visit and month of swab collection, using linear regression with GEE. Additionally, excluding non-detections (Ct ≥35 or no amplification), we estimated pathogen quantity differences between the treatment and control groups at years 0, 1, and 2 using linear regression with GEE, adjusted for clinic visit and month of swab collection. The interactions between treatment group and time point (years 0, 1, and 2) were not significant, and/or numbers were too small to assess heterogeneity by time point, so we collapsed across time points. Further adjustment for sex and year of age did not change the estimates, so they were not included in the final model. In an exploratory analysis, to estimate the burden of illness attributed to each pathogen, we estimated pathogen attributable fractions as $AF=prevalence\times \left(1-\frac{1}{OR}\right)$, where the OR was the pathogen-specific ORs published in the Pneumonia Etiology Research for Child Health (PERCH) study^10^ and the proportion was the proportion of positive samples from the MORDOR substudy sample.

## Results

**Figure 1:**
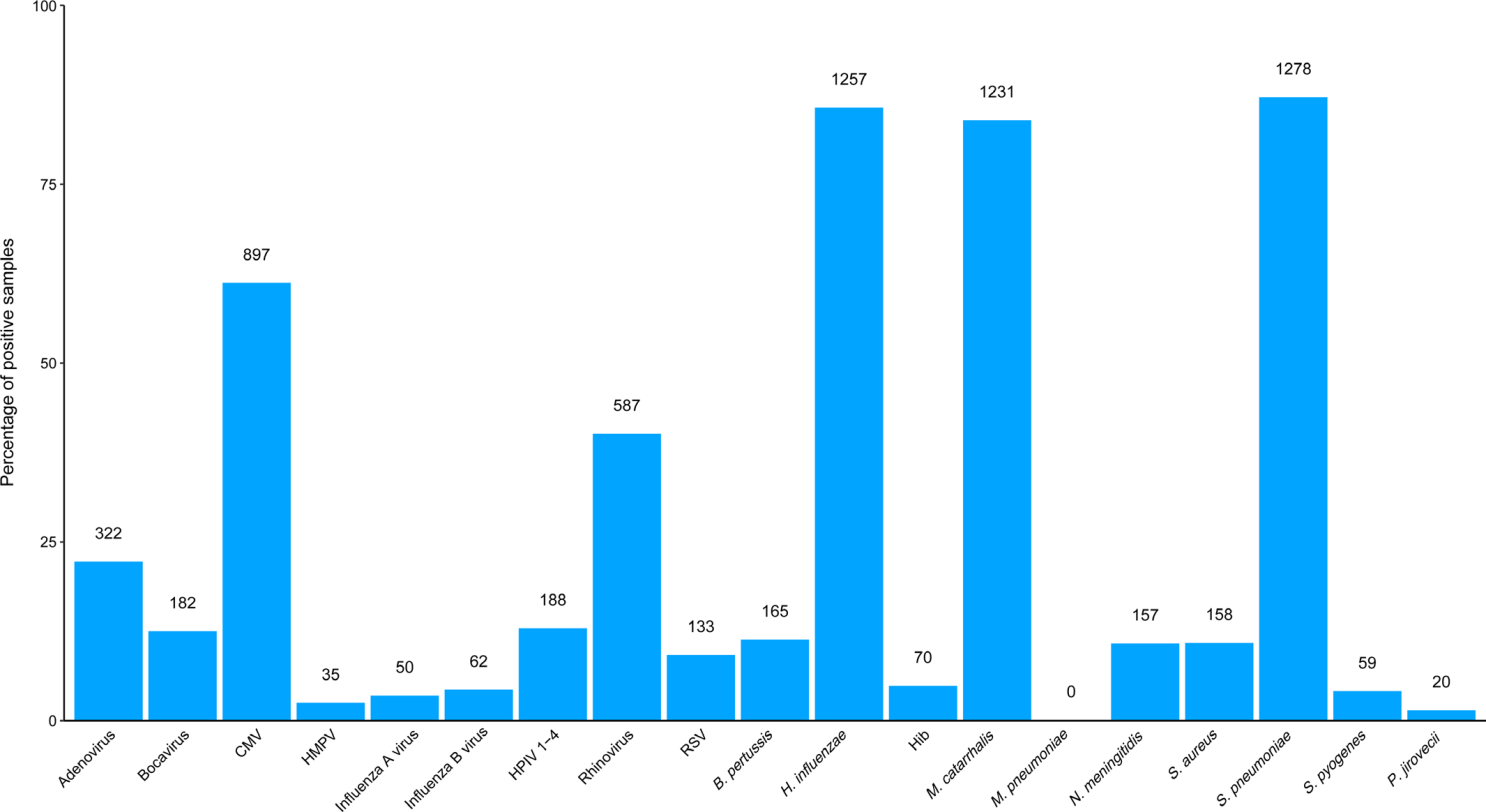
Prevalence of positive nasopharyngeal swab samples among 1468 children under 5 years of age who presented for care at the clinic at years 0, 1 and 2 in the Macrolides Oraux pour Réduire les Décès avec un Oeil sur la Résistance I trial.

We tested nasopharyngeal swabs from 1468 individual children from 166 communities (n=443 children from 83 azithromycin-treated communities; n=400 children from 71 placebo-treated communities; and n=625 children from 12 non-study communities). Swabs were collected at community clinics at three time points over a 2-year period: year 0, n=594 children; year 1, n=317 children; and year 2, n=557 children. Over half of the children were male (n=764/1462) with an average age of 1 year (mean=0.97 years; SD=1.16 years). Across all three time points and 1468 children, 87.1% of swabs were positive for *S. pneumoniae* (1278/1468), 85.6% for *H. influenzae* (1257/1468), 83.9% for *M. catarrhalis* (1231/1468), 61.1% for CMV (897/1468), 40.0% for rhinovirus (587/1467) and 22.1% for adenovirus (322/1455). All other pathogens were detected from less than 15% of swabs (figure 1 and online supplemental figure 1).

**Figure 2:**
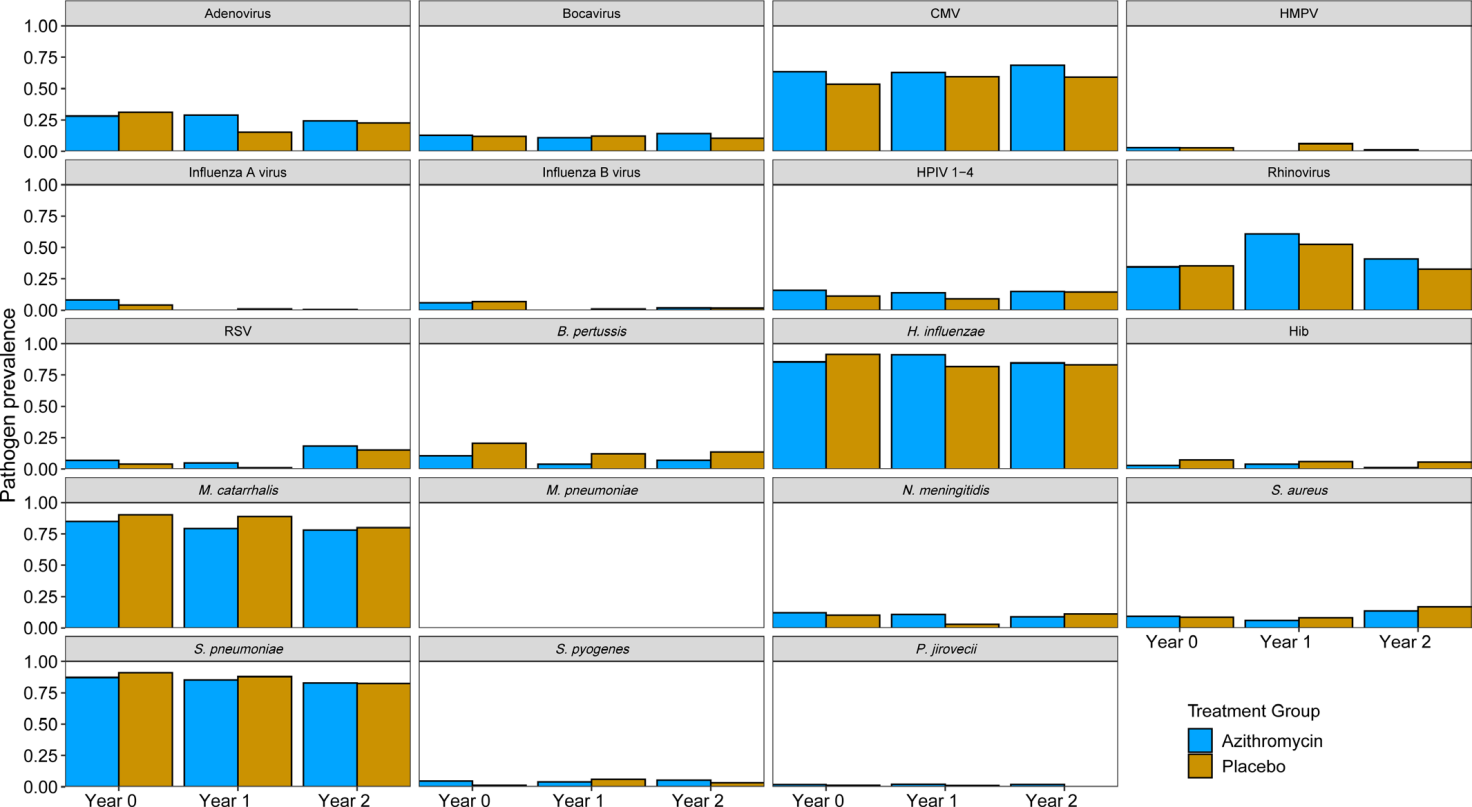
Proportion of positive nasopharyngeal swab samples by treatment and visit groups among 843 children under 5 years of age who presented for care at the clinic at years 0, 1 and 2 in the Macrolides Oraux pour Réduire les Décès avec un Oeil sur la Résistance I trial.

Across all three time points, children from the treatment communities had a 49% (prevalence ratio 0.51; 95% CI 0.35, 0.75) relative reduction of having a *B. pertussis* positive nasopharyngeal swab compared with children from the control communities, which corresponds to an absolute 8% (prevalence difference −0.08, 95% CI −0.12 to –0.03) reduction in *B. pertussis* among the treatment communities (table 1). Additionally, children in the treatment communities were 65% less likely (prevalence ratio 0.35; 95% CI 0.17, 0.71) to have a swab positive for Hib, corresponding to an absolute 5% reduction in Hib. The reduction in prevalence was consistent across all three time points (for *B. pertussis* (year 0 10.5 vs 20.5, year 1 3.9 vs 12.1 and year 2 7.1 vs 13.6); for Hib (year 0 2.9 vs 7.4, year 1 3.9 vs 6.1 and year 2: 1.2 vs 5.6)) (figure 2). When present, the quantities of these two pathogens were also lower: children from the treatment communities had 0.21 (quantity difference −0.21, 95% CI −0.32 to –0.10) fewer log10 copies of *B. pertussis* and 0.14 (quantity difference −0.14, 95% CI −0.25 to –0.02) fewer log10 copies of Hib compared with children from control communities. There was a modest increase in the prevalence ratio for CMV and RSV in the nasopharynx among children from azithromycin communities (prevalence ratio for CMV 1.13, 95% CI 1.01, 1.26; for RSV 1.56, 95% CI 1.00 to 2.43) and a slight decrease in the ratio and quantity of *M. catarrhalis* (prevalence ratio 0.94, 95% CI 0.89, 1.00). Otherwise, there were no statistically significant changes in the prevalence of other pathogens. Treatment effects were similar for children less than and older than 1 year of age (data not shown).

**Table 1:** Relative prevalence and quantity of positive nasopharyngeal swab samples of 19 respiratory pathogens among 843 children under 5 years of age who presented for care with respiratory symptoms at years 0, 1 and 2 in the Macrolides Oraux pour Réduire les Décès avec un Oeil sur la Résistance I trial

Pathogen	Prevalence ratios (95% CI) azithromycin vs placebo communities	Prevalence differences(95% CI) azithromycin vs placebo communities	Quantity differences among positive detections azithromycin vs placebo communities
Adenovirus	1.05 (0.83, 1.34)	0.01 (−0.05, 0.07)	0.04 (−0.12, 0.20)
Bocavirus	1.06 (0.73, 1.53)	0.01 (−0.04, 0.05)	−0.04 (−0.15, 0.07)
CMV	1.13 (1.01, 1.26)	0.07 (0.00, 0.14)	0.23 (0.05, 0.41)
HMPV	0.76 (0.33, 1.75)	−0.01 (−0.02, 0.01)	−0.01 (−0.06, 0.05)
Influenza A virus	1.70 (0.71, 4.08)*	0.01 (−0.01, 0.04)	0.02 (−0.07, 0.1)
Influenza B virus	0.81 (0.39, 1.68)	−0.01 (−0.03, 0.02)	−0.03 (−0.10, 0.04)
HPIV 1–4	1.23 (0.86, 1.75)	0.03 (−0.02, 0.08)	0.03 (−0.11, 0.17)
Rhinovirus	1.06 (0.90, 1.25)	0.02 (−0.04, 0.09)	0.04 (-0.12, 0.20)
RSV	1.56 (1.00, 2.43)	0.04 (0.00, 0.08)	0.08 (-0.06, 0.22)
*B.pertussis*	0.51 (0.35, 0.75)	−0.08 (−0.12, –0.03)	−0.21 (−0.32, –0.10)
*H.influenzae*	0.98 (0.93, 1.04)	−0.01 (−0.06, 0.03)	0.04 (−0.20, 0.28)
Hib	0.35 (0.17, 0.71)	−0.05 (−0.07, –0.02)	−0.14 (-0.25, –0.02)
*M.catarrhalis*	0.94 (0.89, 1.00)	−0.05 (−0.10, 0.00)	−0.23 (−0.45, –0.01)
*M.pneumoniae*	–	–	–
*N.meningitidis*	1.14 (0.75, 1.74)	0.01 (−0.03, 0.05)	0.05 (−0.06, 0.16)
*S.aureus*	0.86 (0.58, 1.27)	−0.02 (−0.06, 0.03)	−0.03 (−0.10, 0.04)
*S.pneumoniae*	0.97 (0.92, 1.03)	−0.03 (−0.07, 0.02)	0.01 (−0.18, 0.20)
*S.pyogenes*	1.72 (0.83, 3.57)	0.02 (−0.01, 0.04)	0.04 (0.00, 0.09)
*P.jirovecii*	2.31 (0.63, 8.42)	0.01 (0.00, 0.02)	0.01 (−0.01, 0.02)

Results adjusted for visit (years 0, 1, and 2) and month of swab collection. Adjustment for visit and month of swab collection was not possible because of Influenza A seasonality.

* Adjustment for visit and month of swab collection was not possible because of Influenza A seasonality.

CMVcytomegalovirusHib*Haemophilus influenzae* type BHMPVhuman metapneumovirusHPIVhuman parainfluenza 1–4RSVrespiratory syncytial virus

To contextualise these differences in pathogen detection, we extrapolated nasopharyngeal pathogen quantities and their specific associations with illness using the quantity-specific associations observed in the large case-control PERCH study of pneumonia aetiology.^6^ This analysis revealed that overall, the leading causes of respiratory illness in this substudy were rhinovirus (attributable fraction (AF) 16%), *H. influenzae* (AF 15%), human parainfluenza virus 1–4 (AF 10%), *B. pertussis* (AF 8%), RSV (8%) and *S. pneumoniae* (AF 6%; see table 2). Most pathogen attributable fractions were similar between the azithromycin and placebo communities, except for decreases in *B. pertussis* (AF 5% vs 11%) and increases in RSV (AF 10% vs 6%) in the azithromycin communities.

**Table 2:** Pathogen attributable fraction among 843 children under 5 years of age who presented for care at a clinic with respiratory symptoms at years 0, 1 and 2 in the Macrolides Oraux pour Réduire les Décès avec un Oeil sur la Résistance I trial

Pathogen	Azithromycin community children N (%)n=443	Placebocommunity children N (%)n=400	OverallN (%)n=843	OR (95% CI)*	Azithromycin communities AF†	Placebo communitiesAF†	OverallAF†
Adenovirus	117 (26.8)	97 (24.5)	214 (25.7)	1.16 (1.00, 1.34)	0.04	0.03	0.04
Bocavirus	57 (12.9)	46 (11.5)	103 (12.2)	1.21 (1.05, 1.39)	0.02	0.02	0.02
CMV	289 (65.2)	227 (56.8)	516 (61.2)	0.83 (0.73, 0.93)	–	–	–
HMPV	7 (1.6)	11 (2.8)	18 (2.1)	4.58 (3.62, 5.80)	0.01	0.02	0.02
Influenza A virus	15 (3.4)	8 (2.0)	23 (2.7)	3.30 (2.34, 4.64)	0.02	0.01	0.02
Influenza B virus	13 (2.9)	15 (3.8)	28 (3.3)	2.76 (1.65, 4.61)	0.02	0.02	0.02
HPIV 1–4	74 (16.7)	56 (14.0)	130 (15.4)	2.62 (2.22, 3.10)	0.10	0.09	0.10
Rhinovirus	190 (43)	155 (38.8)	345 (41)	1.61 (1.43, 1.81)	0.16	0.15	0.16
RSV	48 (10.9)	27 (6.8)	75 (8.9)	12.59 (10.49, 15.12)	0.10	0.06	0.08
*B.pertussis*	34 (7.7)	65 (16.2)	99 (11.7)	3.20 (1.65, 6.18)	0.05	0.11	0.08
*H.influenzae* ‡	330 (74.5)	282 (70.5)	612 (72.6)	1.25 (1.11, 1.41)	0.15	0.14	0.15
Hib‡	7 (1.6)	17 (4.2)	24 (2.8)	2.16 (1.23, 3.80)	0.01	0.02	0.02
*M.catarrhalis*	359 (81.0)	347 (86.8)	706 (83.7)	0.60 (0.54, 0.67)	–	–	–
*M.pneumoniae*	0 (0.0)	0 (0.0)	0 (0.0)	1.10 (0.69, 1.77)	0.00	0.00	0.00
*N.meningitidis* §	–	–	–	–	–	–	–
*S.aureus*	45 (10.2)	44 (11.0)	89 (10.6)	1.13 (0.98, 1.29)	0.01	0.01	0.01
*S.pneumoniae* ‡	73 (16.5)	67 (16.8)	140 (16.6)	1.59 (1.35, 1.88)	0.06	0.06	0.06
*S.pyogenes* §	–	–	–	–	–	–	–
*P.jirovecii* ‡	4 (0.9)	1 (0.2)	5 (0.6)	1.42 (1.07, 1.88)	0.00	0.00	0.00

* Derived from supplemental table 7 https://www.thelancet.com/cms/10.1016/S0140-6736(19)30721-4/attachment/6303e6bf-f391-4045-b893-0a85966ff700/mmc1.pdf.

† AF = (1-1/OR) * prevalence. AFs not calculated for ORs <1.

‡ Cutoffs applied with Ct values derived from copy numbers. For those without a published cutoff, Ct 35 was used.

§ Pathogen was not part of the PERCH study.

AFattributable fractionCMVcytomegalovirusCtcycle thresholdHib*Haemophilus influenzae* type BHMPVhuman metapneumovirusHPIVhuman parainfluenza 1–4PERCHPneumonia Etiology Research for Child HealthRSVrespiratory syncytial virus

## Discussion

In this study, we examined respiratory pathogens in children with respiratory illness from Niger. We found there were substantial reductions in the prevalence of *B. pertussis* and Hib in the azithromycin treated communities. These pathogens were detected in approximately 16% and 7% of cases of respiratory illness, respectively, in placebo communities versus 8% and 3% of cases from azithromycin communities. These pathogens are generally considered clinically significant and aetiologic during respiratory illness since they are infrequently carried asymptomatically in the nasopharynx.^10^ These pathogens can have high severity and mortality in infants and are also vaccine-preventable. The Nigerien pertussis and Hib vaccine coverage from 2015 to 2017 was reported to be 80–85%; however, some reports suggest lower coverage of all three doses.^14^ We do not know the vaccine status of children in this substudy, but it is possible that children in these communities were under-vaccinated or did not mount an effective response to vaccination, on which azithromycin provided an additional layer of protection from respiratory illness. This substudy did not measure or follow these individual children, so we did not directly assess mortality. However, these data suggest that reductions in *B. pertussis* and Hib may play a role in the benefit of azithromycin in high child mortality settings. As human-only pathogens, it is possible that azithromycin exerted a direct and/or indirect reduction in the childhood reservoir of *B. pertussis* and Hib.

After applying a quantitative analysis to the nasopharyngeal quantities of pathogens to better ascribe aetiology,^10^ the six leading pathogens were estimated to be rhinovirus, *H. influenzae*, parainfluenza virus, *B. pertussis*, *S. pneumoniae* and RSV. This epidemiology of respiratory illness, using advanced molecular tools and quantitative analysis, is important to highlight from high childhood mortality settings such as this and will be important to follow over time as azithromycin is deployed. This hierarchy is similar to other pneumonia aetiology studies,^9 10^ except the comparatively high prevalence of *B. pertussis* and low prevalence of RSV. This may reflect that the inclusion criteria was cough or difficulty breathing which could include upper respiratory tract infections such as *B. pertussis*, whereas RSV is more common with stricter pneumonia/lower respiratory tract case definitions.

That said, the high prevalence of *B. pertussis* was particularly notable. A high prevalence of pertussis has been reported previously in the paediatric ward setting in Niamey, Niger (11%), including fully, partially and unvaccinated children, and has not been associated with the classical symptoms of paroxysmal coughing or inspiratory whoop.^15^ It is known that pertussis is spread person to person, carries a high mortality in infants and presents even more non-specifically in the youngest age groups. While this substudy did not capture individual level outcome or mortality data, we believe this reduction of pertussis respiratory illness would predict a reduction in pertussis mortality in azithromycin communities. Our findings would recommend examining pertussis vaccine uptake and efficacy in such settings. Similarly, Hib causes both respiratory illness and meningitis; thus, the reduction in Hib detection in the nasopharynx would suggest that Hib meningitis was likely reduced. This would be compatible with the reduction in meningitis that was noted in verbal autopsy studies.^4^ Interestingly there was no difference in meningococcal carriage observed in azithromycin communities.

There were also no effects seen for the important respiratory pathogens *S. pneumoniae* or non-Hib *H. influenzae*. While treatable with azithromycin, these pathogens are carried in the nasopharynx with a high prevalence. It is possible that there was truly no effect of azithromycin on illness with these pathogens, or it is also possible that an effect may have been obscured by high background carriage or reinfection. There was a very slight decrease in *M. catarrhalis* carriage in the azithromycin community children. There was little difference in viral detection in the nasopharynx except for a slight increase in the prevalence ratios for RSV and CMV and attributable fraction due to RSV. The explanation for this increase is not known but could include pathogen replacement or artefact. This study measured relative prevalence not incidence, and therefore the incidences of any pathogen including RSV are not clear.

There were several limitations to this study. As mentioned, this secondary study design tested consecutive cases of respiratory illness only for those children who presented to the health centre, and thus does not permit measurements of incidence. However, other studies of azithromycin in communities in neighbouring countries have shown a 15% reduction in the incidence of upper respiratory tract infection with azithromycin.^16^ Because sampling occurred after the start of the main MORDOR trial, there were no swabs collected prior to any distribution of azithromycin or placebo, and thus a comparison to baseline prevalence was not possible. As mentioned, individual-level data on vaccine uptake or azithromycin treatment were not collected. Finally, detecting pathogens in the nasopharynx does not clearly implicate disease. To be as stringent as possible, we used pathogen quantity cut-offs from large studies performed in similar settings to better ascribe aetiology. However, these cut-offs were derived among children with a strict definition of pneumonia rather than among children with the case definition used in this study.

## Conclusion

In summary, we noted a reduction in the prevalence of *B. pertussis* and Hib in communities that received biannual mass distributed azithromycin. This suggests that these infections may be important causes of childhood mortality in Niger and that mass azithromycin may complement protection against vaccine-preventable infections in high mortality settings.

## supplementary material

10.1136/bmjgh-2024-016043 online supplemental file 1

10.1136/bmjgh-2024-016043 online supplemental file 2

## Data Availability

All data relevant to the study are included in the article or uploaded as supplementary information.
