# Supplementary material for: Effect of biannual azithromycin on respiratory pathogens among symptomatic children: results from the randomised Macrolides Oraux pour Réduire les Décès avec un Oeil sur la Résistance (MORDOR) I trial
Source: BMJ Glob Health. 2025 Feb 10;10(2):e016043. doi: 10.1136/bmjgh-2024-016043 (PMC11815404; doi:10.1136/bmjgh-2024-016043)

Supplemental Materials

Table S1 qPCR assays included on the customized TaqMan Array Card for the detection of respiratory pathogens.

Type	Organism	Target gene	Reference
Virus	Adenovirus	Hexon	(1-3)
	Bocavirus	NS1	(4)
		NP-1	(4)
	Cytomegalovirus	*gB*	(5, 6)
		IE2 exon 5	(5, 6)
	Human metapneumovirus	F	(2)
	Human parainfluenza 1	HN	(7)
	Human parainfluenza 2	HN	(2)
	Human parainfluenza 3	HN	(2)
	Human parainfluenza 4	HN	(7)
	Influenza A	M	(8)
	Influenza B	NP	(9)
	Respiratory syncytial virus	M	(2, 10)
	Rhinovirus	5’NCR	(2, 11)
Bacteria	*B pertussis*	IS481a	(2, 12)
	Haemophilus influenzae	*bexA*	(2, 13)
		hpd	(13)
	Haemophilus influenzae type B	*bcs2*	(14)
		bcs3	(15)
	Moraxella catarrhalis	*purH*	(16)
	Mycoplasma pneumoniae	CARDS toxin	(17, 18)
	Neisseria meningitidis	*sodC*	(18, 19)
	Staphylococcus aureus	Glutamate synthase	(20)
		sodA	(21)
	Streptococcus pneumoniae	*lytA*	(2, 22)
	Streptococcus pyogenes	spy1258	(2)
Fungus	*Pneumocystis jirovecii*	DHPS	(16)

Reference

1. Liu J, Gratz J, Amour C, Kibiki G, Becker S, Janaki L, et al. A Laboratory-Developed TaqMan Array Card for Simultaneous Detection of 19 Enteropathogens. J Clin Microbiol. 2013;51(2):472-80.

2. Kodani M, Yang GY, Conklin LM, Travis TC, Whitney CG, Anderson LJ, et al. Application of TaqMan Low-Density Arrays for Simultaneous Detection of Multiple Respiratory Pathogens. J Clin Microbiol. 2011;49(6):2175-82.

3. Heim A, Ebnet C, Harste G, Pring-Åkerblom P. Rapid and quantitative detection of human adenovirus DNA by real-time PCR (vol 70, pg 228, 2003). J Med Virol. 2003;71(2):320-.

4. Lu XY, Chittaganpitch M, Olsen SJ, Mackay IM, Sloots TP, Fry AM, et al. Real-time PCR

assays for detection of bocavirus in human specimens. J Clin Microbiol. 2006;44(9):3231-5.
5. Moore CC, Jacob ST, Banura P, Zhang JX, Stroup S, Boulware DR, et al. Etiology of Sepsis in Uganda Using a Quantitative Polymerase Chain Reaction-based TaqMan Array Card. Clin Infect Dis. 2019;68(2):266-72.
6. Boppana SB, Ross SA, Shimamura M, Palmer AL, Ahmed A, Michaels MG, et al. Saliva Polymerase-Chain-Reaction Assay for Cytomegalovirus Screening in Newborns. New Engl J Med. 2011;364(22):2111-8.
7. Weinberg GA, Schnabel KC, Erdman DD, Prill MM, Iwane MK, Shelley LM, et al. Field evaluation of TaqMan Array Card (TAC) for the simultaneous detection of multiple respiratory viruses in children with acute respiratory infection. J Clin Virol. 2013;57(3):254-60.
8. Malhotra B, Swamy MA, Reddy PVJ, Kumar N, Tiwari JK. Evaluation of custom multiplex real - time RT - PCR in comparison to fast - track diagnostics respiratory 21 pathogens kit for detection of multiple respiratory viruses. Virol J. 2016;13.
9. Chen Y, Cui DW, Zheng SF, Yang SG, Tong J, Yang DG, et al. Simultaneous Detection of Influenza A, Influenza B, and Respiratory Syncytial Viruses and Subtyping of Influenza A H3N2 Virus and H1N1 (2009) Virus by Multiplex Real-Time PCR. J Clin Microbiol. 2011;49(4):1653-6.
10. Fry AM, Chittaganpitch M, Baggett HC, Peret TCT, Dare RK, Sawatwong P, et al. The Burden of Hospitalized Lower Respiratory Tract Infection due to Respiratory Syncytial Virus in Rural Thailand. Plos One. 2010;5(11).
11. Lu XY, Holloway B, Dare RK, Kuypers J, Yagi S, Williams JV, et al. Real-time reverse transcription-PCR assay for comprehensive detection of human rhinoviruses. J Clin Microbiol. 2008;46(2):533-9.
12. Tatti KM, Wu KH, Tondella ML, Cassiday PK, Cortese MM, Wilkins PP, et al. Development and evaluation of dual-target real-time polymerase chain reaction assays to detect spp. Diagn Micr Infec Dis. 2008;61(3):264-72.
13. Wang X, Mair R, Hatcher C, Theodore MJ, Edmond K, Wu HM, et al. Detection of bacterial pathogens in Mongolia meningitis surveillance with a new real-time PCR assay to detect Haemophilus influenzae. Int J Med Microbiol. 2011;301(4):303-9.
14. Wang LP, Yuan Y, Liu YL, Lu QB, Shi LS, Ren X, et al. Etiological and epidemiological features of acute meningitis or encephalitis in China: a nationwide active surveillance study. Lancet Reg Health-W. 2022;20.
15. Marty A, Greiner O, Day PJR, Gunziger S, Mühlemann K, Nadal D. Detection of Haemophilus influenzae type b by real-time PCR. J Clin Microbiol. 2004;42(8):3813-5.
16. Nyawanda BO, Njuguna HN, Onyango CO, Makokha C, Lidechi S, Fields B, et al. Comparison of respiratory pathogen yields from Nasopharyngeal/Oropharyngeal swabs and sputum specimens collected from hospitalized adults in rural Western Kenya. Sci Rep-Uk. 2019;9.
17. Winchell JM, Thurman KA, Mitchell SL, Thacker WL, Fields BS. Evaluation of three real-time PCR assays for detection of Mycoplasma pneumoniae in an outbreak investigation. J Clin Microbiol. 2008;46(9):3116-8.
18. Onyango CO, Loparev V, Lidechi S, Bhullar V, Schmid DS, Radford K, et al. Evaluation of a TaqMan Array Card for Detection of Central Nervous System Infections. J Clin Microbiol. 2017;55(7):2035-44.

19. Thomas JD, Hatcher CP, Satterfield DA, Theodore MJ, Bach MC, Linscott KB, et al. -Based Real-Time PCR for Detection of. Plos One. 2011;6(5).
20. Diaz MH, Waller JL, Napoliello RA, Islam MS, Wolff BJ, Burken DJ, et al. Optimization of Multiple Pathogen Detection Using the TaqMan Array Card: Application for a Population-Based Study of Neonatal Infection. Plos One. 2013;8(6).
21. Banada PP, Chakravorty S, Shah D, Burday M, Mazzella FM, Alland D. Highly Sensitive Detection of
Directly from Patient Blood. Plos One. 2012;7(2).
22. Carvalho MDS, Tondella ML, McCaustland K, Weidlich L, McGee L, Mayer LW, et al. Evaluation and improvement of real-time PCR assays targeting lytA, ply, and psaA genes for detection of pneumococcal DNA. J Clin Microbiol. 2007;45(8):2460-6.

Supplemental Figure 1. Prevalence of positive nasopharyngeal swab samples among 1468 children under 5 years of age who presented for care at the clinic at years 0, 1, and 2 in the MORDOR I trial, stratified by community (N=443 children from 83 azithromycin-treated communities; N=400 children from 71 placebo-treated communities; and N=625 children from 12 non-study communities)

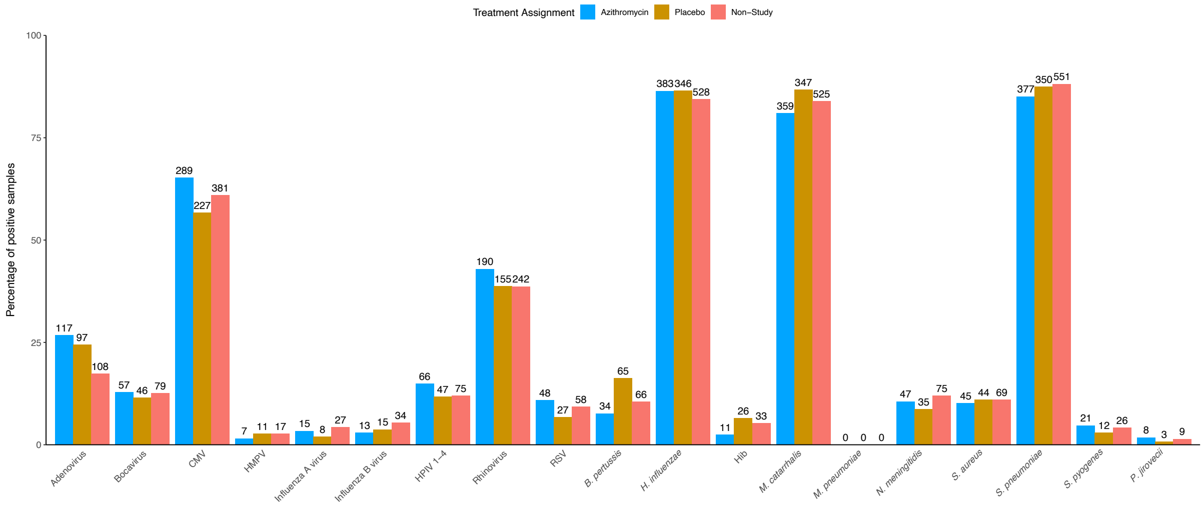

Supplement: online supplemental file 1 [file bmjgh-10-2-s001.pdf]
