# Supplementary material for: Effect of biannual azithromycin on respiratory pathogens among symptomatic children: results from the randomised Macrolides Oraux pour Réduire les Décès avec un Oeil sur la Résistance (MORDOR) I trial
Source: BMJ Glob Health. 2025 Feb 10;10(2):e016043. doi: 10.1136/bmjgh-2024-016043 (PMC11815404; doi:10.1136/bmjgh-2024-016043)
Supplement: online supplemental file 2 [file bmjgh-10-2-s002.pdf]

LabID	group	visit	adenovirus	bocavirus	cmv	hmpv	influenzaA	influenzaB	parainflu1_4	rhinovirus	rsv	b_pertussis	h_influenzae.hpd.	h_influenzaeB	m_catarhalis	m_pneumoniae	n_meningitidis	s_aureus	s_pneumoniae	s_pyogenes	p_jirovecii
1	azithro	12months	35	35	25.057	35	35	35	35	26.091	35	35	28.009	35	22.08	35	35	35	26.806	35	35
2	placebo	0months	35	35	26.676	35	35	35	35	23.865	35	35	19.223	34.287	25.779	35	35	35	20.151	35	35
3	nonstudy	24months	31.822	35	35	35	35	35	19.749	29.54	35	33.939	34.941	35	22.002	35	35	35	35	35	35
4	nonstudy	0months	31.685	35	27.303	35	35	35	35	35	35	35	19.702	35	22.44	35	35	35	24.648	35	35
5	placebo	24months	35	35	35	35	35	35	18.669	32.943	35	35	35	35	35	35	32.058	35	30.017	35	35
6	nonstudy	0months	35	35	35	35	35	35	35	29.587	35	35	35	35	24.927	35	35	26.849	26.156	35	35
7	nonstudy	24months	35	35	34.917	35	35	35	35	29.027	35	32.386	27.295	35	35	35	25.769	35	26.517	35	35
8	azithro	24months	35	32.103	35	35	35	35	35	31.454	35	35	21.73	35	35	35	35	35	35	32.827	35
9	placebo	0months	32.96	35	29.408	35	35	35	35	35	35	35	24.463	35	33.229	35	35	35	35	35	35
10	azithro	0months	35	35	35	35	35	35	35	35	35	35	20.739	20.483	23.503	35	35	35	29.363	35	35
11	azithro	24months	35	35	19.319	35	35	35	35	35	35	35	33.716	35	24.44	35	35	35	26.205	35	35
12	nonstudy	24months	35	35	30.869	35	35	35	35	35	16.411	35	24.259	35	23.559	35	35	35	23.532	35	35
13	azithro	24months	31.611	35	31.576	35	35	35	35	24.664	35	35	19.546	35	25.808	35	35	35	26.366	35	35
14	nonstudy	0months	27.068	35	31.932	35	35	35	35	35	35	35	20.525	20.381	23.912	35	35	35	24.175	35	35
15	nonstudy	0months	35	35	29.434	35	35	35	35	35	35	35	35	35	22.192	35	35	35	25.124	35	35
16	nonstudy	24months	35	35	35	35	35	35	20.112	35	35	35	25.765	35	20.852	35	35	35	24.997	35	35
17	placebo	24months	30.528	35	35	35	35	35	35	35	19.269	35	19.827	35	23.063	35	35	35	25.538	35	35
18	nonstudy	12months	35	35	21.711	35	35	35	35	24.09	35	35	19.336	18.99	22.678	35	35	35	22.301	35	35
19	placebo	0months	33.224	35	35	35	35	35	35	35	35	28.528	19.048	35	22.278	35	35	35	25.183	35	35
20	azithro	0months	29.905	30.909	35	35	35	35	35	27.237	35	35	21.003	35	22.317	35	35	35	24.619	35	35
21	placebo	0months	29.472	35	32.417	35	35	35	35	35	35	35	29.09	35	21.773	35	35	35	27.757	35	35
22	azithro	24months	35	35	35	35	35	35	35	35	35	35	35	35	35	35	35	35	35	35	35
23	nonstudy	0months	35	35	31.947	35	35	35	35	35	35	35	18.77	35	17.564	35	35	35	22.744	35	35
24	azithro	24months	35	35	33.914	35	35	35	35	24.719	21.321	35	21.03	35	35	35	35	35	19.984	35	35
25	nonstudy	24months	26.585	35	28.973	35	35	35	35	34.259	35	35	21.77	35	22.393	35	33.383	35	26.228	35	35
26	placebo	0months	35	35	30.958	35	35	35	20.903	35	35	35	19.256	35	26.051	35	35	26.587	22.274	35	35
27	placebo	12months	35	35	25.027	35	35	35	35	31.187	35	35	21.586	26.4	25.681	35	35	35	26.331	35	35
28	nonstudy	0months	35	35	35	35	35	35	35	35	35	27.228	35	35	35	35	35	35	35	35	35
29	nonstudy	12months	35	35	35	35	35	35	35	35	35	35	35	35	35	35	35	35	35	35	35
30	azithro	12months	35	35	35	35	35	35	35	30.067	35	35	35	35	35	35	35	35	26.649	35	35
31	nonstudy	24months	35	35	28.425	35	35	35	35	31.924	35	35	23.095	35	22.964	35	35	35	25.255	35	35
32	azithro	0months	35	35	24.159	35	35	35	35	25.259	35	35	21.194	35	25.24	35	35	35	25.023	35	32.084
33	nonstudy	0months	35	35	35	35	18.313	35	35	33.607	35	35	26.711	35	22.62	35	35	35	24.18	35	35
34	placebo	0months	35	35	28.759	35	35	35	35	35	35	35	22.582	35	22.765	35	35	34.917	24.674	35	35
35	placebo	24months	14.938	35	33.99	35	35	35	35	35	34.809	35	24.258	23.088	25.553	35	35	35	27.333	35	35
36	azithro	0months	27.708	35	29.545	35	35	35	35	35	35	35	17.763	35	22.743	35	35	35	24.858	35	35
37	azithro	24months	35	35	23.831	35	35	35	35	35	35	35	20.024	35	31.242	35	35	35	24.074	35	35
38	nonstudy	0months	35	35	35	35	35	35	35	35	35	31.192	35	35	35	35	35	35	35	35	35
39	nonstudy	12months	35	35	35	35	35	35	35	35	35	35	35	35	35	35	35	35	33.627	35	35
40	nonstudy	24months	29.821	35	35	35	35	35	35	29.262	35	35	22.331	35	22.14	35	35	35	24.441	35	35
41	placebo	0months	35	35	35	35	35	35	35	29.939	35	35	25.833	35	21.899	35	35	35	23.335	35	35
42	nonstudy	24months	35	35	35	35	35	35	35	35	35	35	20.532	35	20.154	35	35	35	27.318	35	35
43	placebo	0months	35	35	27.41	35	35	35	35	35	35	35	21.135	35	20.742	35	35	35	23.787	35	35
44	nonstudy	24months	35	35	35	35	35	35	35	32.726	35	35	23.971	35	30.179	35	35	35	28.917	35	35
45	nonstudy	24months	35	35	24.741	35	35	35	35	29.732	16.663	35	19.378	35	19.833	35	33.772	26.897	22.169	35	35
46	placebo	0months	35	35	34.474	19.137	35	35	35	35	35	35	16.255	35	19.388	35	35	35	19.201	32.648	35
47	azithro	24months	35	35	29.926	35	35	35	35	23.918	35	35	21.76	35	22.725	35	35	35	24.008	35	35
48	placebo	12months	35	35	35	35	35	35	35	34.137	35	35	35	35	22.631	35	35	35	24.104	35	35
49	nonstudy	12months	35	35	32.913	35	35	35	35	35	35	35	35	35	24.947	35	35	27.28	28.445	35	35
50	nonstudy	24months	35	35	26.019	35	35	35	35	20.774	35	35	21.33	35	24.717	35	35	35	23.932	35	35
51	nonstudy	0months	35	35	35	35	35	35	35	35	35	35	22.055	35	21.202	35	35	35	24.526	35	35
52	nonstudy	0months	35	35	24.096	35	35	35	35	33.453	35	35	22.63	35	21.62	35	35	35	25.638	35	35
53	placebo	0months	32.151	35	35	33.306	35	35	35	35	35	35	17.374	35	17.57	35	35	33.513	19.807	35	35
54	placebo	0months	21.61	35	35	35	35	35	31.121	35	35	35	24.168	35	25.899	35	22.827	35	22.527	35	35
55	azithro	24months	35	35	27.648	35	35	35	35	35	35	35	23.638	35	24.808	35	35	35	31.699	35	35
56	azithro	24months	35	35	27.781	35	35	30.377	35	35	35	35	20.313	35	21.015	35	35	35	23.025	35	35
57	nonstudy	24months	35	35	29.934	35	35	35	35	27.162	35	27.289	18.407	35	25.316	35	35	35	24.743	35	35
58	placebo	0months	35	35	35	35	35	35	29.954	35	35	35	19.577	35	19.955	35	35	35	29.993	35	35
59	nonstudy	24months	35	35	35	35	35	35	35	35	35	35	35	35	33.931	35	35	26.483	35	35	35
60	placebo	0months	35	35	35	34.221	35	35	35	31.277	35	35	34.927	35	24.484	35	35	35	25.852	35	35
61	placebo	0months	35	35	35	35	35	35	35	30.764	35	17.007	22.118	35	19.539	35	35	35	25.033	35	35
62	nonstudy	0months	35	35	35	35	35	35	35	29.718	35	35	19.379	35	18.422	35	34.197	35	19.869	35	35
63	nonstudy	12months	35	35	34.776	35	35	35	35	35	35	35	25.848	35	34.337	35	35	35	23.906	35	35
64	azithro	12months	33.217	35	35	35	35	35	34.26	31.72	35	35	22.602	35	20.195	35	30.522	35	23.736	35	35

65	azithro	24months	35	35	29.29	35	35	35	35	35	35	35	25.634	35	21.652	35	35	35	35	35	35
66	azithro	12months	35	35	35	35	35	35	35	24.68	35	35	27.614	35	26.846	35	35	35	27.799	35	35
67	nonstudy	12months	35	35	29.074	35	35	35	35	35	35	35	35	35	35	35	35	35	28.605	35	35
68	azithro	0months	23.873	35	23.742	30.962	35	35	35	35	35	35	26.512	35	23.132	35	19.33	35	25.094	35	35
69	placebo	0months	35	35	35	35	35	35	35	35	35	35	23.383	35	21.233	35	35	35	22.526	35	35
70	azithro	24months	35	35	24.524	35	35	35	35	33.295	35	35	24.928	35	32.342	35	35	35	27.37	35	35
71	nonstudy	24months	35	35	31.4	35	35	35	35	29.367	35	35	25.271	35	31.457	35	35	35	29.571	35	35
72	placebo	24months	35	19.814	32.684	35	35	35	35	35	35	18.652	28.641	35	22.124	35	32.589	35	30.827	35	35
73	nonstudy	0months	26.183	35	31.303	35	35	35	35	20.036	35	34.094	26.375	35	18.608	35	35	35	20.024	35	35
74	azithro	0months	35	35	34.179	35	35	35	35	35	35	35	17.113	35	21.154	35	35	23.577	21.082	25.503	35
75	nonstudy	0months	35	35	35	35	35	30.029	35	35	35	31.863	35	35	35	35	35	35	35	35	35
76	nonstudy	12months	35	21.875	31.126	35	35	35	35	30.491	35	35	24.569	35	30.546	35	35	35	29.4	35	35
77	placebo	0months	35	35	35	35	35	35	35	35	35	35	23.128	35	21.004	35	35	35	25.929	35	35
78	placebo	24months	35	35	27.423	35	35	35	35	35	31.362	35	24.92	35	35	35	35	35	33.891	35	35
79	azithro	24months	35	35	27.126	35	35	35	35	22.385	35	35	19.642	35	25.626	35	35	35	21.825	35	35
80	azithro	24months	35	29.141	26.18	35	35	35	34.069	33.848	35	35	19.649	35	26.722	35	35	35	26.537	35	35
81	placebo	0months	35	35	28.022	34.026	35	35	35	31.488	35	35	23.855	35	21.959	35	30.146	35	24.66	35	35
82	azithro	12months	35	35	35	35	35	35	35	25.426	35	35	19.337	35	24.808	35	35	35	35	35	35
83	azithro	24months	35	35	35	35	35	35	35	34.552	32.597	35	21.445	35	23.388	35	19.341	35	24.979	35	35
84	azithro	0months	35	35	30.958	35	35	35	35	35	35	35	23.894	35	24.211	35	35	35	35	35	35
85	nonstudy	24months	35	35	29.788	35	35	35	23.352	35	35	35	24.326	35	23.347	35	35	35	24.668	35	35
86	nonstudy	24months	35	35	35	35	35	35	35	27.933	35	34.307	20.229	35	19.721	35	35	35	23.09	35	35
87	placebo	24months	35	35	35	35	35	35	35	28.022	35	25.231	23.613	35	35	35	35	28.77	35	35	35
88	nonstudy	12months	35	35	31.842	35	35	35	35	35	35	35	17.004	35	20.833	35	35	35	20.99	35	35
89	nonstudy	24months	35	35	35	35	35	35	35	35	35	35	35	35	24.395	35	35	35	27.444	35	35
90	placebo	0months	34.426	35	24.669	35	35	35	35	23.75	35	35	18.328	35	21.339	35	35	35	19.43	35	35
91	azithro	0months	35	35	35	35	35	35	35	27.126	35	35	20.033	35	24.021	35	34.222	35	23.548	35	35
92	placebo	12months	32.555	35	29.522	33.078	35	35	35	35	35	35	23.65	35	21.934	35	35	35	26.51	35	35
93	azithro	0months	35	35	35	35	35	35	35	24.319	35	35	20.24	35	35	35	35	35	34.238	35	35
94	placebo	0months	33.192	35	32.05	35	28.109	35	35	35	35	35	20.424	35	35	35	35	35	23.468	35	35
95	placebo	12months	35	33.535	35	35	35	35	25.708	35	35	35	18.501	35	22.104	35	35	35	22.795	35	35
96	placebo	0months	35	34.398	35	35	35	35	35	27.655	35	35	18.176	35	20.728	35	35	35	23.608	35	35
97	nonstudy	0months	35	35	29.034	35	35	35	30.972	35	35	35	22.65	35	20.957	35	35	35	23.002	35	35
98	nonstudy	24months	35	35	28.78	35	35	35	35	34.411	35	34.478	33.841	35	23.632	35	35	33.508	31.695	35	35
99	azithro	0months	35	34.557	35	35	35	35	35	35	35	35	21.894	35	21.964	35	35	35	21.713	35	35
100	placebo	24months	35	35	35	35	35	35	35	35	35	35	35	35	35	35	35	35	35	35	35
101	azithro	0months	35	35	29.887	35	35	35	19.366	35	35	35	26.031	35	22.083	35	35	35	25.209	35	35
102	nonstudy	12months	35	10.32	26.059	35	35	35	35	25.656	35	35	20.076	35	22.575	35	35	35	23.48	35	35
103	placebo	0months	34.717	34.381	35	35	35	32.375	35	35	35	35	18.359	35	21.945	35	35	35	21.484	35	35
104	nonstudy	24months	35	35	33.443	35	17.592	35	35	35	35	35	24.252	35	22.862	35	31.266	35	28.771	35	35
105	nonstudy	0months	35	35	34.313	35	35	35	35	35	35	35	26.711	35	20.641	35	35	35	31.284	35	35
106	azithro	24months	35	35	30.425	35	35	35	35	35	16.925	35	20.259	35	21.385	35	20.738	34.437	23.911	35	35
107	nonstudy	12months	35	24.724	25.395	33.478	35	35	31.452	27.902	34.925	35	19.435	35	25.498	35	35	33.627	26.294	35	35
108	azithro	0months	35	35	35	35	35	35	35	27.38	35	35	19.547	35	35	35	35	35	35	35	35
109	nonstudy	24months	31.889	34.277	33.66	35	35	35	35	35	35	34.888	19.223	35	19	35	21.484	35	23.849	33.648	35
110	nonstudy	0months	35	34.512	23.969	35	35	35	35	35	35	35	20.17	35	23.885	35	35	35	24.348	35	35
111	placebo	0months	35	33.797	35	35	35	35	35	35	35	35	26.217	35	22.23	35	35	35	25.795	35	35
112	placebo	24months	35	35	35	35	35	35	35	35	35	35	19.632	35	17.138	35	35	35	21.63	35	35
113	azithro	24months	35	35	22.797	35	35	35	35	28.367	35	35	19.296	35	21.492	35	35	35	20.885	35	35
114	nonstudy	0months	35	35	35	35	35	35	35	29.418	35	35	25.614	35	23.489	35	35	35	22.6	35	35
115	nonstudy	0months	30.471	35	29.824	35	35	35	35	35	35	35	25.429	35	28.098	35	35	35	25.988	35	35
116	azithro	0months	15.305	35	25.454	35	35	35	35	25.116	35	35	26.089	35	20.52	35	35	35	22.179	35	35
117	nonstudy	24months	35	35	35	35	35	35	18.089	35	35	35	23.1	35	35	35	35	35	35	35	35
118	placebo	0months	35	35	35	35	35	33.972	35	35	35	30.508	35	35	35	35	35	35	35	35	35
119	azithro	24months	35	32.103	26.254	32.14	35	35	35	35	35	33.882	25.494	35	21.297	35	20.873	35	25.509	35	35
120	nonstudy	24months	35	35	31.307	35	35	35	35	35	35	35	22.344	35	35	35	35	35	35	35	35
121	placebo	0months	35	35	35	35	35	35	35	35	35	35	21.467	35	21.979	35	35	33.31	23.131	35	35
122	placebo	0months	35	35	28.527	35	35	35	35	35	35	35	17.851	35	20.035	35	35	35	23.223	35	35
123	azithro	24months	32.691	34.603	35	35	35	35	35	35	35	30.984	35	35	20.973	35	35	35	35	35	35
124	placebo	12months	35	32.182	23.1	35	35	35	35	35	35	35	33.744	35	25.324	35	35	35	28.829	35	35
125	azithro	24months	25.074	35	35	35	35	35	35	35	35	35	22.28	35	35	35	35	35	25.827	35	35
126	nonstudy	0months	35	35	35	35	21.486	35	35	35	35	35	19.418	35	21.421	35	20.183	35	26.861	35	35
127	azithro	12months	35	35	26.262	35	35	35	35	35	NA	35	22.131	35	35	35	35	35	35	35	35
128	azithro	24months	35	35	27.272	35	35	35	35	34.261	NA	35	35	35	35	35	35	35	29.57	35	35
129	nonstudy	12months	35	35	35	35	35	35	35	35	35	31.536	35	35	29.498	35	35	35	35	35	35
130	azithro	12months	35	35	28.407	35	35	35	35	29.035	35	35	28.781	35	21.521	35	24.765	35	35	35	35

131	nonstudy	24months	27.631	34.529	28.732	35	35	35	35	35	35	34.986	26.456	35	35	35	35	35	35	35	35
132	nonstudy	0months	34.695	33.624	28.61	35	35	35	35	29.426	NA	35	24.334	35	22.41	35	35	35	26.346	35	35
133	azithro	24months	14.913	33.216	28.799	35	35	35	35	35	35	35	24.566	35	24.725	35	35	35	35	35	35
134	nonstudy	0months	35	35	35	35	35	33.881	35	35	35	30.749	35	35	35	35	35	35	35	35	35
135	azithro	24months	27.315	35	31.41	35	35	35	35	35	35	35	21.651	35	24.304	35	35	35	23.394	35	35
136	azithro	24months	35	35	35	35	35	35	35	35	35	35	35	35	35	35	35	35	35	35	35
137	azithro	0months	35	35	35	35	35	35	35	35	19.828	35	20.605	35	24.378	35	35	32.745	24.959	35	35
138	azithro	0months	34.004	35	35	35	35	35	35	35	35	35	20.959	35	22.617	35	35	33.515	25.088	35	35
139	azithro	0months	35	35	35	35	35	35	35	26.774	35	35	19.964	35	22.812	35	35	35	21.433	35	35
140	nonstudy	12months	35	35	25.824	35	35	35	35	35	35	35	22.726	35	23.575	35	35	35	26.165	35	35
141	nonstudy	0months	35	35	35	35	35	35	35	35	35	35	21.061	21.457	23.268	35	35	35	24.355	35	35
142	nonstudy	24months	35	35	35	35	35	35	35	35	35	35	23.163	35	24.641	35	35	35	35	35	35
143	nonstudy	0months	35	35	26.919	35	35	35	35	29.162	35	35	21.72	35	24.308	35	35	35	25.603	35	35
144	placebo	24months	35	35	25.871	35	35	35	35	35	23.821	35	24.901	35	26.236	35	35	35	35	35	35
145	nonstudy	12months	35	35	35	35	35	35	35	30.667	35	34.683	25.584	35	20.807	35	35	35	35	31.945	35
146	azithro	0months	11.398	35	32.639	35	35	35	35	35	35	35	23.241	35	25.467	35	35	35	27.888	35	35
147	azithro	0months	33.434	35	33.446	35	35	35	35	35	35	35	25.384	35	35	35	35	35	25.128	35	35
148	nonstudy	24months	35	35	30.704	35	35	35	35	35	35	35	25.063	35	29.797	35	32.755	35	25.583	35	35
149	azithro	24months	34.249	35	28.675	35	35	35	35	31.01	35	35	24.459	35	20.854	35	35	35	25.739	35	35
150	azithro	12months	33.295	35	24.017	35	35	35	35	29.483	35	35	21.766	35	22.69	35	35	32.553	23.829	35	35
151	azithro	24months	33.584	35	29.821	35	35	35	35	30.514	35	35	21.329	35	29.303	35	35	35	27.644	35	35
152	nonstudy	0months	15.368	35	27.69	34.551	35	35	35	32.447	29.405	35	19.828	35	23.133	35	35	35	28.141	35	35
153	nonstudy	12months	35	31.495	25.311	35	35	35	35	29.74	35	35	19.408	35	21.132	35	35	33.663	23.894	35	35
154	azithro	24months	35	29.717	35	35	35	35	35	27.712	35	35	21.332	35	26.578	35	35	35	29.301	35	35
155	placebo	24months	30.957	26.407	35	35	35	35	30.659	27.701	35	35	21.822	35	27.066	35	35	35	26.908	35	35
156	nonstudy	24months	35	35	24.525	35	35	35	35	35	35	29.919	25.663	35	21.118	35	35	35	27.593	35	35
157	nonstudy	0months	35	35	35	35	35	35	35	35	35	32.389	19.711	19.089	21.926	35	35	35	27.697	35	35
158	nonstudy	24months	35	26.016	28.811	35	35	35	35	35	35	35	25.037	35	19.835	35	35	35	23.612	35	35
159	placebo	0months	17.142	35	33.675	35	35	35	35	35	35	35	21.841	35	21.212	35	35	35	25.885	35	35
160	nonstudy	24months	NA	35	34.528	35	35	35	34.55	27.652	35	35	19.684	35	26.252	35	35	35	25.074	35	35
161	placebo	24months	NA	35	32.191	35	35	35	35	35	21.941	35	22.158	35	31.76	35	35	35	26.676	35	35
162	placebo	12months	NA	35	33.316	35	35	35	35	35	35	35	21.857	35	27.852	35	35	35	27.046	35	35
163	azithro	0months	NA	35	28.462	35	35	35	35	31.881	35	35	21.402	35	24.473	35	35	35	24.467	35	35
164	nonstudy	0months	35	35	35	35	35	35	35	30.096	35	35	23.053	35	25.372	35	35	35	23.782	35	35
165	azithro	24months	NA	35	27.737	35	35	35	35	35	35	35	18.248	35	21.015	35	35	35	27.806	35	35
166	azithro	24months	NA	30.689	27.564	35	35	35	35	35	35	35	25.25	35	25.136	35	35	35	27.578	29.278	35
167	nonstudy	24months	NA	33.336	30.437	35	35	35	35	28.939	35	35	23.622	35	23.895	35	35	35	30.492	35	35
168	azithro	24months	NA	35	28.694	35	35	35	35	35	16.842	35	24.733	30.584	35	35	35	35	22.05	35	35
169	nonstudy	24months	35	35	33.212	35	35	18.245	35	35	35	35	19.403	35	21.262	35	23.389	35	23.965	35	35
170	nonstudy	0months	35	35	35	35	35	29.844	35	35	35	30.796	35	35	35	35	35	35	35	35	35
171	placebo	24months	35	35	35	35	35	35	35	27.567	35	35	33.842	35	35	35	35	35	35	35	35
172	nonstudy	12months	35	35	35	35	35	35	35	28.984	35	35	19.243	35	20.836	35	35	35	22.1	35	35
173	nonstudy	24months	35	35	35	35	35	35	35	35	35	35	35	35	35	35	35	35	35	35	35
174	nonstudy	12months	35	35	27.4	35	35	35	35	35	35	34.956	31.108	35	35	35	35	35	31.098	35	35
175	azithro	12months	NA	35	35	35	35	35	35	35	34.283	35	26.108	35	27.316	35	35	35	35	35	35
176	azithro	24months	NA	35	24.772	35	35	35	23.755	35	35	35	23.964	35	35	35	35	35	30.727	35	35
177	azithro	12months	35	35	35	35	35	35	35	35	35	31.856	35	35	35	35	35	35	35	35	35
178	placebo	12months	35	35	35	35	35	35	35	35	35	35	29.1	35	24.518	35	35	35	28.352	35	35
179	placebo	12months	35	35	28.302	35	35	35	35	35	35	35	18.01	35	24.533	35	35	34.494	23.549	34.799	35
180	nonstudy	0months	NA	35	30.118	35	35	35	35	35	35	33.453	21.135	35	22.943	35	35	35	24.975	35	35
181	placebo	0months	NA	35	30.689	35	35	35	35	35	35	35	21.901	35	22.64	35	35	34.62	24.601	35	35
182	placebo	0months	NA	35	33.707	35	35	35	35	35	35	30.066	35	35	22.865	35	35	35	27.568	35	35
183	placebo	24months	35	35	35	35	35	35	35	35	35	35	33.41	35	20.716	35	35	35	31.787	35	35
184	nonstudy	0months	35	35	27.985	35	35	35	35	35	35	35	26.26	35	22.023	35	35	35	25.17	35	35
185	placebo	12months	35	35	25.671	35	35	35	35	35	35	35	23.035	35	25.166	35	35	35	25.726	35	35
186	azithro	0months	31.975	33.214	25.617	35	35	35	33.488	33.884	33.479	35	19.228	35	25.208	35	35	35	25.955	35	35
187	azithro	12months	35	35	32.143	35	35	35	35	35	35	32.729	35	35	25.624	35	35	35	35	35	35
188	nonstudy	24months	30.761	14.338	35	35	35	35	35	35	35	35	18.737	35	22.302	35	35	35	24.59	35	35
189	azithro	24months	35	35	35	35	35	35	25.853	35	35	35	22.681	35	25.058	35	35	35	23.532	35	35
190	nonstudy	24months	35	19.274	35	35	35	35	24.384	32.927	35	35	21.116	35	35	35	35	35	35	35	35
191	nonstudy	24months	35	35	35	35	35	35	35	35	35	35	35	35	35	35	35	28.281	35	35	35
192	azithro	0months	10.635	33.348	32.772	35	35	35	35	30.869	35	35	20.549	35	33.654	35	35	35	33.004	35	35
193	placebo	12months	35	35	31.128	35	35	35	35	35	35	32.896	33.072	35	22.781	35	35	35	25.809	35	35
194	azithro	24months	25.825	34.389	34.946	35	35	35	35	34.708	33.983	35	28.08	35	34.606	35	35	35	35	35	35
195	placebo	12months	35	35	29.162	35	35	35	35	35	35	35	35	35	35	35	35	35	27.582	35	35
196	placebo	0months	35	34.486	35	35	35	35	35	27.797	35	35	21.002	35	35	35	35	35	23.783	35	35

197	azithro	0months	35	35	29.607	35	28.312	35	35	35	35	35	20.804	35	23.839	35	35	35	25.408	33.831	35
198	placebo	24months	35	35	33.388	35	35	35	35	35	35	35	25.192	35	34.395	35	35	35	35	35	35
199	placebo	24months	34.061	35	35	35	35	35	35	35	35	35	35	35	34.54	35	35	34.471	35	35	35
200	nonstudy	0months	35	35	35	35	35	35	32.631	34.395	35	35	24.871	35	22.968	35	35	35	25.681	35	35
201	nonstudy	24months	32.267	35	35	35	35	35	21.3	28.344	35	35	17.567	35	35	35	35	35	21.588	35	35
202	placebo	12months	35	35	35	35	35	35	35	35	35	35	28.943	35	35	35	35	35	29.289	35	35
203	placebo	12months	35	35	35	35	35	35	35	34.072	35	35	35	35	32.336	35	35	33.711	34.161	35	35
204	placebo	0months	27.598	35	35	35	35	35	33.305	35	35	35	19.637	35	23.76	35	35	35	26.663	35	35
205	nonstudy	24months	35	34.158	35	35	35	35	35	35	35	35	35	35	35	35	35	28.942	35	35	35
206	azithro	0months	35	35	30.56	35	35	35	35	35	35	35	22.976	35	21.96	35	35	35	28.734	35	35
207	azithro	12months	35	29.296	29.943	35	35	35	35	35	35	35	35	35	35	35	35	35	27.224	35	35
208	placebo	0months	31.467	35	26.97	35	35	35	35	30.535	35	35	20.156	28.261	25.18	35	30.724	33.58	21.481	35	35
209	nonstudy	12months	35	35	23.775	35	35	35	35	35	35	35	29.109	35	33.767	35	35	35	35	35	35
210	nonstudy	0months	35	35	35	35	35	35	35	35	35	35	20.669	20.946	23.726	35	35	35	28.465	35	35
211	placebo	24months	30.461	35	29.756	35	35	35	35	24.558	35	35	20.643	35	35	35	35	35	23.054	35	35
212	nonstudy	0months	27.024	35	35	35	35	35	35	35	35	35	21.86	27.734	22.445	35	35	35	25.658	35	35
213	azithro	12months	35	35	23.791	35	35	35	35	32.381	35	35	16.688	35	22.097	35	35	35	23.938	35	35
214	placebo	24months	35	35	35	35	35	35	35	35	35	35	35	35	35	35	35	35	35	35	35
215	nonstudy	0months	35	35	35	35	35	28.414	35	35	35	31.136	35	35	35	35	35	35	35	35	35
216	nonstudy	0months	33.501	26.681	33.082	35	35	35	35	35	31.748	35	19.242	35	21.601	35	35	35	26.105	35	35
217	azithro	0months	32.985	35	28.906	35	35	35	35	24.513	35	35	35	35	23.357	35	35	35	21.409	35	34.409
218	nonstudy	0months	35	35	35	35	21.791	35	35	35	35	33.557	25.811	35	24.91	35	35	35	26.147	35	35
219	azithro	24months	35	35	33.462	35	35	16.214	35	32.539	35	35	21.813	35	23.427	35	35	35	24.487	35	35
220	nonstudy	12months	35	35	24.932	35	35	35	35	24.305	35	35	20.332	35	19.863	35	35	35	20.568	35	35
221	nonstudy	24months	23.735	35	30.723	35	35	35	35	35	35	35	21.393	35	22.258	35	35	35	25.606	35	35
222	nonstudy	24months	35	35	35	35	35	35	35	35	35	35	20.287	35	22.992	35	35	35	22.012	35	35
223	azithro	12months	35	28.247	27.467	35	35	35	35	31.958	35	35	19.624	35	21.193	35	35	35	24.783	30.784	35
224	nonstudy	0months	35	35	25.43	35	35	35	35	35	35	35	27.658	35	20.339	35	35	35	23.023	35	35
225	placebo	24months	35	35	25.484	35	35	35	35	35	35	35	20.658	35	22.466	35	35	35	24.392	35	35
226	azithro	24months	35	35	30.682	35	35	35	35	31.658	35	34.944	20.981	35	23.183	35	35	35	21.858	35	35
227	nonstudy	0months	29.213	35	29.886	35	35	35	35	35	35	35	22.825	35	25.495	35	35	35	27.582	35	35
228	nonstudy	0months	35	35	30.96	22.714	35	35	35	35	35	35	26.938	35	16.082	35	35	31.778	18.953	35	35
229	nonstudy	24months	31.883	35	34.052	35	35	35	35	35	17.171	35	17.954	35	22.48	35	35	35	23.535	35	35
230	nonstudy	0months	35	35	35	35	35	35	35	35	35	35	27.079	35	31.151	35	35	33.906	29.135	35	35
231	placebo	24months	28.587	35	26.607	35	35	35	35	35	16.487	35	33.391	35	27.63	35	35	35	27.492	35	35
232	nonstudy	24months	35	35	25.974	35	35	35	25.104	35	35	32.876	24.743	35	35	35	35	35	26.219	35	35
233	nonstudy	0months	35	35	33.789	35	21.265	35	35	30.953	35	35	23.789	35	27.717	35	35	35	27.736	35	35
234	azithro	12months	35	35	24.966	35	35	35	35	29.836	35	35	19.563	35	22.166	35	35	35	26.037	35	33.048
235	placebo	24months	31.061	25.51	32.204	35	35	35	35	35	35	35	26.857	35	23.919	35	35	35	33.952	35	35
236	placebo	24months	30.104	35	25.588	35	35	35	35	35	35	35	22.442	35	23.01	35	30.088	35	27.771	35	35
237	placebo	0months	32.773	33.836	26.48	35	35	35	35	35	35	35	18.954	35	29.76	35	35	35	22.089	35	35
238	nonstudy	0months	31.075	35	35	35	35	35	35	35	35	35	19.198	35	22.502	35	35	35	24.437	35	35
239	azithro	12months	35	35	24.088	35	35	35	35	27.978	35	35	21.584	35	22.611	35	35	35	22.703	35	35
240	nonstudy	24months	35	34.546	29.936	35	35	15.964	35	35	35	35	23.165	35	22.372	35	35	35	26.055	35	35
241	nonstudy	0months	35	35	35	35	35	35	35	35	35	35	21.392	35	20.24	35	35	35	20.176	35	35
242	azithro	0months	35	35	26.812	35	35	35	35	33.949	35	35	23.586	35	21.542	35	35	35	25.421	35	35
243	placebo	0months	35	35	35	35	35	35	35	35	35	35	35	35	25.078	35	35	33.589	28.227	35	35
244	placebo	0months	35	35	35	35	35	35	35	35	35	35	35	35	35	35	35	35	35	35	35
245	azithro	12months	35	35	31.231	35	35	35	35	35	35	35	29.455	35	24.959	35	35	35	28.791	35	35
246	azithro	12months	35	35	30.238	35	35	35	35	30.912	33.462	35	26.663	35	21.309	35	35	35	30.352	35	35
247	placebo	24months	35	30.468	32.625	35	35	35	35	35	34.908	35	35	35	25.82	35	35	35	25.778	35	35
248	azithro	0months	35	35	25.651	35	35	35	35	27.367	35	35	35	35	23.736	35	34.893	35	26.369	35	35
249	placebo	0months	35	35	25.81	35	35	35	35	26.186	35	35	20.426	35	22.071	35	35	35	21.856	35	35
250	placebo	24months	35	35	35	35	35	35	35	35	35	35	35	35	32.624	35	35	27.018	35	35	35
251	placebo	12months	35	27.383	24.929	35	35	35	35	34.214	35	35	26.276	35	23.14	35	35	35	25.186	35	35
252	placebo	0months	35	35	22.42	35	35	35	35	33.875	35	35	17.764	35	18.88	35	35	35	19.02	35	35
253	nonstudy	12months	35	34.412	25.808	35	35	35	35	35	35	35	22.52	33.781	24.129	35	35	35	23.439	35	35
254	placebo	24months	35	35	33.522	35	35	35	35	30.4	25.951	35	35	35	16.772	35	35	26.549	21.121	35	35
255	nonstudy	24months	35	35	35	35	35	35	35	29.79	35	35	27.878	35	28.108	35	35	35	35	35	35
256	nonstudy	24months	35	35	27.59	35	35	35	35	31.964	35	33.965	21.231	35	28.576	35	35	35	25.496	35	35
257	nonstudy	24months	35	33.702	25.557	35	35	35	22.391	35	35	35	20.516	22.08	26.265	35	35	35	25.504	35	35
258	nonstudy	12months	35	35	26.588	35	35	35	35	25.739	35	35	35	35	35	35	35	35	25.047	35	35
259	placebo	0months	35	35	34.022	35	35	35	35	33.094	35	35	32.744	35	26.256	35	35	35	27.188	35	35
260	nonstudy	24months	35	34.16	29.971	35	35	35	19.326	35	31.207	35	26.45	35	21.37	35	35	35	28.79	35	35
261	nonstudy	24months	35	35	33.877	35	35	35	35	30.135	35	28.406	19.178	35	25.501	35	35	35	25.761	35	35
262	azithro	0months	32.205	35	27.647	35	35	35	35	32.013	35	35	25.998	35	21.775	35	30.462	35	17.671	35	35

263	nonstudy	24months	34.133	35	35	35	35	35	35	28.724	35	35	20.471	35	28.945	35	35	35	24.992	35	33.992
264	nonstudy	24months	35	35	35	35	35	35	19.016	35	35	35	33.616	35	35	35	35	35	29.564	35	35
265	azithro	12months	29.637	35	35	35	35	35	35	33.325	35	35	26.557	35	22.313	35	35	35	27.984	35	35
266	nonstudy	0months	35	35	28.428	35	17.101	35	32.335	35	32.149	35	23.939	35	19.357	35	35	35	24.293	35	35
267	nonstudy	0months	35	35	32.056	35	35	35	35	35	35	35	21.629	35	32.37	35	35	35	26.528	35	35
268	nonstudy	0months	35	35	35	35	30.006	35	35	29.727	35	35	19.267	35	26.338	35	35	35	23.414	35	35
269	azithro	12months	35	35	35	35	35	35	35	35	35	35	26.662	35	22.059	35	35	35	30.677	35	35
270	azithro	24months	35	35	34.936	35	35	35	35	26.777	35	35	20.358	35	26.914	35	35	35	24.33	35	35
271	azithro	24months	33.795	35	35	35	34.066	35	35	33.473	35	35	21.581	35	33.579	35	35	35	24.129	35	35
272	azithro	12months	35	35	35	35	35	35	28.185	35	35	35	19.399	35	22.461	35	35	35	21.645	35	35
273	nonstudy	0months	35	35	28.713	35	35	35	35	35	35	35	25.219	35	25.935	35	35	35	28.323	35	35
274	placebo	0months	35	28.535	31.225	35	35	35	35	35	35	35	24.638	35	30.99	35	35	35	25.794	35	35
275	azithro	12months	35	35	27.821	35	35	35	35	35	35	29.34	30.82	35	26.367	35	35	35	29.446	35	35
276	placebo	24months	35	35	27.881	35	35	35	22.678	32.48	35	35	17.955	35	26.137	35	35	35	23.118	35	35
277	nonstudy	24months	16.821	10.923	35	35	35	35	35	30.928	34.723	35	17.531	35	19.903	35	18.699	35	22.044	35	35
278	placebo	0months	35	32.418	32.024	35	35	35	35	34.417	35	35	19.187	35	22.115	35	31.837	35	23.619	35	35
279	placebo	12months	35	32.782	24.81	35	35	35	35	35	35	35	23.277	35	22.055	35	35	35	26.949	35	35
280	azithro	12months	35	35	30.117	35	35	35	35	29.825	35	35	33.728	35	23.797	35	35	35	27.456	35	35
281	placebo	24months	35	34.292	35	35	35	35	35	34.584	35	35	19.871	35	22.238	35	30.378	35	22.374	35	35
282	nonstudy	24months	35	34.022	34.533	35	35	35	35	35	35	35	30.217	35	25.538	35	35	35	22.152	35	35
283	azithro	0months	33.984	34.35	30.52	35	35	35	35	35	35	35	25.371	35	28.978	35	35	35	25.176	35	35
284	nonstudy	24months	35	35	35	35	35	35	35	35	35	35	20.096	35	35	35	35	31.881	26.021	35	35
285	azithro	12months	35	35	35	35	35	35	35	35	35	35	35	35	35	35	35	35	35	35	35
286	placebo	0months	35	35	35	35	35	35	35	28.458	35	35	21.083	35	22.543	35	35	35	23.25	35	35
287	placebo	0months	35	35	28.478	35	35	35	35	34.217	35	35	18.097	35	22.364	35	35	35	23.925	35	33.626
288	azithro	24months	35	35	35	35	35	35	35	35	33.49	35	29.541	35	35	35	35	35	29.977	35	35
289	placebo	24months	35	33.808	35	35	35	35	35	34.728	35	35	35	35	35	35	35	35	29.789	35	35
290	nonstudy	24months	35	35	27.962	35	35	35	35	30.806	35	35	17.969	35	22.397	35	35	35	23.258	35	35
291	azithro	24months	35	35	23.662	35	35	35	35	30.874	35	35	23.05	35	29.638	35	35	35	28.573	35	35
292	placebo	12months	35	32.2	30.951	35	35	35	35	35	35	33.74	20.983	35	21.485	35	35	35	23.462	35	35
293	nonstudy	0months	35	35	30.462	35	35	35	35	27.152	35	31.798	27.416	35	23.049	35	35	35	31.014	35	35
294	placebo	0months	35	35	35	35	35	19.168	35	35	35	35	23.392	35	31.408	35	35	35	26.981	35	35
295	azithro	24months	34.224	35	35	35	35	35	35	35	35	35	35	35	24.698	35	35	35	35	35	35
296	nonstudy	0months	35	35	35	35	35	35	35	35	35	35	24.78	35	35	35	35	35	27.117	35	35
297	nonstudy	24months	35	34.394	35	35	35	35	35	24.502	35	35	20.702	28.924	23.22	35	35	35	23.623	35	35
298	azithro	24months	35	35	28.475	35	35	35	35	35	21.61	35	22.849	35	25.833	35	35	35	25.778	35	35
299	placebo	24months	35	35	28.262	35	35	35	35	26.517	35	35	26.752	35	35	35	35	35	22.595	35	35
300	azithro	24months	35	35	31.721	35	35	35	31.075	27.49	33.158	35	25.235	35	32.433	35	35	35	29.72	35	35
301	nonstudy	24months	35	35	32.918	35	35	35	35	35	35	35	30.67	35	35	35	35	35	27.259	35	35
302	azithro	0months	35	35	30.942	35	35	35	20.502	35	35	35	31.134	35	22.341	35	35	33.479	21.906	35	35
303	nonstudy	0months	35	35	29.367	35	35	13.838	35	35	35	35	23.943	35	21.798	35	35	35	25.05	35	35
304	placebo	12months	35	35	35	35	35	33.735	35	34.267	35	35	22.511	35	20.41	35	35	35	29.552	35	35
305	azithro	24months	23.628	35	35	35	35	34.085	35	35	35	35	19.264	35	35	35	35	35	35	35	35
306	azithro	0months	35	35	35	35	35	35	27.067	35	35	35	17.849	35	20.81	35	35	35	24.742	35	35
307	placebo	0months	35	35	35	35	35	35	35	35	35	35	19.207	35	23.464	35	35	35	29.839	35	35
308	azithro	24months	35	35	22.489	35	35	35	35	26.006	35	35	23.478	35	28.101	35	35	35	28.297	35	35
309	nonstudy	24months	35	35	31.714	35	35	33.906	35	28.356	35	35	33.468	35	35	35	33.714	35	24.633	35	35
310	azithro	24months	35	35	24.923	35	35	35	35	35	35	34.992	17.017	35	21.317	35	23.412	35	21.444	30.479	35
311	placebo	12months	35	35	28.758	35	35	35	35	35	35	33.893	26.277	35	20.012	35	35	35	23.805	34.238	35
312	placebo	12months	35	35	35	35	35	35	35	35	35	35	21.142	35	22.451	35	35	35	24.468	35	35
313	nonstudy	0months	35	35	22.593	35	31.271	35	35	35	35	35	20.875	35	21.279	35	35	35	22.915	35	35
314	placebo	12months	35	35	35	35	35	35	35	35	35	34.575	30.358	35	23.232	35	35	35	26.999	35	35
315	azithro	0months	35	35	23.126	35	35	35	35	26.417	35	35	20.958	35	22.649	35	34.355	35	22.813	35	35
316	nonstudy	0months	35	35	34.52	35	26.304	35	35	35	35	35	21.684	35	24.234	35	35	35	21.068	35	35
317	placebo	24months	35	35	35	35	35	35	35	35	35	35	23.978	35	31.264	35	35	35	35	35	35
318	nonstudy	24months	33.182	35	35	35	35	35	35	35	35	35	20.595	35	35	35	35	35	30.981	35	35
319	nonstudy	0months	35	35	35	35	35	35	35	35	35	35	19.969	19.996	22.071	35	35	35	27.305	35	35
320	nonstudy	24months	35	35	35	35	35	35	33.808	35	35	35	35	35	26.2	35	35	35	23.607	35	35
321	nonstudy	24months	35	35	35	35	35	35	35	35	35	35	35	35	35	35	35	29.415	35	35	35
322	azithro	12months	27.961	32.374	35	35	35	35	27.822	32.913	35	35	22.733	22.58	20.36	35	35	35	25.527	35	35
323	nonstudy	12months	35	35	35	35	35	35	35	34.275	35	35	27.92	35	25.18	35	35	35	30.038	35	35
324	nonstudy	12months	35	35	33.966	35	35	35	35	28.147	35	35	35	35	35	35	35	35	21.35	35	35
325	nonstudy	12months	27.319	35	32.542	35	35	35	35	32.09	35	35	21.629	35	21.254	35	35	35	29.143	35	35
326	placebo	0months	35	35	34.576	35	35	35	35	35	35	35	25.468	35	19.913	35	35	35	25.075	35	35
327	nonstudy	0months	32.942	35	35	35	35	35	35	35	35	35	35	35	27.872	35	35	35	35	35	35
328	azithro	0months	34.071	33.527	35	35	35	35	35	27.855	35	35	20.281	35	19.339	35	35	35	24.106	35	35

329	nonstudy	12months	35	35	30.341	35	35	35	35	27.328	35	35	25.665	35	26.065	35	35	35	24.051	35	35
330	azithro	24months	35	35	33.19	35	35	35	35	22.432	35	35	35	35	27.418	35	35	35	27.896	35	35
331	nonstudy	0months	35	35	32.506	35	35	35	18.631	35	35	35	22.891	35	22.687	35	18.099	35	29.887	35	35
332	azithro	12months	35	35	33.167	35	35	35	35	35	35	35	28.819	35	21.495	35	35	35	26.773	35	35
333	azithro	0months	30.19	35	32.683	35	35	35	35	35	35	35	21.708	35	24.647	35	31.229	35	23.317	33.016	35
334	nonstudy	12months	35	34.366	23.146	35	35	35	35	35	35	35	33.337	35	35	35	35	35	35	35	35
335	nonstudy	24months	35	35	23.622	35	35	35	35	35	35	35	19.898	35	20.505	35	35	35	28.284	35	35
336	nonstudy	12months	35	35	31.131	35	35	35	33.722	22.869	35	35	22.329	35	35	35	35	35	22.069	35	35
337	placebo	24months	35	35	33.138	35	35	35	35	33.842	35	35	29.958	35	22.306	35	35	34.352	22.379	35	35
338	nonstudy	24months	33.393	35	35	35	35	35	35	21.781	35	34.614	18.679	35	22.019	35	35	35	25.713	35	35
339	nonstudy	24months	35	35	28.553	35	35	35	35	35	34.585	35	21.101	35	19.562	35	33.59	35	23.325	35	35
340	azithro	24months	35	35	35	35	35	35	35	25.227	35	35	26.75	35	23.072	35	35	35	35	35	35
341	placebo	12months	35	35	27.593	35	35	35	28.491	34.884	35	35	21.126	35	22.384	35	35	35	24.616	35	35
342	azithro	24months	35	35	25.596	35	35	35	35	24.998	35	35	19.804	35	35	35	35	35	23.433	35	32.297
343	azithro	0months	35	35	29.399	35	35	35	35	33.747	35	35	25.391	35	24.958	35	35	35	24.196	35	35
344	placebo	24months	35	35	29.949	35	35	35	35	35	35	35	20.708	35	21.526	35	35	35	25.009	35	35
345	nonstudy	24months	35	35	35	35	35	27.258	35	35	35	35	27.982	35	23.111	35	30.509	35	24.225	35	35
346	azithro	0months	35	35	35	35	35	35	35	30.986	35	35	19.546	35	19.877	35	35	35	22.731	35	35
347	azithro	0months	35	35	35	35	35	32.234	35	35	35	32.333	35	35	35	35	35	35	35	35	35
348	nonstudy	0months	35	35	28.941	35	17.891	35	35	35	35	35	24.568	35	24.03	35	35	35	27.528	35	35
349	placebo	24months	35	28.966	31.081	35	35	35	35	21.834	35	35	19.781	35	21.356	35	31.854	35	27.625	35	35
350	azithro	24months	35	35	35	35	35	35	35	23.835	35	35	24.904	35	27.601	35	35	35	24.484	35	35
351	placebo	12months	35	35	33.206	35	35	35	35	35	35	35	23.812	35	23.796	35	35	35	35	35	35
352	azithro	12months	30.989	35	27.742	35	35	35	35	25.052	35	35	19.94	35	23.546	35	35	35	24.26	35	35
353	placebo	0months	35	35	30.069	35	35	35	35	32.533	35	35	33.297	35	26.661	35	35	35	34.041	35	35
354	placebo	12months	35	35	35	23.713	35	35	35	29.239	35	32.168	18.971	35	28.217	35	35	35	33.925	35	35
355	nonstudy	24months	35	35	35	35	35	26.858	35	35	34.66	35	21.219	35	22.469	35	35	34.237	23.25	33.665	35
356	nonstudy	12months	35	29.142	27.603	35	35	35	35	35	35	35	24.702	35	27.311	35	35	35	26.755	35	35
357	nonstudy	24months	35	35	25.685	35	35	35	35	25.367	35	35	19.371	35	24.306	35	35	35	24.35	35	35
358	placebo	24months	35	35	25.275	35	35	35	23.27	35	35	35	18.966	35	22.276	35	35	35	29.678	35	35
359	nonstudy	0months	35	35	28.415	35	35	35	35	28.074	35	35	24.031	35	23.296	35	35	35	23.288	35	35
360	nonstudy	0months	35	35	20.665	35	35	35	35	32.991	35	35	16.139	35	21.091	35	35	35	21.49	35	35
361	azithro	24months	31.575	35	31.049	35	35	35	35	24.114	35	35	21.731	35	23.195	35	22.467	35	21.487	35	35
362	azithro	0months	35	35	35	35	35	35	35	31.792	35	35	23.609	35	35	35	35	35	22.535	35	35
363	azithro	0months	35	35	24.821	35	35	35	35	35	35	35	21.578	35	24.813	35	35	35	25.124	35	35
364	azithro	24months	35	35	26.842	35	35	35	35	26.305	35	35	24.931	35	26.147	35	35	35	24.267	35	35
365	azithro	24months	35	35	31.18	35	35	35	35	27.513	35	35	23.283	35	22.743	35	35	35	26.956	35	35
366	nonstudy	0months	14.174	35	31.316	35	35	35	35	35	35	35	19.56	35	22.309	35	35	35	29.92	35	35
367	nonstudy	0months	35	33.967	30.633	35	35	35	35	31.154	35	35	19.796	35	18.175	35	20.971	35	22.89	35	35
368	placebo	0months	35	35	24.77	35	35	35	35	35	35	35	18.681	35	20.684	35	35	35	22.801	35	35
369	nonstudy	12months	35	35	35	35	35	35	35	30.992	35	33.885	26.852	35	23.631	35	35	35	29.57	35	35
370	placebo	0months	26.429	16.397	35	35	35	35	35	27.31	35	32.577	19.609	35	26.649	35	35	35	25.932	35	35
371	placebo	12months	35	35	35	35	35	35	35	28.368	35	35	32.966	35	35	35	35	35	35	35	35
372	nonstudy	0months	30.763	35	26.505	35	35	35	35	29.675	35	35	18.447	35	25.184	35	35	35	17.771	35	35
373	nonstudy	24months	35	35	26.19	35	35	35	35	28.001	35	35	22.341	35	23.134	35	35	32.376	23.117	35	34.517
374	azithro	0months	35	35	20.793	35	35	35	35	35	35	35	20.17	35	25.457	35	30.696	35	29.514	35	35
375	nonstudy	0months	35	35	27.047	35	35	35	21.755	32.888	35	35	17.542	35	21.478	35	35	31.509	22.608	35	35
376	nonstudy	0months	35	31.577	27.555	35	35	35	35	35	35	35	19.695	35	18.644	35	35	35	22.714	35	35
377	placebo	0months	35	35	35	35	35	35	35	35	35	32.872	18.884	19.282	20.9	35	35	35	25.612	35	35
378	placebo	24months	35	35	35	35	35	35	35	35	35	35	20.072	19.946	33.848	35	35	35	23.698	35	35
379	placebo	0months	35	35	28.931	35	35	35	35	35	35	35	23.463	35	21.371	35	35	30.638	23.279	35	35
380	azithro	24months	35	35	28.249	35	35	35	31.623	35	35	35	21.455	35	25.135	35	35	35	29.195	35	35
381	azithro	12months	35	35	32.465	35	35	35	35	25.59	35	35	20.967	35	21.639	35	34.396	35	23.47	35	35
382	placebo	24months	35	35	28.347	35	35	35	35	26.439	35	33.712	22.547	21.073	34.481	35	35	35	25.346	35	35
383	azithro	24months	35	35	21.899	35	35	35	35	35	35	35	21.227	35	28.832	35	35	35	30.799	35	35
384	azithro	12months	35	35	35	35	35	35	35	35	35	35	20.702	35	22.538	35	35	35	24.221	35	35
385	nonstudy	24months	35	35	31.558	35	35	35	35	35	35	35	27.479	35	22.551	35	35	35	24.59	35	35
386	nonstudy	12months	35	35	28.372	27.663	35	35	35	35	35	35	35	35	28.535	35	35	35	35	35	35
387	placebo	12months	35	35	34.573	35	35	35	35	29.527	35	35	20.34	35	23.05	35	35	33.627	23.055	35	35
388	placebo	12months	35	35	30.562	35	35	35	35	34.042	35	35	21.267	33.321	21.247	35	35	33.413	24.361	35	35
389	nonstudy	0months	35	34.764	28.603	35	20.89	35	35	35	33.512	35	23.236	35	33.763	35	35	35	25.788	35	35
390	nonstudy	12months	35	32.029	35	35	35	35	35	35	35	35	35	35	23.127	35	35	35	25.545	35	35
391	nonstudy	24months	35	35	35	35	35	35	35	22.32	35	35	21.994	35	22.783	35	35	35	27.469	35	35
392	nonstudy	0months	35	12.956	27.122	35	35	35	23.421	35	35	35	18.125	35	30.865	35	35	35	20.832	35	35
393	nonstudy	0months	35	33.261	35	35	35	33.768	35	35	35	31.318	35	35	35	35	35	35	35	35	35
394	azithro	12months	35	34.622	33.137	35	35	35	35	35	35	35	31.582	35	22.236	35	35	35	27.545	35	35

395	placebo	0months	28.611	33.422	30.071	35	35	35	21.975	35	34.734	35	31.78	35	23.754	35	35	35	27.028	35	35
396	nonstudy	0months	35	34.384	33.51	35	35	35	35	35	35	35	24.658	35	33.252	35	35	35	34.974	35	35
397	azithro	12months	35	35	35	35	35	35	35	35	35	35	27.552	35	35	35	35	35	25.1	35	35
398	placebo	0months	27.993	35	35	35	20.132	35	35	35	35	35	22.486	35	20.497	35	35	35	21.983	35	35
399	nonstudy	0months	35	35	31.632	20.337	35	35	35	35	35	35	21.937	35	25.209	35	35	35	35	35	35
400	nonstudy	12months	28.589	26.951	28.079	35	35	35	34.048	32.755	35	35	21.738	35	21.065	35	35	35	20.969	35	35
401	placebo	0months	35	35	35	35	35	35	35	27.263	35	27.916	20.658	35	25.81	35	35	35	24.796	35	35
402	nonstudy	0months	35	35	31.544	35	35	35	35	27.139	35	35	19.377	35	25.589	35	35	35	25.857	35	35
403	nonstudy	0months	35	35	25.249	35	35	35	35	35	35	35	21.633	35	19.651	35	35	35	25.975	35	35
404	nonstudy	24months	27.319	35	35	35	35	35	35	35	35	33.9	19.657	35	26.97	35	35	35	24.92	35	35
405	nonstudy	24months	35	35	35	35	35	35	35	35	35	35	29.818	35	22.413	35	35	33.443	28.361	35	35
406	azithro	24months	35	35	30.316	35	35	35	35	27.493	35	35	23.272	35	23.586	35	35	35	23.952	35	35
407	placebo	12months	35	35	32.737	35	35	35	35	31.825	35	35	20.553	35	22.532	35	35	35	25.917	35	35
408	nonstudy	0months	35	34.492	35	35	35	35	32.993	35	35	35	22.071	35	21.09	35	33.023	35	24.93	35	35
409	placebo	24months	30.389	35	22.291	35	35	35	35	35	35	35	29.002	35	35	35	35	35	29.124	35	35
410	azithro	12months	35	35	31.291	35	35	35	35	28.96	35	35	23.313	35	21.318	35	35	35	26.041	35	35
411	azithro	24months	31.65	34.607	26.108	35	35	35	35	32.759	35	35	24.478	35	19.711	35	35	35	28.069	35	35
412	azithro	24months	35	35	29.831	35	35	35	35	35	35	35	32.04	35	35	35	35	35	25.494	35	35
413	placebo	24months	30.248	35	30.599	35	35	35	35	31.014	35	35	19.027	35	20.651	35	35	33.604	24.951	35	35
414	azithro	0months	33.594	35	35	35	35	35	35	29.484	35	35	19.644	35	24.577	35	35	35	27.743	35	35
415	azithro	24months	35	35	35	35	35	35	35	35	35	35	35	35	35	35	35	29.015	35	35	35
416	placebo	0months	35	35	35	35	35	35	35	35	35	35	19.981	19.891	22.902	35	35	35	28.499	35	35
417	nonstudy	0months	24.905	35	33.181	NA	35	35	35	35	35	35	21.796	35	19.662	35	35	35	24.464	35	35
418	nonstudy	0months	35	35	32.339	35	35	35	35	29.248	35	35	22.876	35	24.097	35	35	35	23.147	35	35
419	azithro	24months	35	35	35	35	35	35	18.867	35	35	35	35	35	25.149	35	35	31.327	26.968	35	35
420	placebo	0months	35	35	35	35	35	35	35	35	35	35	19.929	35	21.688	35	35	35	24.476	35	35
421	placebo	12months	32.109	35	29.095	35	35	35	35	26.821	35	35	20.811	35	23.036	35	35	35	26.959	35	35
422	azithro	24months	34.327	30.207	23.675	35	35	35	35	26.727	35	30.557	20.134	35	22.331	35	35	35	31.423	35	35
423	nonstudy	0months	31.515	35	35	35	35	35	35	35	32.758	35	17.73	35	21.268	35	19.045	35	21.071	35	35
424	azithro	24months	21.386	35	28.49	35	35	35	35	35	35	35	17.481	35	23.687	35	35	35	35	35	35
425	azithro	0months	32.829	34.497	26.415	35	35	35	35	34.631	34.203	35	18.692	35	19.532	35	35	35	22.532	35	35
426	placebo	0months	28.093	35	28.443	35	35	35	29.759	35	35	35	24.669	34.849	22.349	35	35	35	27.844	35	35
427	azithro	12months	35	35	27.455	35	35	35	35	33.896	35	35	23.023	35	35	35	35	35	29.91	35	35
428	azithro	24months	31.27	35	24.204	35	35	35	35	35	35	32.5	25.153	35	23.02	35	35	35	27.126	35	35
429	nonstudy	12months	35	35	33.15	35	35	35	35	35	35	35	35	35	24.903	35	35	35	35	35	35
430	nonstudy	24months	35	35	34.98	35	35	35	27.476	34.088	35	35	20.287	35	29.345	35	35	35	24.956	35	35
431	nonstudy	24months	35	35	30.736	35	35	24.97	35	35	35	35	18.932	35	22.779	35	35	35	25.693	35	35
432	azithro	0months	35	35	35	35	35	35	35	35	35	35	19.343	35	35	35	35	35	34.049	35	35
433	azithro	0months	16.482	35	30.446	35	35	35	35	35	35	35	31.093	35	24.399	35	35	35	27.179	35	35
434	azithro	0months	35	35	20.282	35	35	35	33.583	30.943	35	35	23.658	35	22.627	35	35	35	23.136	35	35
435	azithro	24months	35	35	34.575	35	35	35	35	35	28.233	35	22.125	35	23.898	35	35	35	25.048	35	32.548
436	azithro	24months	35	29.774	24.867	35	35	35	35	28.033	31.931	35	26.251	35	22.636	35	35	34.708	24.96	34.031	35
437	nonstudy	0months	31.101	35	27.988	35	35	35	35	35	35	35	20.213	35	23.518	35	35	35	24.471	35	35
438	azithro	12months	35	35	34.289	35	35	35	35	31.615	35	35	24.691	35	22.069	35	35	35	26.986	35	35
439	azithro	0months	35	35	29.58	35	24.588	35	35	35	35	35	20.661	35	22.795	35	35	35	28.902	35	35
440	azithro	0months	35	35	28.433	35	35	35	35	32.804	35	30.286	17.418	35	35	35	35	33.993	22.287	35	35
441	nonstudy	24months	35	35	31.431	35	35	35	35	35	35	35	21.768	35	20.658	35	35	35	24.255	35	35
442	placebo	12months	35	35	29.281	35	35	35	35	26.117	35	33.848	24.03	35	35	35	35	35	35	35	35
443	placebo	0months	35	35	31.58	35	35	35	35	35	35	31.316	35	35	27.722	35	35	35	29.207	35	35
444	azithro	24months	35	35	35	35	35	35	35	31.968	35	35	28.436	35	27.911	35	35	35	25.673	35	35
445	nonstudy	24months	35	35	35	35	35	35	35	25.561	35	35	26.827	35	30.057	35	35	35	25.765	35	35
446	placebo	24months	23.63	35	27.374	35	35	35	35	35	35	35	21.043	35	22.266	35	35	35	24.563	35	35
447	azithro	12months	35	35	31.748	35	35	35	35	35	35	35	19.271	35	35	35	35	27.975	35	35	35
448	nonstudy	0months	35	35	35	35	35	35	35	35	35	35	24.967	35	21.51	35	35	32.131	29.068	35	35
449	nonstudy	0months	35	35	34.234	35	35	35	35	35	35	35	25.789	35	22.021	35	35	34.959	26.803	35	35
450	placebo	0months	29.276	35	29.242	35	35	35	35	31.124	35	35	21.004	35	23.504	35	34.609	35	21.132	35	35
451	placebo	24months	35	35	35	35	35	35	35	35	23.512	35	26.42	35	35	35	35	35	25.338	35	35
452	azithro	0months	35	35	34.625	35	35	35	35	35	35	35	35	35	35	35	35	35	30.155	35	35
453	azithro	12months	35	35	32.141	35	35	35	35	30.391	35	35	19.044	35	22.837	35	35	35	23.265	35	35
454	nonstudy	24months	35	35	35	35	35	35	35	35	35	35	23.167	35	21.586	35	34.977	35	27.732	35	35
455	placebo	0months	35	34.396	29.433	35	35	35	35	35	35	35	26.35	35	22.845	35	35	35	25.036	35	35
456	nonstudy	0months	26.215	35	28.526	35	35	35	35	35	35	35	26.839	35	20.072	35	35	35	24.754	35	35
457	placebo	24months	35	35	24.459	35	35	35	35	30.556	35	35	23.455	35	26.241	35	35	35	25.569	35	35
458	placebo	0months	35	35	35	35	35	35	35	35	35	26.668	23.592	35	22.848	35	35	35	27.22	35	35
459	azithro	0months	35	35	35	35	35	35	35	35	35	35	35	35	35	35	35	35	35	35	35
460	azithro	0months	35	35	24.146	35	35	35	35	35	35	35	26.509	35	25.502	35	35	35	22.238	35	35

461	azithro	24months	32.92	28.004	23.949	35	35	35	35	31.511	35	35	23.053	35	19.805	35	35	35	20.6	35	35
462	placebo	12months	35	35	30.248	35	35	35	35	32.059	35	35	20.941	35	19.813	35	35	35	23.304	35	35
463	azithro	12months	30.081	27.663	35	35	35	35	35	35	35	35	22.77	35	19.313	35	35	35	27.713	35	35
464	azithro	0months	35	35	35	35	35	35	35	35	35	35	35	35	35	35	35	35	31.724	35	35
465	azithro	24months	31.878	35	26.494	35	35	35	33.846	27.128	35	35	20.896	35	22.223	35	35	35	22.211	35	35
466	nonstudy	24months	35	19.025	35	35	35	35	35	35	35	35	35	35	20.681	35	35	35	28.084	35	35
467	azithro	24months	32.701	35	30.031	35	35	35	35	35	35	35	35	35	35	35	35	26.918	35	35	35
468	nonstudy	0months	35	35	35	35	35	35	35	25.751	35	35	35	35	35	35	35	35	24.062	35	35
469	nonstudy	0months	35	35	35	35	35	35	35	35	35	35	23.894	35	25.515	35	35	35	27.629	35	35
470	nonstudy	24months	34.158	35	24.96	35	35	35	35	35	15.631	35	21.522	35	23.375	35	35	35	21.477	35	35
471	placebo	12months	35	35	35	35	35	35	35	35	35	35	28.726	35	22.604	35	35	35	26.694	35	35
472	placebo	24months	35	35	35	35	35	35	35	26.015	34.438	35	26.554	35	22.678	35	35	35	27.142	35	35
473	placebo	24months	35	35	32.286	35	35	35	34.628	25.985	35	35	26.852	35	35	35	35	35	28.464	35	35
474	azithro	24months	35	35	35	35	35	35	35	35	35	35	20.648	35	26.859	35	35	35	23.774	35	35
475	azithro	0months	35	35	35	35	35	35	35	30.916	26.775	35	20.522	35	27.045	35	35	35	27.133	35	35
476	nonstudy	0months	35	35	30.609	35	35	35	35	35	35	26.952	24.118	35	18.638	35	35	35	21.681	33.789	35
477	azithro	24months	35	34.418	31.888	35	35	35	35	35	34.575	35	22.533	35	24.548	35	35	35	27.337	35	35
478	nonstudy	0months	35	35	35	35	35	35	34.809	32.449	35	35	27.478	35	24.429	35	35	35	25.787	35	35
479	nonstudy	12months	35	35	33.918	35	35	35	35	35	35	35	35	35	35	35	35	33.743	35	35	35
480	azithro	0months	35	35	35	35	35	35	35	35	35	35	18.75	35	20.583	35	35	35	19.78	35	35
481	nonstudy	24months	35	35	28.824	35	35	35	35	25.037	34.503	35	24.519	35	24.26	35	35	35	26.748	35	35
482	azithro	0months	35	35	35	35	35	29.504	35	35	35	30.257	35	35	35	35	35	35	34.146	35	35
483	nonstudy	0months	24.602	35	35	35	35	35	35	26.591	34.523	35	17.841	35	31.029	35	35	35	24.481	35	35
484	placebo	12months	35	35	29.174	35	35	35	35	32.051	35	35	20.043	35	22.967	35	34.957	35	24.717	35	35
485	placebo	0months	35	35	35	35	35	35	35	35	35	35	19.667	19.185	22.344	35	35	35	27.445	35	35
486	placebo	12months	35	35	35	35	35	35	35	35	16.881	35	24.998	35	24.478	35	35	35	25.207	35	35
487	azithro	0months	35	34.388	27.437	35	35	35	35	27.144	35	35	17.827	35	20.039	35	35	33.793	20.588	35	35
488	placebo	24months	35	35	26.914	35	35	35	20.826	32.373	35	35	22.069	24.824	22.694	35	35	35	27.457	35	35
489	azithro	12months	35	29.393	35	35	35	35	34.607	31.031	34.231	35	25.029	35	35	35	35	35	26.933	35	35
490	nonstudy	24months	35	35	29.24	35	35	35	35	31.632	35	35	24.579	35	21.881	35	35	35	26.483	32.265	35
491	azithro	12months	35	35	33.406	35	35	35	35	35	35	35	33.391	35	24.469	35	35	35	25.779	35	35
492	placebo	24months	35	35	35	35	35	35	34.796	35	22.516	35	22.104	35	24.066	35	35	33.523	31.04	35	35
493	azithro	12months	35	35	35	35	35	35	35	35	35	35	27.73	35	29.386	35	35	35	28.481	35	35
494	azithro	0months	31.469	35	26.585	35	35	35	35	23.863	35	35	35	35	21.384	35	35	35	22.225	35	35
495	azithro	0months	35	33.428	29	35	35	35	35	35	35	35	35	35	21.652	35	35	35	25.535	35	35
496	nonstudy	0months	35	34.23	24.251	35	35	35	35	35	35	35	32.719	35	22.008	35	31.772	35	26.234	33.997	35
497	placebo	0months	31.997	35	35	35	35	35	35	25.734	35	35	22.436	35	19.982	35	35	35	24.719	35	35
498	nonstudy	0months	19.993	35	31.877	35	35	35	35	34.616	35	35	18.468	35	21.43	35	35	35	22.566	35	35
499	azithro	0months	35	35	32.432	35	35	35	23.056	35	35	35	22.766	35	23.855	35	35	35	23.938	35	35
500	nonstudy	24months	35	35	35	35	35	35	35	35	16.323	35	34.147	35	22.453	35	35	35	22.898	35	35
501	nonstudy	12months	35	35	32.862	35	35	35	35	35	35	35	22.567	35	22.152	35	35	35	25.297	35	35
502	placebo	24months	35	35	28.271	35	35	35	35	31.071	35	19.738	22.578	35	21.397	35	35	35	28.889	35	35
503	placebo	12months	35	35	32.923	35	35	35	26.302	35	35	35	33.842	35	21.706	35	35	35	25.76	35	35
504	placebo	12months	28.39	35	24.971	35	35	35	35	28.954	35	35	19.451	35	19.743	35	35	35	23.224	35	35
505	nonstudy	0months	35	35	34.684	35	35	35	35	32.894	35	32.828	35	34.127	24.137	35	35	35	26.855	35	35
506	azithro	12months	35	35	35	35	35	35	35	29.425	35	35	34.489	35	35	35	35	35	29.266	35	35
507	nonstudy	12months	35	35	27.773	35	35	35	29.277	35	35	35	19.129	35	24.013	35	35	35	25.647	35	35
508	placebo	12months	35	35	35	35	35	35	35	35	35	35	26.024	35	22.086	35	35	35	26.252	35	35
509	azithro	12months	35	35	35	35	35	35	35	27.086	35	35	24.312	35	20.887	35	35	35	27.329	35	35
510	placebo	24months	35	35	35	35	35	35	35	35	35	35	23.163	35	22.375	35	35	35	26.246	35	35
511	nonstudy	24months	33.999	35	24.439	35	35	35	35	35	35	35	24.314	35	35	35	35	35	25.645	35	35
512	placebo	0months	27.568	35	22.303	35	35	35	35	27.373	35	35	27.717	35	20.246	35	35	35	26.633	35	35
513	nonstudy	24months	35	35	32.856	35	35	35	35	30.333	35	35	23.532	35	35	35	25.016	35	25.429	35	35
514	nonstudy	24months	35	35	26.593	35	35	35	35	35	35	31.931	34.752	35	28.409	35	31.292	35	25.771	35	35
515	azithro	24months	35	35	35	35	35	35	35	30.539	35	35	35	35	35	35	35	35	35	35	35
516	azithro	12months	35	35	26.575	35	35	35	35	29.644	35	35	21.701	35	17.981	35	35	35	22.597	35	35
517	placebo	0months	35	35	35	35	19.687	35	35	35	35	35	16.459	35	23.85	35	35	25.287	35	35	35
518	nonstudy	0months	35	30.728	35	34.419	35	35	35	26.281	35	35	21.941	35	27.419	35	35	35	23.64	35	35
519	nonstudy	24months	31.31	28.876	35	35	35	35	19.723	35	35	35	24.017	35	22.012	35	32.89	35	26.491	35	35
520	nonstudy	24months	32.625	35	35	35	35	35	35	29.13	32.114	35	20.442	35	23.851	35	35	35	23.293	35	35
521	azithro	0months	35	35	35	35	35	35	21.408	35	35	35	32.927	35	23.98	35	35	35	26.701	35	35
522	placebo	0months	35	35	35	35	35	35	35	35	35	35	20.14	20.589	23.913	35	35	35	29.336	35	35
523	nonstudy	12months	35	35	35	35	35	35	35	32.255	35	35	21.3	35	21.296	35	35	35	25.315	35	35
524	azithro	24months	35	18.339	35	35	35	35	24.206	35	35	35	24.294	35	21.987	35	35	35	23.652	35	35
525	placebo	12months	35	35	28.891	20.547	35	35	35	35	35	35	35	35	25.704	35	35	35	35	35	35
526	placebo	12months	35	15.412	29.578	35	35	35	35	34.445	35	35	21.577	35	18.177	35	35	35	21.75	35	35

527	placebo	12months	27.271	35	23.435	35	35	35	35	33.35	35	35	21.004	35	19.599	35	35	35	26.108	35	35
528	placebo	12months	33.514	32.995	25.727	35	35	35	35	32.005	35	35	26.977	30.124	21.334	35	35	35	29.003	35	35
529	placebo	0months	35	35	35	35	35	35	35	35	35	35	18.965	18.825	21.794	35	35	35	26.617	35	35
530	nonstudy	24months	28.256	35	26.734	35	35	35	35	31.198	35	33.216	21.922	35	22.025	35	23.61	35	24.201	35	35
531	azithro	24months	35	35	35	35	35	35	35	35	18.073	35	24.259	35	25.001	35	34.911	35	31.683	35	35
532	azithro	0months	30.926	35	26.979	35	35	35	35	31.907	35	35	23.919	35	35	35	35	27.15	24.097	35	35
533	azithro	12months	35	35	26.631	35	35	35	33.436	35	35	35	22.977	35	32.604	35	35	35	21.185	35	35
534	azithro	24months	35	35	25.756	35	35	35	35	31.729	18.039	35	30.662	35	28.48	35	35	35	30.654	35	35
535	nonstudy	12months	33.264	35	26.617	35	35	35	35	35	35	35	19.902	35	20.613	35	35	35	23.007	35	35
536	nonstudy	24months	35	31.024	35	35	35	35	26.499	35	35	35	20.111	35	21.38	35	35	35	24.253	35	35
537	azithro	12months	32.82	35	29.641	35	35	35	35	35	33.618	35	22.076	35	25.341	35	35	35	27.497	35	35
538	nonstudy	0months	33.834	34.516	31.582	35	35	35	35	35	35	35	24.565	35	22.042	35	35	35	26.713	32.404	35
539	nonstudy	12months	35	35	26.565	35	35	35	35	30.373	35	35	24.648	35	24.274	35	35	35	25.743	32.045	35
540	placebo	12months	35	35	35	35	35	35	35	35	35	35	24.88	35	35	35	35	35	35	35	35
541	placebo	24months	35	35	29.306	35	35	35	35	35	35	35	21.095	35	20.919	35	29.832	35	24.456	35	35
542	placebo	12months	35	35	28.538	35	35	35	33.278	30.129	35	35	21.957	35	24.431	35	35	35	23.235	35	35
543	nonstudy	24months	30.172	35	33.026	35	35	35	35	31.435	17.571	35	18.863	35	21.492	35	35	35	23.384	35	35
544	azithro	0months	35	35	35	35	35	35	35	35	35	34.751	17.976	35	22.032	35	35	35	21.176	35	35
545	placebo	24months	27.958	35	28.686	35	35	35	35	35	35	35	19.71	35	25.035	35	26.859	35	24.833	35	35
546	placebo	0months	32.032	35	33.969	35	35	35	35	27.064	35	35	30.234	35	22.807	35	35	35	26.954	35	35
547	azithro	24months	35	35	35	35	35	35	35	35	35	35	35	35	35	35	28.669	28.092	35	35	35
548	placebo	0months	35	35	35	35	15.764	35	35	35	35	35	30.165	35	21.452	35	35	35	32.757	35	35
549	nonstudy	12months	35	31.852	26.641	35	35	35	35	35	35	35	23.99	35	22.503	35	35	35	24.214	35	35
550	placebo	0months	35	35	35	35	35	35	35	35	35	35	25.608	35	22.15	35	35	35	26.625	35	35
551	nonstudy	12months	35	35	35	35	35	35	35	35	35	35	30.214	35	27.221	35	35	35	35	35	35
552	nonstudy	0months	35	35	28.263	35	35	35	35	35	35	35	30.801	35	33.415	35	35	35	27.195	35	35
553	placebo	0months	24.042	35	35	35	35	35	35	27.955	35	24.435	21.91	35	24.609	35	35	35	26.724	35	35
554	nonstudy	0months	33.725	35	27.567	35	35	35	35	24.994	35	35	24.978	35	23.893	35	35	35	28.289	35	35
555	nonstudy	24months	35	35	31.629	35	35	35	35	35	35	35	24.006	35	35	35	35	34.539	35	35	35
556	placebo	0months	35	35	28.044	35	35	35	35	35	35	35	21.341	35	24.518	35	35	35	25.046	35	35
557	nonstudy	0months	35	35	30.602	35	35	35	35	29.64	35	34.203	27.981	35	35	35	35	35	35	35	35
558	placebo	24months	35	35	28.576	35	35	35	35	30.899	35	35	23.349	35	21.955	35	35	35	25.03	35	35
559	azithro	24months	35	31.482	22.311	35	35	35	35	35	35	35	24.972	35	26.21	35	35	35	26.083	35	35
560	nonstudy	24months	35	33.192	29.108	35	35	20.371	35	35	35	35	19.68	35	22.81	35	35	35	26.591	35	35
561	azithro	0months	35	29.397	24.522	35	35	35	35	29.888	35	35	15.624	35	18.745	35	35	35	21.448	35	35
562	nonstudy	0months	35	35	26.238	35	35	35	35	35	35	35	19.084	35	20.766	35	35	35	22.778	35	35
563	nonstudy	24months	35	35	25.39	35	35	35	35	35	35	35	21.532	35	21.803	35	35	35	23.838	35	35
564	nonstudy	12months	35	35	35	35	35	35	35	35	35	35	20.917	35	26.691	35	35	35	23.784	35	35
565	nonstudy	24months	35	35	29.715	35	35	35	35	26.819	35	35	25.987	35	23.073	35	35	35	26.559	35	35
566	azithro	0months	35	35	27.468	35	26.677	35	35	35	35	35	20.27	35	22.984	35	35	35	23.876	35	35
567	azithro	0months	35	35	35	34.17	35	35	35	31.479	35	34.679	22.934	35	26.795	35	35	35	25.973	35	35
568	nonstudy	24months	35	35	35	35	35	35	35	35	35	35	19.168	19.684	25.524	35	32.773	35	23.536	35	35
569	azithro	24months	35	35	35	35	35	35	35	28.983	35	35	21.691	35	35	35	35	35	24.899	35	35
570	placebo	0months	35	18.67	25.665	35	35	35	31.528	35	35	35	28.92	35	21.453	35	35	35	24.71	35	35
571	placebo	12months	35	35	26.848	35	35	35	35	27.348	35	35	21.598	35	21.671	35	35	35	24.073	35	35
572	placebo	12months	35	31.126	27.453	35	35	35	35	25.855	35	35	21.994	35	25.551	35	35	35	28.412	35	35
573	azithro	0months	35	35	29.15	35	35	35	35	35	35	35	35	35	35	35	35	35	35	35	35
574	nonstudy	24months	35	35	35	35	35	35	35	23.052	35	35	28.099	35	30.87	35	35	35	30.583	35	35
575	nonstudy	12months	35	35	33.297	35	35	35	35	35	35	35	32.089	35	20.469	35	35	35	28.828	35	35
576	placebo	0months	35	35	26.395	35	35	35	35	29.59	35	35	21.522	35	32.764	35	35	35	28.983	35	35
577	azithro	0months	35	35	25.053	35	35	35	35	35	35	32.346	25.697	35	22.557	35	35	35	27.198	35	35
578	azithro	0months	35	35	27.356	35	35	35	35	35	35	35	18.343	35	18.511	35	35	35	23.606	35	35
579	azithro	12months	33.601	35	31.978	35	35	35	35	26.59	35	35	19.365	35	23.147	35	35	35	24.968	34.082	35
580	nonstudy	0months	35	35	28.858	35	35	35	35	35	35	35	26.557	35	24.509	35	35	35	28.406	35	35
581	nonstudy	24months	35	35	29.59	35	23.707	35	35	35	35	35	29.353	35	30.368	35	22.057	35	34.092	35	35
582	placebo	12months	35	35	35	35	35	35	35	35	35	35	35	35	35	35	35	35	27.443	35	35
583	azithro	0months	35	35	29.656	35	30.776	35	35	35	35	35	17.451	35	20.304	35	35	35	23.707	35	35
584	placebo	0months	35	25.434	31.604	35	35	35	33.406	35	35	35	21.153	35	21.655	35	35	34.745	29.251	35	35
585	nonstudy	0months	35	35	35	35	35	35	35	35	35	34.156	35	35	20.517	35	35	35	29.577	35	35
586	azithro	24months	19.475	30.08	28.014	35	35	35	35	35	35	35	23.637	35	24.891	35	35	35	26.485	35	35
587	azithro	12months	35	35	27.037	35	35	35	35	32.361	35	35	22.314	35	19.561	35	35	35	25.88	35	35
588	placebo	12months	35	35	35	35	35	35	35	28.984	35	35	20.359	35	24.458	35	35	35	23.877	35	35
589	nonstudy	0months	35	35	35	35	35	35	35	30.618	35	33.349	23.647	23.326	21.646	35	35	34.512	22.222	35	35
590	nonstudy	0months	35	35	35	35	35	35	35	35	35	35	23.577	35	24.283	35	35	35	25.367	35	35
591	azithro	12months	35	35	35	35	35	35	35	33.563	35	35	21.931	20.801	23.901	35	35	35	25.947	35	35
592	nonstudy	12months	35	35	28.243	35	35	35	23.63	35	35	35	20.179	35	20.191	35	35	35	25.803	35	35

593	nonstudy	0months	35	35	35	35	35	33.51	35	35	35	31.348	35	35	35	35	35	35	35	35	35
594	nonstudy	0months	35	35	31.66	35	35	35	35	35	35	35	22.953	35	20.026	35	35	35	23.859	35	35
595	nonstudy	0months	35	35	26.115	35	35	35	35	33.407	35	35	22.941	35	21.718	35	35	35	23.766	35	35
596	azithro	0months	35	35	35	35	35	35	35	35	35	30.133	34.584	35	35	35	35	35	35	35	35
597	azithro	0months	35	35	19.643	35	35	35	35	35	35	35	21.412	35	21.773	35	35	35	24.127	35	35
598	azithro	24months	35	35	26.362	35	35	35	23.529	29.276	35	33.881	20.247	35	21.259	35	35	35	22.553	35	35
599	azithro	12months	35	35	26.654	35	35	35	35	20.413	35	35	25.593	35	22.209	35	35	35	27.338	35	35
600	placebo	12months	26.073	35	27.216	35	35	35	35	32.981	35	35	32.334	35	22.583	35	35	35	30.165	35	35
601	azithro	12months	27.909	33.059	27.553	35	35	35	35	35	35	35	25.49	35	35	35	35	35	35	35	35
602	nonstudy	24months	35	35	26.492	35	35	35	35	35	35	35	21.167	35	23.355	35	35	35	25.522	35	35
603	nonstudy	24months	32.339	35	25.753	35	35	35	35	35	17.076	35	21.308	35	22.942	35	35	35	23.444	35	35
604	nonstudy	24months	35	35	34.342	35	35	35	35	35	35	35	34.769	35	23.657	35	35	35	27.656	35	35
605	placebo	12months	35	35	35	35	35	35	35	26.163	35	35	18.85	35	21.029	35	35	35	22.238	35	35
606	placebo	24months	35	35	32.734	35	35	35	35	31.162	34.731	35	20.63	35	22.961	35	35	35	28.997	35	35
607	nonstudy	0months	35	35	30.638	35	35	35	35	35	20.253	35	20.795	35	22.487	35	35	35	35	34.009	35
608	nonstudy	24months	35	35	29.656	35	35	35	35	35	23.797	35	22.998	35	35	35	35	35	22.064	35	35
609	placebo	12months	35	35	28.455	35	35	35	35	35	35	35	35	35	35	35	35	35	34.557	35	35
610	nonstudy	24months	35	33.372	35	35	35	35	35	35	35	35	32.788	35	27.338	35	26.028	35	32.253	35	35
611	placebo	0months	34.343	35	28.922	35	35	35	35	35	35	35	23.062	35	20.455	35	35	35	22.796	35	35
612	placebo	24months	35	34.442	31.594	35	35	35	35	35	35	17.76	35	35	29.876	35	35	35	35	35	35
613	placebo	12months	35	35	26.404	35	35	35	35	33.105	35	35	25.764	29.461	35	35	35	35	35	35	35
614	nonstudy	24months	35	35	35	35	35	35	35	34.341	35	35	19.309	35	28.435	35	35	35	24.456	35	35
615	nonstudy	0months	35	35	29.36	35	35	35	35	35	35	35	25.75	35	21.894	35	33.246	25.927	27.531	35	35
616	placebo	0months	35	35	35	35	35	33.797	35	35	35	31.556	35	35	35	35	35	35	35	35	35
617	placebo	0months	35	35	35	35	35	32.788	35	35	35	32.47	35	35	35	35	35	35	35	35	35
618	placebo	12months	35	35	35	35	35	35	35	30.969	35	35	35	35	23.558	35	35	35	27.434	35	35
619	azithro	0months	35	35	35	35	35	35	23.888	30.867	35	34.348	19.811	35	22.913	35	35	35	28.542	35	35
620	nonstudy	24months	35	13.533	26.092	35	35	35	35	35	35	35	17.164	35	20.226	35	35	35	23.669	35	35
621	placebo	0months	35	35	29.633	35	35	35	21.756	35	35	35	25.337	35	25.006	35	17.555	35	34.139	35	35
622	nonstudy	24months	35	35	35	35	35	35	35	31.432	35	35	20.644	35	19.949	35	35	31.031	25.48	35	35
623	azithro	24months	35	35	27.183	35	35	35	35	35	35	35	22.907	35	22.263	35	35	35	26.661	35	35
624	nonstudy	0months	35	35	28.663	35	35	35	20.583	35	35	35	17.64	35	24.761	35	35	35	28.977	35	35
625	azithro	0months	35	35	32.705	35	35	35	35	35	35	35	16.739	35	18.797	35	35	35	20.96	35	35
626	nonstudy	24months	35	35	34.069	35	35	35	35	32.724	35	35	17.731	35	18.207	35	35	30.443	24.747	33.944	35
627	nonstudy	0months	35	35	35	35	35	31.723	35	35	35	30.286	35	35	35	35	35	35	35	35	35
628	nonstudy	24months	35	35	28.962	35	35	35	35	35	35	35	25.997	35	21.86	35	35	35	33.277	35	35
629	nonstudy	12months	35	35	25.704	35	35	35	35	34.06	35	35	32.672	35	26.106	35	35	35	27.066	35	35
630	nonstudy	0months	35	34.399	32.03	35	35	35	35	32.56	35	35	21.403	35	21.231	35	35	30.171	24.425	30.69	35
631	azithro	24months	35	35	35	35	35	35	35	35	35	35	35	35	35	35	35	25.037	35	35	35
632	placebo	12months	34.226	35	32.155	35	35	35	35	33.324	35	35	26.546	35	21.095	35	35	30.58	29.363	32.607	35
633	nonstudy	12months	35	26.759	35	35	35	35	35	33.085	35	35	20.782	35	24.637	35	35	35	28.136	35	35
634	azithro	24months	29.814	35	31.498	35	35	35	35	35	35	35	20.02	35	26.576	35	35	35	24.016	35	35
635	nonstudy	0months	35	35	35	35	35	35	35	35	35	35	19.016	19.616	21.727	35	35	35	26.102	35	35
636	azithro	24months	35	15.205	35	35	35	35	35	35	23.583	35	21.492	35	35	35	35	35	29.308	35	35
637	nonstudy	24months	35	35	20.834	35	35	35	35	33.096	35	35	35	35	35	35	35	32.925	33.596	35	35
638	placebo	24months	35	35	33.675	35	35	35	35	26.73	35	35	35	35	30.391	35	35	35	22.905	35	35
639	azithro	24months	35	35	29.303	35	35	35	35	35	34.57	35	20.11	35	35	35	35	35	21.701	35	35
640	azithro	12months	29.904	35	33.09	35	35	35	35	33.014	35	35	23.031	35	24.192	35	35	35	25.644	35	35
641	nonstudy	24months	35	35	31.348	35	35	35	35	35	35	35	21.264	35	19.674	35	25.74	35	22.206	35	35
642	nonstudy	24months	32.086	35	31.33	35	35	35	35	30.145	35	35	18.38	35	23.118	35	35	35	24.035	35	35
643	nonstudy	12months	35	35	35	35	35	35	35	33.215	35	35	24.846	35	26.251	35	35	35	26.391	35	35
644	nonstudy	24months	32.467	35	27.194	35	35	35	35	27.676	35	35	26.724	35	25.234	35	35	35	26.467	35	35
645	placebo	24months	32.09	32.348	35	35	35	35	19.671	35	35	35	19.905	35	31.544	35	35	35	23.962	35	35
646	placebo	0months	35	35	35	35	35	35	35	35	35	23.869	21.904	35	21.67	35	35	35	24.195	35	35
647	nonstudy	24months	35	35	35	35	35	35	35	32.057	35	35	28.106	35	35	35	35	31.113	35	35	35
648	nonstudy	24months	35	35	28.525	35	35	35	35	35	35	35	20.46	35	23.334	35	35	35	24.642	35	35
649	nonstudy	0months	35	35	35	35	35	35	35	35	35	35	19.984	32.004	21.865	35	35	35	25.489	35	35
650	nonstudy	24months	35	35	25.179	35	35	35	35	35	35	35	35	35	23.979	35	35	35	30.167	35	35
651	nonstudy	0months	35	35	34.772	35	35	35	35	31.359	35	35	22.958	35	25.541	35	35	35	24.472	35	35
652	azithro	24months	22.184	35	25.406	35	35	35	35	29.34	35	35	21.73	35	20.808	35	35	35	22.373	27.59	35
653	nonstudy	24months	35	35	28.033	35	35	35	35	30.733	34.828	35	16.962	35	35	35	35	35	22.126	35	35
654	placebo	0months	35	35	33.95	35	35	35	35	35	35	28.449	22.228	35	24.758	35	35	35	29.29	35	35
655	placebo	0months	30.565	35	35	35	35	35	35	35	35	35	17.179	35	21.043	35	35	35	34.373	35	35
656	azithro	12months	35	35	35	35	35	35	35	35	35	35	23.939	35	35	35	35	35	24.177	35	35
657	nonstudy	12months	35	35	35	35	35	35	35	35	35	35	35	35	35	35	35	35	35	35	35
658	placebo	12months	35	35	29.48	35	35	35	34.081	32.639	35	35	21.948	35	20.982	35	23.435	35	25.361	32.859	35

659	placebo	0months	35	35	35	35	35	35	35	35	35	31.49	35	35	35	35	35	35	35	35	35
660	nonstudy	24months	35	35	35	35	35	35	35	35	35	35	21.318	35	24.966	35	35	35	24.955	35	35
661	placebo	12months	35	35	27.347	24.328	35	35	35	33.572	35	35	22.695	35	35	35	35	35	24.218	35	35
662	nonstudy	0months	35	35	35	35	35	33.428	35	35	35	31.266	35	35	35	35	35	35	35	35	35
663	nonstudy	12months	35	35	35	35	35	35	35	31.619	35	35	35	35	35	35	35	35	35	35	35
664	nonstudy	12months	35	35	32.45	35	35	35	35	27.45	35	35	33.059	35	32.654	35	35	35	20.975	35	35
665	nonstudy	24months	25.388	35	32.273	35	35	35	35	27.548	35	35	19.259	35	23.01	35	35	35	25.35	35	35
666	placebo	24months	35	35	35	35	35	35	35	35	16.963	32.309	25.251	35	35	35	35	35	20.694	33.771	35
667	nonstudy	24months	35	35	33.593	35	35	35	35	27.494	34.697	35	19.421	35	23.816	35	35	35	21.956	35	35
668	azithro	24months	35	35	35	35	35	35	23.816	35	35	35	19.594	35	35	35	35	35	25.159	35	35
669	nonstudy	24months	35	35	29.103	35	35	35	35	35	35	35	24.042	35	21.378	35	35	35	25.156	35	35
670	nonstudy	12months	35	35	26.342	35	35	35	35	32.821	35	35	29.584	35	35	35	34.384	35	26.54	35	35
671	placebo	24months	35	35	32.457	35	35	35	18.109	35	35	35	17.341	35	21.003	35	35	35	32.921	35	35
672	azithro	0months	35	35	35	35	35	35	35	34.071	35	35	35	35	35	35	35	22.513	35	35	35
673	nonstudy	0months	35	35	29.594	35	35	35	35	35	35	35	19.412	35	24.781	35	35	35	23.231	35	35
674	azithro	0months	35	35	35	35	35	29.683	35	35	35	29.552	35	35	35	35	35	35	35	35	35
675	azithro	0months	35	35	35	35	35	35	35	35	35	35	19.889	35	21.171	35	35	35	28.471	35	35
676	azithro	24months	35	35	26.37	35	35	35	33.273	35	35	35	24.553	35	20.997	35	35	35	24.832	35	35
677	placebo	0months	34.128	35	35	35	35	35	35	35	35	31.788	29.865	35	23.698	35	35	35	25.288	35	35
678	nonstudy	24months	35	35	31.388	35	35	35	35	33.823	35	35	21.122	35	22.209	35	35	35	25.611	35	35
679	nonstudy	0months	35	35	23.691	27.642	35	35	35	35	35	35	20.833	35	27.553	35	30.999	35	21.515	35	35
680	azithro	24months	34.578	35	29.588	35	35	35	35	35	19.135	35	22.065	35	26.972	35	35	35	24.517	35	35
681	nonstudy	0months	35	35	24.744	35	35	35	35	27.506	35	35	21.378	35	22.219	35	35	35	24.084	35	35
682	azithro	24months	18.215	35	29.491	35	35	35	35	35	35	35	22.642	35	26.703	35	35	35	23.94	35	35
683	nonstudy	0months	24.682	35	25.34	35	35	35	35	28.049	33.592	35	18.354	35	28.669	35	35	35	25.467	35	35
684	azithro	24months	35	35	32.643	35	35	35	35	21.445	35	35	33.212	35	25.261	35	35	35	26.385	35	35
685	nonstudy	0months	19.712	35	26.67	35	35	35	35	35	35	35	22.269	35	22.972	35	35	35	26.213	35	35
686	placebo	0months	15.623	35	31.194	35	35	35	35	35	35	27.993	24.791	35	22.272	35	35	35	28.018	35	35
687	placebo	12months	35	35	35	35	35	35	35	32.215	35	35	16.271	33.079	15.408	35	35	35	19.543	35	35
688	nonstudy	24months	35	35	35	35	35	35	27.7	35	35	35	25.981	35	26.121	35	35	35	29.824	35	35
689	placebo	0months	35	29.431	31.952	35	35	35	35	35	35	35	23.738	35	21.061	35	35	35	26.313	35	35
690	placebo	24months	35	35	21.082	35	35	35	35	29.873	35	35	18.545	35	21.469	35	35	35	23.908	35	35
691	nonstudy	24months	35	35	35	35	35	35	35	35	35	35	27.602	35	35	35	35	35	32.443	35	35
692	azithro	0months	35	35	35	35	29.621	35	35	35	35	35	20.619	35	21.137	35	35	35	22.506	35	35
693	azithro	24months	35	35	35	35	35	35	35	35	27.94	35	18.63	35	35	35	35	31.321	35	35	35
694	placebo	24months	35	35	29.866	35	35	32.252	35	35	35	35	21.681	35	21.753	35	30.479	35	23.959	35	35
695	azithro	24months	34.287	35	32.536	35	35	35	35	35	35	35	25.383	35	35	35	35	34.749	25.296	35	35
696	nonstudy	0months	35	35	35	35	35	35	35	35	35	35	20.888	35	21.026	35	35	35	27.771	35	35
697	nonstudy	0months	35	35	35	35	35	35	35	35	35	34.879	35	35	35	35	35	35	35	35	35
698	nonstudy	24months	35	35	23.982	35	35	35	35	34.489	35	35	24.229	35	23.583	35	35	35	23.855	35	35
699	azithro	0months	35	35	35	35	35	35	35	35	35	35	35	35	35	35	35	35	35	35	35
700	nonstudy	24months	33.225	35	35	35	35	20.372	35	35	30.426	34.619	20.293	35	28.383	35	35	35	25.57	35	35
701	azithro	0months	35	35	30.794	35	35	35	35	34.445	35	35	23.178	35	23.321	35	32.233	35	25.972	35	35
702	nonstudy	24months	15.63	32.607	32.428	35	35	35	35	35	35	35	28.708	35	35	35	35	35	35	35	35
703	azithro	0months	35	35	28.791	35	35	35	30.742	35	35	35	18.911	35	24.441	35	35	35	23.3	35	35
704	placebo	0months	35	35	27.285	35	35	35	35	35	35	35	21.607	35	19.098	35	35	35	23.832	35	35
705	placebo	24months	35	35	35	35	35	35	35	35	35	35	23.259	35	26.102	35	35	29.424	26.428	35	35
706	nonstudy	24months	35	35	27.755	35	35	35	35	25.15	34.426	35	21.103	35	30.022	35	35	35	25.6	35	35
707	nonstudy	12months	35	33.549	35	35	35	35	35	35	35	35	23.569	31.952	24.852	35	35	35	20.538	35	35
708	azithro	12months	35	35	21.334	35	35	35	35	35	35	35	30.495	35	22.889	35	35	35	27.999	35	35
709	placebo	0months	35	35	26.963	35	35	35	35	34.555	35	35	32.321	35	29.29	35	35	35	34.162	35	35
710	placebo	24months	35	35	35	35	35	35	35	35	35	35	25.014	35	35	35	25.474	35	29.111	35	35
711	nonstudy	24months	35	35	35	35	35	35	35	35	19.26	35	35	35	20.562	35	35	23.051	20.71	35	35
712	nonstudy	12months	35	35	35	35	35	35	33.302	33.675	35	35	25.468	35	35	35	35	35	24.362	35	35
713	nonstudy	12months	35	35	35	35	35	35	35	35	35	35	22.042	35	24.548	35	35	35	26.085	35	35
714	placebo	0months	35	35	32.052	35	35	35	22.313	35	35	35	24.705	35	23.142	35	35	35	24.828	35	35
715	placebo	0months	33.1	34.424	26.22	35	35	35	35	29.998	35	35	20.468	35	24.926	35	25.718	35	24.425	35	35
716	azithro	0months	31.661	35	31.993	35	22.29	35	35	35	35	35	18.432	35	25.673	35	35	35	25.516	35	35
717	azithro	24months	35	35	35	35	35	35	35	35	35	35	35	35	35	35	35	27.771	35	35	35
718	nonstudy	0months	35	35	35	35	35	35	35	26.476	35	35	35	35	22.928	35	31.289	35	22.07	35	35
719	nonstudy	12months	35	35	33.413	35	35	35	35	31.417	35	35	35	35	25.266	35	35	35	28.987	35	35
720	azithro	0months	33.145	35	29.628	35	35	35	35	35	19.726	35	24.987	35	22.461	35	35	35	26.769	35	35
721	placebo	12months	35	35	35	35	35	35	35	27.599	35	35	20.131	35	22.471	35	35	35	24.683	35	35
722	azithro	0months	35	35	23.804	35	35	35	35	33.368	19.88	35	23.8	35	20.565	35	35	35	19.207	35	35
723	placebo	12months	35	35	27.941	35	23.308	35	35	35	35	35	28.159	35	21.751	35	35	35	26.726	35	35
724	placebo	0months	35	35	30.968	35	35	35	35	34.645	35	30.647	26.18	35	29.814	35	35	35	26.152	35	35

725	nonstudy	24months	35	35	35	35	35	35	35	35	35	26.747	35	35	30.144	35	22.374	33.577	32.713	35	35
726	placebo	0months	32.248	35	35	35	35	35	35	32.009	35	35	20.045	35	23.958	35	35	35	27.136	35	35
727	placebo	0months	32.271	35	33.432	35	35	35	35	33.73	35	35	21.19	35	24.746	35	35	35	26.01	35	35
728	nonstudy	12months	35	35	35	35	35	35	35	23.762	35	35	21.731	22.747	24.893	35	35	35	25.639	35	35
729	nonstudy	24months	35	35	35	35	35	35	27.946	35	35	35	16.901	35	19.524	35	35	35	35	35	35
730	azithro	24months	35	35	29.163	35	35	35	35	35	35	35	21.856	35	23.436	35	35	35	24.15	35	35
731	placebo	0months	35	35	25.049	35	35	35	35	27.933	35	35	17.697	35	23.905	35	35	35	27.833	35	35
732	nonstudy	12months	35	35	21.969	35	35	35	35	27.112	35	35	30.709	35	24.603	35	35	35	25.222	35	35
733	azithro	0months	33.661	35	29.396	35	35	35	33.693	35	35	35	17.864	35	18.657	35	35	35	20.244	35	35
734	azithro	0months	33.583	35	28.351	35	35	35	35	23.211	35	35	35	35	22.551	35	35	35	25.522	35	35
735	nonstudy	24months	35	35	30.87	35	35	35	35	20.674	35	35	26.567	35	21.465	35	35	35	24.588	35	35
736	placebo	12months	35	35	35	35	35	35	35	30.53	35	33.927	25.388	35	25.876	35	35	35	25.172	35	35
737	nonstudy	0months	35	35	33.507	35	28.181	35	35	35	35	35	22.562	35	23.308	35	35	35	24.44	35	35
738	azithro	12months	35	35	35	35	35	35	35	35	35	35	25.743	35	29.581	35	35	35	25.513	35	35
739	nonstudy	24months	35	32.739	35	35	35	35	35	20.232	35	35	34.343	35	25.871	35	35	33.512	31.013	35	35
740	azithro	24months	35	35	28	35	35	35	35	31.772	35	35	22.072	35	22.684	35	35	34.382	24.906	33.924	35
741	nonstudy	0months	35	35	22.222	35	35	35	35	35	35	29.839	24.075	35	20.45	35	35	35	26.779	35	35
742	azithro	24months	35	35	33.857	35	35	35	35	35	17.998	35	18.96	35	26.331	35	35	35	26.913	35	35
743	placebo	0months	35	35	24.587	26.137	35	35	35	35	35	33.531	22.975	35	23.44	35	35	35	23.854	35	35
744	placebo	0months	23.639	35	35	35	35	35	35	31.096	35	35	18.396	35	23.44	35	29.017	35	23.459	35	35
745	nonstudy	0months	35	35	35	35	35	35	35	35	35	35	21.445	29.051	22.131	35	35	35	24.999	35	35
746	nonstudy	0months	35	35	35	35	35	19.564	35	35	35	35	35	35	23.882	35	35	35	30.446	35	35
747	nonstudy	0months	35	35	28.923	35	35	35	35	35	35	35	20.147	35	21.234	35	35	35	24.24	35	35
748	azithro	24months	35	35	35	35	35	35	21.577	35	34.397	35	19.337	35	22.462	35	35	35	24.1	35	35
749	nonstudy	24months	21.801	35	27.078	35	35	35	35	32.529	35	35	21.561	35	24.131	35	35	35	27.343	35	35
750	nonstudy	0months	35	35	35	35	35	35	35	35	35	35	19.74	35	22.907	35	35	35	27.625	35	35
751	nonstudy	0months	31.01	35	31.517	35	35	35	34.653	35	35	35	19.36	35	21.711	35	35	35	27.433	35	35
752	nonstudy	24months	35	35	33.177	35	35	35	35	20.573	35	35	25.481	35	22.918	35	35	35	24.803	35	35
753	nonstudy	24months	35	35	35	35	35	35	35	32.946	35	35	18.697	35	20.154	35	35	35	22.917	35	35
754	azithro	0months	13.049	35	28.491	35	35	35	35	35	35	35	27.887	35	33.843	35	35	35	35	35	35
755	nonstudy	12months	35	15.07	35	35	35	35	35	35	35	35	24.011	35	24.857	35	35	33.127	26.57	35	35
756	azithro	24months	35	35	32.258	35	35	35	28.218	35	35	35	21.122	35	28.669	35	35	35	24.541	35	35
757	placebo	24months	24.19	35	24.245	35	35	35	35	27.053	35	13.82	19.264	25.189	21.115	35	35	35	22.306	35	35
758	placebo	0months	35	35	35	35	35	35	35	25	35	35	18.564	35	25.055	35	35	35	23.048	35	35
759	nonstudy	24months	35	35	29.811	35	35	35	35	26.834	35	35	21.369	35	35	35	35	35	23.308	35	35
760	nonstudy	0months	35	35	35	35	35	20.704	35	35	35	35	24.476	35	24.672	35	35	35	24.246	35	35
761	nonstudy	0months	35	35	27.747	35	35	35	35	35	35	35	18.843	35	20.534	35	35	35	26.128	35	35
762	nonstudy	0months	35	31.011	22.822	29.975	35	35	35	27.368	33.555	35	27.015	35	23.732	35	18.763	35	24.726	35	35
763	azithro	24months	35	35	28.967	35	35	35	35	26.099	35	35	17.475	35	20.202	35	35	35	23.958	35	35
764	nonstudy	12months	35	35	35	35	35	35	35	26.437	35	35	35	35	35	35	35	35	35	35	35
765	nonstudy	0months	31.098	35	30.285	35	35	35	35	30.381	33.294	35	20.286	35	26.738	35	35	35	26.442	35	35
766	placebo	0months	34.54	33.164	26.47	35	35	35	35	32.865	35	35	20.928	35	23.506	35	35	35	25.682	35	35
767	azithro	24months	35	35	25.591	35	35	35	35	35	35	35	28.693	35	25.666	35	35	35	23.677	35	35
768	nonstudy	0months	30.122	35	25.771	35	35	35	35	35	35	35	23.907	35	20.489	35	35	35	26.836	35	35
769	nonstudy	24months	35	35	35	35	23.584	35	35	35	35	35	21.26	35	20.331	35	35	35	30.364	35	35
770	nonstudy	12months	35	35	35	33.074	35	35	35	35	35	35	24.578	35	31.853	35	35	35	32.864	35	35
771	nonstudy	0months	35	35	30.471	35	35	17.244	32.282	27.881	34.821	35	30.69	35	23.202	35	35	35	25.585	35	35
772	nonstudy	24months	35	35	27.681	35	35	35	35	28.029	35	35	19.743	35	29.832	35	35	35	23.456	35	35
773	nonstudy	24months	35	35	22.659	35	35	35	35	27.838	35	35	21.255	35	24.483	35	24.03	35	22.818	35	35
774	nonstudy	24months	35	35	35	35	35	35	35	35	35	35	35	35	35	35	35	26.818	35	35	35
775	nonstudy	12months	35	35	35	35	35	35	35	35	35	35	23.608	35	20.945	35	35	35	23.694	35	35
776	azithro	0months	35	35	27.802	35	35	35	35	27.636	35	35	22.296	35	23.289	35	20.327	35	24.912	35	35
777	azithro	24months	20.644	35	29.462	35	35	35	34.411	35	35	34.705	25.024	27.849	23.353	35	35	33.758	26.142	35	35
778	placebo	24months	35	35	35	35	35	35	35	35	35	35	20.112	24.094	20.963	35	35	27.709	34.088	35	35
779	nonstudy	24months	35	30.112	27.179	35	35	35	19.994	35	35	35	20.65	35	22.225	35	35	35	25.499	35	35
780	azithro	24months	35	35	26.908	35	35	35	35	23.66	35	35	20.307	35	22.556	35	35	35	22.681	32.697	35
781	placebo	0months	35	35	29.955	35	35	35	35	26.149	35	35	21.805	35	22.564	35	35	35	25.089	35	35
782	azithro	24months	35	35	35	35	35	35	35	35	35	35	26.103	35	24.618	35	35	35	26.929	35	35
783	azithro	24months	15.428	35	27.841	35	35	35	35	35	35	28.404	35	35	21.609	35	35	35	25.898	35	35
784	nonstudy	24months	35	35	26.235	35	35	35	35	31.693	35	35	21.053	35	22.369	35	35	35	25.722	35	35
785	azithro	24months	35	35	25.591	35	35	35	35	35	35	27.219	23.841	35	21.465	35	35	35	31.953	35	35
786	azithro	12months	32.731	35	28.931	35	35	35	35	32.397	35	35	18.25	35	24.277	35	35	35	23.907	35	35
787	placebo	24months	35	35	29.561	35	35	35	35	35	35	35	35	35	21.558	35	35	35	24.933	35	35
788	azithro	0months	35	35	35	35	35	35	35	27.975	35	35	22.899	35	30.675	35	35	35	25.141	35	35
789	azithro	0months	35	35	35	35	35	29.66	35	35	35	31.857	35	35	35	35	35	34.471	35	35	35
790	placebo	12months	35	35	35	34.194	35	35	34.574	35	35	35	35	35	26.379	35	35	35	29.721	35	35

791	nonstudy	24months	35	35	30.281	35	35	35	35	35	20.163	35	20.849	21.446	23.072	35	35	35	24.234	35	35
792	azithro	0months	35	35	30.544	35	35	35	35	35	35	35	22.609	35	22.875	35	35	35	24.909	29.208	35
793	nonstudy	0months	35	35	25.42	35	35	35	35	35	35	35	17.747	35	33.004	35	18.644	35	18.351	35	35
794	nonstudy	0months	35	34.166	28.631	35	35	35	35	27.511	35	35	20.26	35	26.467	35	35	35	26.99	35	35
795	azithro	24months	25.817	35	35	35	35	35	35	35	35	35	26.403	35	31.922	35	35	35	35	35	35
796	nonstudy	12months	32.24	35	28.375	35	35	35	27.455	29.216	35	35	34.417	35	20.387	35	35	35	24.801	35	35
797	azithro	24months	35	35	28.695	35	35	35	35	18.359	35	35	33.25	35	35	35	35	35	29.949	35	35
798	azithro	0months	35	35	28.82	35	35	35	34.782	26.388	35	35	22.371	35	21.686	35	35	35	25.126	35	35
799	nonstudy	0months	35	33.457	27.911	35	35	35	35	35	35	35	23.722	35	23.85	35	35	35	21.971	35	35
800	azithro	24months	35	35	33.177	35	35	35	35	26.995	34.605	35	20.347	35	28.703	35	35	35	23.16	35	35
801	azithro	0months	31.881	35	30.918	35	35	35	35	35	35	35	22.1	20.959	24.391	35	35	35	23.836	35	35
802	nonstudy	0months	35	32.237	35	35	35	35	35	35	35	35	20.731	35	23.45	35	35	35	26.068	35	35
803	azithro	24months	35	32.73	35	35	35	35	35	23.095	34.47	35	35	35	35	35	30.816	35	26.906	35	35
804	azithro	0months	35	35	33.641	35	35	35	35	24.569	35	35	16.688	35	25.489	35	35	35	35	35	35
805	nonstudy	24months	35	35	35	35	35	35	35	34.167	35	35	30.192	35	35	35	35	33.574	30.947	35	35
806	placebo	0months	35	35	35	35	35	35	35	35	35	27.456	33.142	35	25.522	35	35	35	27.042	35	35
807	azithro	24months	35	35	35	35	35	35	32.701	35	35	35	34.231	35	35	35	35	35	34.53	35	35
808	nonstudy	0months	35	34.275	27.625	35	35	35	25.606	35	35	35	24.992	35	35	35	35	35	35	35	35
809	nonstudy	0months	35	35	26.578	35	35	35	35	35	35	35	16.157	35	19.374	35	35	35	21.63	35	35
810	azithro	0months	35	35	35	35	35	35	35	35	35	35	19.901	35	33.173	35	35	35	33.02	35	35
811	nonstudy	24months	35	35	27.328	35	35	35	35	22.473	35	35	21.388	35	24.376	35	35	33.492	30.76	35	35
812	placebo	0months	35	35	35	35	35	35	35	35	35	35	20.046	19.853	21.88	35	35	35	27.936	35	35
813	nonstudy	24months	35	35	29.816	35	35	35	35	26.956	35	35	22.173	35	21.587	35	35	35	24.817	35	35
814	azithro	0months	32.862	35	30.065	35	35	35	35	35	35	35	23.126	35	24.876	35	35	35	26.681	35	35
815	nonstudy	24months	23.769	35	25.843	35	35	35	35	35	35	35	20.195	35	21.286	35	35	35	31.157	35	35
816	nonstudy	0months	35	35	35	35	35	35	35	35	35	35	30.837	35	25.107	35	35	35	28.155	35	35
817	nonstudy	24months	35	35	25.041	35	35	35	35	29.982	35	35	19.059	35	24.426	35	33.543	35	23.666	35	35
818	placebo	0months	35	35	26.277	35	35	35	35	35	19.136	33.519	26.813	35	26.192	35	35	35	26.498	35	35
819	placebo	0months	34.872	35	35	35	35	35	35	35	34.885	35	17.568	35	35	35	35	24.63	24.268	35	35
820	nonstudy	0months	31.218	35	25.037	35	35	35	35	35	35	35	17.934	35	19.771	35	35	35	23.154	35	35
821	nonstudy	0months	35	35	35	35	35	35	35	35	35	35	19.507	19.674	21.293	35	35	35	27.249	35	35
822	placebo	24months	35	35	28.441	35	35	35	35	24.754	35	30.284	26.025	35	23.959	35	35	35	24.881	35	35
823	placebo	12months	35	35	35	35	35	35	35	35	35	35	33.156	35	29.046	35	35	35	35	35	35
824	placebo	12months	35	35	27.518	35	35	35	35	35	35	35	22.686	35	24.53	35	35	35	26.851	35	35
825	placebo	24months	31.497	35	30.938	35	35	35	35	23.834	35	35	22.879	35	24.152	35	35	35	24.402	35	35
826	placebo	0months	35	35	35	35	35	32.9	35	35	35	31.097	35	35	35	35	35	35	35	35	35
827	azithro	0months	33.894	35	27.996	35	35	35	20.977	35	35	35	20.769	35	28.665	35	35	35	23.138	35	35
828	placebo	12months	35	35	35	35	35	35	35	35	35	35	25.369	35	24.037	35	35	35	26.802	35	35
829	azithro	12months	35	35	25.634	35	35	35	35	28.692	35	35	21.558	35	21.89	35	33.133	35	23.468	35	35
830	placebo	24months	35	19.391	35	35	35	35	35	35	35	35	26.738	35	35	35	35	35	30.34	35	35
831	nonstudy	0months	35	35	28.587	35	35	35	35	35	34.289	35	21.909	35	19.847	35	35	35	21.552	35	35
832	nonstudy	12months	35	35	35	35	35	35	35	35	35	35	29.898	35	26.521	35	35	35	25.204	35	35
833	azithro	24months	35	35	35	35	35	35	35	29.492	35	35	30.128	35	35	35	35	29.471	21.864	35	35
834	nonstudy	24months	35	35	31.177	35	35	35	35	35	34.328	32.724	19.803	35	31.004	35	35	35	24.414	35	35
835	placebo	24months	35	35	29.101	35	35	35	35	35	35	35	24.73	35	25.311	35	35	35	23.572	35	35
836	placebo	12months	35	35	30.205	35	35	35	35	35	35	35	26.192	35	19.275	35	35	35	20.642	35	35
837	nonstudy	24months	33.472	35	31.888	35	19.009	35	35	35	35	35	25.002	35	19.664	35	20.309	34.642	28.778	35	35
838	azithro	12months	35	33.387	24.33	35	35	35	33.901	25.719	35	35	20.139	35	33.428	35	35	35	25.988	35	35
839	azithro	0months	35	14.447	26.644	35	35	35	33.817	25.233	35	35	20.916	35	20.251	35	35	35	21.753	35	35
840	azithro	0months	29.995	35	23.745	35	35	35	35	35	35	35	28.504	35	22.718	35	32.378	33.879	30.153	35	35
841	azithro	24months	35	35	35	35	35	35	35	35	35	29.502	21.685	35	19.2	35	35	31.68	30.556	35	35
842	nonstudy	24months	35	34.32	32.193	35	35	35	35	35	17.486	35	23.576	35	24.579	35	35	35	22.378	35	35
843	azithro	12months	32.549	35	29.131	35	35	35	35	30.12	35	35	22.701	35	20.633	35	35	35	26.533	35	35
844	placebo	24months	30.667	35	35	35	35	35	35	35	35	33.624	32.113	35	35	35	35	35	23.988	35	35
845	nonstudy	0months	30.824	35	33.295	28.801	35	35	35	35	35	35	21.848	35	26.676	35	35	35	28.268	32.085	35
846	nonstudy	0months	35	35	26.26	35	21.177	35	35	35	35	35	21.528	35	25.287	35	35	35	26.194	32.61	35
847	azithro	12months	35	35	33.52	35	35	35	35	31.79	35	35	21.206	35	22.436	35	35	35	21.65	35	35
848	azithro	24months	27.701	35	27.195	35	35	35	35	35	35	35	23.869	35	24.419	35	35	35	27.799	35	35
849	azithro	0months	31.11	35	23.513	35	35	35	35	32.257	33.682	35	22.338	35	22.518	35	35	35	25.527	35	35
850	azithro	0months	35	34.224	27.396	35	35	35	35	29.117	35	35	21.394	35	25.656	35	35	35	23.65	35	35
851	placebo	12months	35	35	30.653	35	35	35	35	35	35	33.055	30.431	35	26.602	35	35	35	29.886	35	35
852	azithro	0months	35	35	35	35	35	35	35	24.6	35	35	18.101	35	35	35	35	35	25.361	35	35
853	placebo	24months	35	35	34.621	35	35	35	35	35	35	35	35	35	26.106	35	35	35	25.697	35	35
854	placebo	12months	35	35	24.466	35	35	35	35	28.261	35	35	18.333	35	21.547	35	35	35	22.947	35	35
855	nonstudy	0months	35	35	35	35	35	35	35	24.418	35	35	20.657	35	35	35	35	35	24.962	35	35
856	nonstudy	0months	30.224	35	25.172	35	35	35	35	28.983	35	35	22.364	35	23.126	35	35	35	25.992	35	35

857	placebo	24months	35	35	31.591	35	35	35	35	26.356	35	35	21.794	35	24.334	35	35	35	24.165	35	35
858	nonstudy	24months	35	35	35	35	35	35	29.867	35	35	35	20.873	35	24.241	35	18.508	35	22.629	35	35
859	nonstudy	24months	35	35	35	35	35	35	35	35	35	35	35	35	35	35	35	26.353	35	35	35
860	nonstudy	0months	32.375	28.398	25.91	35	35	35	35	35	34.506	35	15.96	35	20.655	35	17.8	35	22.125	35	35
861	nonstudy	0months	35	35	25.019	35	35	35	35	35	35	35	25.142	35	26.492	35	35	35	24.522	35	35
862	placebo	0months	35	35	34.865	35	35	35	35	35	20.041	35	18.257	33.11	20.915	35	34.293	35	22.534	35	35
863	azithro	24months	35	35	33.753	35	35	35	35	35	17.808	35	21.8	35	22.311	35	35	35	23.514	35	35
864	placebo	12months	35	34.262	25.74	35	35	35	35	27.632	35	35	20.245	35	23.323	35	35	35	24.811	35	33.706
865	nonstudy	24months	35	35	25.567	35	35	35	24.99	35	34.314	35	22.172	35	30.264	35	35	35	26.045	35	35
866	nonstudy	12months	35	35	27.848	35	35	35	35	35	35	35	20.217	35	20.041	35	23.175	35	22.579	34.029	35
867	nonstudy	0months	35	35	35	35	35	35	22.846	35	34.717	35	30.431	35	22.41	35	35	35	23.161	35	35
868	placebo	24months	35	35	35	35	35	35	24.366	35	35	35	30.188	35	28.968	35	35	35	28.844	35	35
869	nonstudy	24months	35	35	35	35	35	35	35	35	35	35	35	35	35	35	35	27.534	35	35	35
870	nonstudy	12months	35	35	35	35	35	35	35	35	35	35	35	35	35	35	35	35	35	35	35
871	azithro	24months	28.425	35	32.972	35	35	35	35	26.3	35	35	21.221	35	23.328	35	35	35	25.499	35	35
872	placebo	0months	35	35	25.84	35	35	35	35	33.874	35	35	22.09	35	35	35	35	35	23.215	35	35
873	placebo	0months	35	35	35	35	35	35	35	26.335	35	35	21.293	35	27.62	35	35	35	22.425	35	35
874	azithro	12months	23.061	35	35	35	35	35	35	31.955	35	35	30.557	35	21.514	35	35	35	29.979	35	35
875	nonstudy	0months	35	35	35	35	35	35	35	29.233	35	35	23.865	35	23.162	35	35	34.733	23.327	35	35
876	azithro	0months	35	29.853	24.222	35	35	35	28.841	35	35	35	19.816	35	21.934	35	35	35	23.078	35	35
877	nonstudy	12months	35	35	30.484	35	35	35	35	31.473	35	35	20.048	35	35	35	35	35	26.073	35	35
878	nonstudy	0months	35	35	29.99	35	35	35	22.948	35	35	35	25.127	35	24.001	35	35	35	24.889	35	35
879	azithro	12months	35	35	34.443	35	35	35	35	35	35	35	25.353	35	19.574	35	35	35	26.517	35	35
880	azithro	12months	33.491	35	32.641	35	35	35	35	29.065	35	35	18.761	35	21.795	35	35	35	25.142	35	35
881	nonstudy	0months	35	35	29.705	35	35	35	35	35	35	35	25.125	35	24.57	35	35	33.505	25.559	35	31.821
882	azithro	0months	35	35	31.091	35	20.776	35	35	35	35	35	23.372	35	21.725	35	35	26.41	30.212	35	35
883	nonstudy	0months	35	35	27.577	35	35	35	20.034	29.637	35	35	19.268	35	24.624	35	35	35	23.981	35	35
884	nonstudy	12months	35	35	29.828	35	35	35	35	35	35	35	35	35	26.538	35	35	35	24.381	35	35
885	placebo	12months	27.413	35	35	35	35	35	25.483	35	35	35	18.778	35	23.055	35	35	35	28.696	35	35
886	azithro	24months	35	35	28.97	35	35	35	35	31.968	30.25	35	35	35	21.558	35	35	35	26.862	35	35
887	nonstudy	0months	34.159	35	34.719	35	35	35	35	35	35	35	35	35	22.977	35	35	33.414	33.624	35	35
888	placebo	24months	35	35	35	35	35	35	35	31.223	35	35	35	35	21.848	35	35	34.63	35	34.567	35
889	placebo	12months	25.61	35	35	35	35	35	35	30.308	35	33.295	35	35	19.276	35	35	35	27.692	35	35
890	azithro	12months	18.96	35	26.321	35	35	35	35	35	35	35	31.92	35	21.543	35	35	35	26.233	35	35
891	nonstudy	24months	35	35	31.847	35	35	35	35	28.354	35	35	22.579	35	25.267	35	35	35	26.4	35	35
892	placebo	12months	24.167	35	29.034	35	35	35	22.524	35	35	35	24.898	35	20.277	35	35	35	24.748	34.173	35
893	azithro	24months	35	35	35	35	35	35	35	35	35	35	24.475	35	24.911	35	35	32.216	31.406	32.945	35
894	azithro	24months	35	35	28.87	35	35	35	31.661	35	35	35	22.22	35	21.442	35	35	35	24.447	35	35
895	placebo	24months	35	35	35	35	35	35	27.99	35	35	35	33.35	35	24.912	35	35	35	33.327	35	35
896	nonstudy	24months	35	35	35	35	35	24.426	35	35	35	35	15.846	35	26.311	35	35	35	22.327	35	35
897	placebo	0months	24.974	35	28.646	35	35	35	35	35	35	35	17.755	20.715	19.586	35	35	35	23.365	35	35
898	azithro	12months	35	35	35	35	35	35	33.791	35	35	35	25.377	28.357	25.844	35	35	35	25.787	35	35
899	nonstudy	0months	35	35	31.29	35	35	35	35	28.571	35	35	19.778	35	20.381	35	35	35	23.873	35	35
900	nonstudy	24months	35	35	35	35	35	35	35	30.009	35	35	21.907	35	24.753	35	19.478	35	26.21	35	35
901	nonstudy	12months	35	35	30.449	35	35	35	35	35	35	35	24.996	35	23.028	35	35	35	24.335	35	35
902	nonstudy	12months	35	35	35	35	35	35	35	35	35	33.565	35	35	35	35	35	35	35	35	35
903	nonstudy	0months	33.246	35	33.478	35	35	35	35	28.488	35	35	21.871	35	22	35	35	35	28.464	35	35
904	nonstudy	24months	35	35	28.676	35	35	35	35	30.782	35	32.731	26.879	35	32.458	35	31.135	35	31.443	35	35
905	placebo	24months	35	35	35	35	35	35	35	35	34.672	35	24.07	35	35	35	35	35	26.309	35	35
906	azithro	24months	35	34.212	28.5	35	35	35	35	26.462	35	35	18.669	35	25.161	35	35	35	23.502	35	35
907	nonstudy	24months	35	35	33.771	35	35	17.545	35	35	35	35	20.775	35	22.984	35	24.487	34.64	22.775	35	35
908	azithro	12months	35	35	35	35	35	35	35	31.864	35	35	24.92	35	35	35	35	35	35	35	35
909	placebo	0months	35	35	35	35	35	35	35	26.711	35	35	23.442	35	23.386	35	35	25.809	25.828	35	35
910	placebo	24months	35	16.725	35	35	35	35	35	26.168	35	35	18.784	35	22.909	35	35	35	26.436	35	35
911	nonstudy	24months	35	35	32.818	35	35	35	35	30.882	35	35	29.234	35	21.088	35	35	35	34.352	35	35
912	placebo	0months	33.156	35	30.625	35	35	35	35	35	35	35	20.512	35	19.774	35	35	35	26.166	35	35
913	placebo	12months	27.629	35	24.315	35	35	35	35	35	35	35	32.33	35	21.077	35	35	35	26.863	35	35
914	placebo	24months	35	28.875	24.251	35	35	35	35	22.121	35	35	20.365	35	25.456	35	35	35	23.11	35	35
915	nonstudy	24months	35	35	35	35	35	35	35	35	35	35	35	35	24.496	35	35	35	29.653	35	35
916	nonstudy	12months	35	35	33.234	35	35	35	35	35	35	35	32.4	35	21.067	35	35	35	26.592	35	35
917	nonstudy	24months	26.764	35	35	35	35	35	35	28.005	35	35	25.105	35	22.827	35	35	35	29.163	35	35
918	nonstudy	24months	35	35	30.254	35	35	35	35	35	35	35	17.653	34.877	25.601	35	35	35	23.233	35	35
919	placebo	0months	35	35	35	35	35	35	35	35	35	34.925	35	35	35	35	35	35	35	35	35
920	nonstudy	24months	35	35	27.289	35	35	35	35	32.336	35	35	23.496	35	28.252	35	35	35	25.184	35	35
921	placebo	12months	35	35	35	35	35	35	35	35	35	35	19.987	34.124	23.804	35	35	35	24.223	35	35
922	placebo	0months	35	35	27.593	35	35	35	35	24.107	35	35	22.269	35	26.071	35	35	35	34.212	35	35

923	nonstudy	24months	33.743	35	32.833	35	35	35	35	28.438	35	23.409	24.935	24.543	24.313	35	33.6	35	26.881	35	35
924	placebo	12months	35	35	26.182	35	35	35	35	35	35	35	25.493	35	23.524	35	35	35	27.943	35	35
925	placebo	12months	35	35	31.082	35	35	35	35	29.969	35	35	33.857	35	26.396	35	35	35	32.336	35	35
926	nonstudy	24months	35	35	34.973	35	35	35	35	30.763	35	35	21.316	35	22.535	35	35	35	26.001	35	35
927	nonstudy	0months	35	35	35	35	35	35	28.949	35	35	35	19.25	35	25.728	35	35	35	31.756	35	35
928	nonstudy	24months	35	35	28.32	35	18.015	35	35	35	35	35	35	35	24.529	35	35	35	29.66	35	35
929	placebo	12months	35	35	35	35	35	35	35	35	35	35	26.387	35	24.085	35	35	35	28.074	35	35
930	azithro	24months	35	35	25.434	35	35	35	35	35	35	35	17.253	35	16.52	35	35	35	21.484	35	35
931	azithro	24months	35	27.051	25.387	35	35	35	35	32.518	19.412	35	18.917	35	21.961	35	35	35	29.573	35	35
932	placebo	0months	35	35	35	35	35	35	35	35	35	35	35	35	35	35	35	31.329	35	35	35
933	nonstudy	12months	35	35	35	35	35	35	35	35	35	35	35	35	35	35	35	30.84	32.441	35	35
934	nonstudy	24months	35	35	35	35	35	35	28.367	35	35	35	27.265	35	21.781	35	35	35	35	35	35
935	placebo	24months	35	35	29.067	35	35	35	35	35	35	35	18.243	35	22.08	35	35	35	23.569	35	35
936	nonstudy	24months	35	35	35	35	35	35	35	35	35	35	35	35	32.339	35	35	25.971	35	35	35
937	placebo	0months	31.644	35	28.476	35	35	35	35	35	35	35	24.073	35	19.961	35	35	35	26.324	35	35
938	azithro	0months	35	35	32.061	35	35	35	35	35	35	35	18.053	35	35	35	35	35	30.878	35	35
939	azithro	12months	35	35	35	35	35	35	35	27.529	35	35	22.122	35	25.329	35	35	35	24.458	35	35
940	nonstudy	0months	35	35	27.332	35	35	35	35	25.19	35	35	20.378	35	27.182	35	32.821	35	26.497	35	35
941	placebo	24months	35	35	35	35	35	35	35	35	35	35	24.092	35	35	35	35	29.806	35	35	35
942	azithro	0months	35	34.307	25.262	35	35	35	35	26.069	35	35	22.458	35	21.904	35	20.527	35	27.002	35	35
943	placebo	12months	35	35	29.547	35	35	35	35	32.945	35	35	35	35	29.095	35	35	35	29.825	35	35
944	placebo	24months	35	35	35	35	35	35	35	35	35	35	35	35	35	35	35	27.321	35	35	35
945	nonstudy	0months	35	35	28.555	35	35	35	35	35	35	35	23.273	24.104	22.236	35	35	35	25.736	35	35
946	nonstudy	24months	35	35	30.749	35	35	35	35	29.403	35	35	20.495	20.827	24.752	35	35	35	24.114	35	35
947	placebo	24months	35	35	35	35	35	35	35	35	35	35	22.888	35	24.922	35	23.077	35	32.733	35	35
948	azithro	24months	35	35	35	35	35	35	35	35	25.646	35	35	35	35	35	32.09	33.777	24.601	35	35
949	nonstudy	24months	35	35	27.551	35	35	35	35	35	35	35	35	35	35	35	31.526	23.95	35	35	35
950	nonstudy	24months	35	35	30.547	35	35	35	35	35	19.672	35	18.655	35	20.439	35	34.98	35	20.92	35	35
951	nonstudy	24months	20.643	34.544	30.225	35	35	35	35	35	35	35	19.545	35	20.906	35	35	35	21.666	35	35
952	placebo	24months	35	35	23.661	35	35	35	35	29.281	35	35	23.35	35	23.977	35	35	35	24.345	35	35
953	nonstudy	24months	35	35	23.358	35	35	35	35	27.069	35	35	19.948	35	29.714	35	35	35	23.301	34.96	35
954	azithro	0months	35	35	35	35	35	35	35	32.753	35	35	20.043	35	23.526	35	35	35	23.571	35	35
955	azithro	12months	32.9	31.321	30.836	35	35	35	33.978	31.775	34.026	35	18.385	35	19.113	35	34.098	35	21.104	32.039	35
956	nonstudy	24months	35	35	35	35	35	24.179	35	35	35	35	25.047	35	23.287	35	21.091	35	25.478	35	35
957	azithro	0months	35	35	35	35	35	35	30.873	35	35	35	27.019	35	23.645	35	25.614	35	23.136	35	35
958	nonstudy	12months	35	35	35	35	35	35	35	35	35	35	35	35	35	35	35	35	35	35	35
959	azithro	12months	35	35	32.075	35	35	35	35	32.484	35	35	22.04	35	30.682	35	35	35	27.175	35	35
960	nonstudy	0months	15.595	35	29.549	35	35	35	35	35	35	35	23.832	35	22.264	35	35	35	22.651	35	35
961	azithro	12months	35	35	30.065	35	35	35	35	27.591	35	35	21.153	35	21.446	35	30.745	35	24.771	35	35
962	placebo	24months	35	35	22.746	35	35	35	35	35	16.599	35	19.716	19.275	34.382	35	35	35	24.392	35	35
963	azithro	0months	35	35	29.396	35	35	35	35	35	35	35	26.117	35	23.453	35	35	35	28.208	35	35
964	nonstudy	12months	35	35	31.158	35	35	35	31.665	35	35	35	21.304	35	34.361	35	35	35	27.962	35	35
965	nonstudy	12months	35	35	32.508	35	35	35	22.946	35	35	35	35	35	20.926	35	35	35	35	35	35
966	nonstudy	12months	35	35	35	35	35	35	35	35	35	35	22.931	35	25.471	35	35	35	27.931	35	31.357
967	azithro	12months	32.163	35	34.674	35	35	35	35	35	35	35	28.081	35	35	35	35	35	26.553	35	35
968	placebo	0months	35	35	35	35	35	33.963	35	35	35	30.941	33.471	35	35	35	35	35	35	35	35
969	nonstudy	24months	35	35	35	35	35	35	35	35	35	35	23.652	35	35	35	35	35	24.939	35	35
970	nonstudy	24months	35	35	35	35	35	35	32.502	35	35	35	23.591	35	23.853	35	35	35	26.473	35	35
971	azithro	0months	35	35	33.024	35	21.718	35	35	35	35	35	18.726	35	35	35	35	35	35	35	35
972	placebo	12months	35	35	31.733	35	35	35	35	35	35	35	26.975	35	24.833	35	35	35	27.384	35	35
973	azithro	24months	35	30.814	30.566	35	35	35	35	35	35	35	18.747	35	22.773	35	35	35	19.611	35	35
974	nonstudy	0months	35	35	30.155	35	35	18.061	35	35	35	35	20.294	35	22.767	35	35	35	29.588	35	35
975	azithro	24months	35	35	23.753	35	35	35	35	31.467	35	35	19.522	35	21.099	35	35	35	22.964	35	35
976	azithro	0months	32.501	35	35	35	35	35	35	35	35	35	23.62	35	22.906	35	35	35	25.032	35	35
977	nonstudy	24months	35	35	35	35	35	35	35	27.411	35	35	18.785	35	19.91	35	35	35	24.337	35	35
978	nonstudy	0months	35	34.282	26.855	35	35	35	34.844	35	30.953	35	29.122	35	32.241	35	19.974	35	32.981	35	35
979	nonstudy	0months	35	35	25.828	35	35	35	35	35	35	35	20.98	35	20.888	35	35	35	23.993	35	35
980	nonstudy	24months	35	35	31.175	35	35	35	35	29.451	27.446	35	19.964	35	23.792	35	34.432	32.522	18.548	35	35
981	placebo	0months	23.033	23.204	28.157	35	35	35	35	35	35	32.366	27.972	35	33.299	35	35	35	35	35	35
982	nonstudy	0months	35	35	35	35	35	35	35	35	35	35	35	35	35	35	35	35	35	35	35
983	azithro	0months	33.065	27.915	35	16.939	35	35	35	35	35	35	26.102	35	35	35	35	35	24.718	35	35
984	placebo	24months	34.821	35	35	35	35	35	35	35	35	34.918	28.179	35	19.896	35	35	35	35	33.444	35
985	azithro	24months	35	35	35	35	35	35	35	35	35	35	35	35	33.416	35	35	27.742	35	35	35
986	azithro	0months	35	31.863	35	35	35	35	35	25.773	35	35	22.398	35	28.125	35	35	35	27.744	35	35
987	placebo	24months	35	35	31.274	35	35	35	35	35	21.36	35	25.031	35	35	35	35	35	25.031	35	35
988	placebo	24months	32.045	35	35	35	35	35	35	35	35	35	27.801	35	20.899	35	35	32.079	35	35	35

989	nonstudy	0months	14.635	32.713	27.417	35	35	35	25.154	35	35	35	26.797	35	19.943	35	28.882	35	25.841	35	35
990	azithro	0months	31.324	35	28.358	35	35	35	35	35	35	35	20.084	35	20.97	35	27.635	35	24.968	35	35
991	placebo	24months	35	35	31.945	35	35	35	35	28.262	35	35	18.635	35	21.348	35	34.459	35	22.692	35	35
992	nonstudy	24months	35	35	35	35	35	35	35	22.145	35	35	22.651	35	22.267	35	35	35	26.694	35	35
993	placebo	24months	35	35	35	35	35	35	35	35	35	35	21.27	35	21.72	35	35	35	25.818	35	35
994	nonstudy	24months	19.955	35	27.332	35	35	35	35	35	35	34.093	32.512	35	21.581	35	35	35	25.107	35	35
995	placebo	0months	35	35	35	35	35	35	35	30.791	35	35	22.75	35	21.104	35	35	35	24.955	35	35
996	placebo	12months	35	35	35	35	35	35	35	27.609	35	35	25.742	35	21.237	35	35	28.703	26.308	35	35
997	azithro	0months	33.868	35	27.205	35	35	35	35	32.246	35	35	35	35	22.097	35	35	35	24.843	35	35
998	nonstudy	0months	31.043	35	25.043	35	35	35	35	35	35	35	26.838	35	23.567	35	35	35	24.638	35	35
999	nonstudy	24months	35	35	31.185	35	35	35	35	35	19.752	35	24.445	35	23.217	35	35	35	24.928	35	35
1000	nonstudy	24months	35	35	28.866	35	35	18.234	35	35	35	35	22.045	35	35	35	35	35	27.172	35	35
1001	placebo	24months	35	35	35	35	35	19.669	35	35	34.396	35	23.611	35	21.384	35	35	33.525	23.309	35	35
1002	nonstudy	0months	17.665	35	35	35	35	35	35	35	35	35	35	35	22.83	35	35	35	31.302	35	35
1003	azithro	0months	35	25.444	30.486	35	35	35	21.168	32.397	32.878	35	25.317	35	24.353	35	35	35	25.184	35	35
1004	placebo	24months	34.218	35	35	35	35	35	35	35	35	31.188	23.768	35	27.869	35	35	35	29.601	35	35
1005	nonstudy	12months	35	35	35	35	35	35	35	35	35	35	35	35	31.904	35	35	35	33.359	35	35
1006	azithro	24months	35	35	35	35	35	35	35	35	35	35	22.236	35	26.613	35	35	35	27.123	35	35
1007	nonstudy	24months	35	28.923	27.455	35	35	35	35	35	35	35	26.519	35	24.414	35	35	35	24.598	35	35
1008	placebo	24months	35	35	27.016	35	35	35	35	31.033	35	35	22.897	35	20.626	35	34.205	35	23.604	35	35
1009	placebo	24months	31.907	35	35	35	35	35	35	34.905	35	33.881	35	35	22.164	35	35	35	35	35	35
1010	placebo	24months	35	35	34.086	35	35	35	35	35	35	35	35	35	35	35	35	30.601	35	35	35
1011	nonstudy	0months	32.156	35	34.143	35	35	35	35	35	35	35	24.008	35	22.359	35	35	35	27.26	35	35
1012	placebo	12months	18.005	35	32.3	35	35	35	35	31.209	35	35	35	35	23.785	35	35	35	35	35	35
1013	azithro	0months	35	35	30.523	35	35	35	33.753	29.935	35	35	18.998	35	22.174	35	25.919	33.314	24.407	35	35
1014	nonstudy	12months	35	35	35	35	35	35	35	35	35	35	30.328	35	24.39	35	35	35	30.448	35	35
1015	azithro	12months	35	35	35	35	35	35	35	25.122	35	35	35	35	35	35	35	33.721	24.132	35	35
1016	placebo	0months	35	35	25.538	35	35	35	35	35	35	35	25.109	35	20.815	35	32.613	35	26.895	35	35
1017	placebo	0months	35	35	35	35	35	35	35	35	35	33.646	34.404	35	35	35	35	35	31.426	35	35
1018	nonstudy	24months	35	35	35	35	35	35	35	35	35	35	28.307	35	27.317	35	28.524	35	29.209	35	35
1019	nonstudy	24months	35	35	31.49	35	35	35	35	35	35	35	26.082	35	35	35	35	35	35	35	35
1020	azithro	0months	35	35	35	35	35	30.907	35	35	35	31.924	35	35	35	35	35	35	35	35	35
1021	azithro	0months	35	35	35	35	35	35	35	35	35	35	26.311	35	23.343	35	35	35	27.311	35	35
1022	nonstudy	12months	35	35	35	35	35	35	35	35	35	35	35	35	32.892	35	35	35	29.876	35	35
1023	nonstudy	24months	35	35	31.568	35	35	35	35	35	35	35	22.458	35	21.862	35	35	33.618	19.4	35	35
1024	placebo	12months	20.477	34.28	28.526	35	35	35	35	29.758	35	35	17.521	35	20.024	35	35	35	21.844	35	35
1025	azithro	0months	35	35	26.46	35	35	35	35	30.406	35	35	20.59	35	23.986	35	35	35	23.587	35	35
1026	azithro	24months	35	35	33.365	35	35	35	20.035	35	32.216	35	21.684	35	21.633	35	35	35	23.573	35	35
1027	nonstudy	24months	35	35	26.09	35	35	35	35	28.653	35	35	17.382	35	23.209	35	35	35	26.311	35	35
1028	placebo	0months	27.773	35	28.689	35	35	35	35	26.075	35	35	19.178	35	26.21	35	35	35	25.891	35	35
1029	nonstudy	24months	35	35	26.839	35	35	35	35	35	35	35	25.88	25.837	20.34	35	29.306	35	24.592	35	35
1030	nonstudy	12months	35	35	28.399	35	14.022	35	35	33.069	35	35	21.691	35	20.369	35	35	35	24.056	35	35
1031	nonstudy	24months	35	35	29.014	35	34.286	35	35	24.138	35	35	20.119	35	23.559	35	35	35	23.151	35	35
1032	azithro	0months	35	35	21.046	35	35	35	35	31.744	35	35	22.769	35	19.55	35	35	35	22.228	35	35
1033	placebo	0months	35	35	35	35	35	35	35	24.164	35	35	17.791	35	21.804	35	26.472	35	20.108	35	35
1034	azithro	12months	35	32.957	27.821	35	35	35	35	35	35	35	24.014	35	35	35	35	35	26.136	35	35
1035	nonstudy	12months	35	35	28.128	35	35	35	35	25.237	35	35	34.644	35	20.604	35	35	35	24.792	35	35
1036	nonstudy	0months	28.511	35	24.972	35	35	35	35	28.886	26.249	35	19.472	35	19.946	35	35	35	23.036	33.998	35
1037	azithro	12months	31.364	35	35	35	35	35	35	32.802	35	35	19.616	35	19.021	35	35	35	28.965	35	35
1038	azithro	24months	34.487	34.516	25.319	35	35	35	35	31.716	35	35	18.466	35	19.481	35	35	35	22.493	35	35
1039	nonstudy	24months	35	35	20.764	35	35	35	35	27.42	35	35	22.93	35	23.623	35	35	35	25.372	35	33.893
1040	nonstudy	0months	33.813	35	31.721	35	35	35	35	23.834	35	35	19.221	35	29.715	35	35	35	28.211	35	35
1041	nonstudy	12months	35	35	30.599	35	35	35	33.729	35	35	35	18.631	35	35	35	35	35	25.489	35	35
1042	nonstudy	0months	35	35	30.094	35	35	35	35	35	35	35	15.895	35	27.366	35	35	35	34.633	35	35
1043	placebo	0months	32.982	35	25.66	35	35	35	35	31.231	35	35	23.556	35	23.717	35	35	35	25.245	35	35
1044	azithro	24months	35	35	34.908	35	35	35	35	35	35	35	35	35	18.936	35	35	33.278	26.231	35	35
1045	nonstudy	0months	35	35	35	35	35	35	35	35	35	35	19.502	35	19.946	35	35	35	27.457	35	35
1046	nonstudy	0months	35	35	28.142	29.103	35	35	35	35	20.809	35	19.666	35	22.977	35	35	35	23.59	35	35
1047	nonstudy	0months	35	35	26.241	35	35	35	32.969	27.677	35	35	35	35	24.173	35	35	35	22.603	35	35
1048	placebo	0months	35	35	35	35	35	30.512	35	35	35	35	35	35	35	35	35	35	35	35	35
1049	azithro	24months	25.331	35	26.93	35	35	35	35	33.832	35	35	21.597	35	26.236	35	35	35	23.577	35	35
1050	azithro	24months	35	35	29.271	35	35	35	35	33.941	35	35	28.604	35	22.7	35	35	35	25.655	35	35
1051	nonstudy	12months	35	35	35	35	35	35	35	35	35	35	35	35	31.815	35	35	33.548	32.99	35	35
1052	azithro	24months	35	35	29.284	35	35	35	20.129	35	20.121	35	18.191	35	21.379	35	35	35	21.044	35	35
1053	azithro	12months	35	35	30.102	35	35	35	33.654	34.089	35	35	27.709	35	24.261	35	35	35	27.607	35	35
1054	placebo	0months	31.668	35	28.746	35	35	35	35	19.063	35	35	19.562	35	25.025	35	35	35	24.317	35	35

1055	azithro	0months	32.638	35	30.15	35	35	35	35	35	35	35	17.969	35	28.193	35	35	35	22.645	35	35
1056	azithro	0months	35	33.936	30.525	35	20.89	35	35	35	35	35	18.785	18.292	20.537	35	35	35	22.036	35	35
1057	placebo	24months	35	35	35	35	35	35	35	35	35	32.821	19.209	35	35	35	31.12	29.511	34.15	35	35
1058	placebo	0months	35	35	35	35	35	35	35	35	35	35	23.124	35	20.552	35	35	35	22.426	35	35
1059	placebo	0months	35	35	35	35	35	35	35	21.696	35	35	25.525	35	24.768	35	35	35	27.658	35	35
1060	nonstudy	0months	35	35	35	35	35	35	35	35	35	35	26.177	35	21.69	35	35	35	35	35	35
1061	azithro	0months	35	35	26.246	35	35	35	35	35	35	35	24.192	35	21.07	35	35	35	27.878	35	35
1062	azithro	24months	35	35	35	35	35	35	35	35	35	35	18.679	35	19.509	35	35	35	28.678	35	35
1063	azithro	24months	35	35	29.154	35	35	35	32.655	35	35	35	21.751	35	19.175	35	35	35	24.932	35	35
1064	nonstudy	0months	22.381	35	29.203	35	35	35	35	35	35	35	16.95	35	20.508	35	35	35	21.251	35	35
1065	nonstudy	24months	35	35	24.88	35	35	35	35	33.316	35	35	20.597	35	20.031	35	35	34.47	23.541	35	35
1066	nonstudy	24months	35	35	35	35	35	35	35	35	35	35	33.768	35	35	35	35	27.364	35	35	35
1067	azithro	0months	35	35	28.095	35	35	35	30.487	35	35	35	22.782	35	25.221	35	35	32.489	25.072	35	35
1068	nonstudy	12months	35	35	35	35	35	35	35	21.86	35	35	20.65	35	23.115	35	35	35	25.042	35	35
1069	nonstudy	24months	35	35	27.933	35	35	35	35	31.712	35	35	29.401	35	30.611	35	35	32.474	27.308	32.777	35
1070	azithro	12months	35	35	29.316	35	35	35	35	35	35	35	21.482	35	22.442	35	35	35	23.883	35	35
1071	nonstudy	24months	35	35	23.515	35	35	35	35	35	35	35	33.669	35	21.993	35	35	35	22.734	35	35
1072	placebo	24months	35	35	24.079	35	35	35	35	35	35	35	19.125	35	22.735	35	35	35	22.999	35	35
1073	azithro	24months	35	35	34.587	35	35	35	35	28.288	35	35	19.701	35	21.128	35	35	33.752	22.332	35	35
1074	azithro	0months	35	35	35	35	35	33.838	35	35	35	35	22.34	35	21.729	35	26.48	35	23.617	35	35
1075	nonstudy	24months	35	35	25.932	35	35	35	35	32.24	35	35	19.826	35	21.09	35	35	35	25.145	35	35
1076	nonstudy	12months	35	22.441	33.111	35	35	35	32.872	35	35	35	18.872	35	20.408	35	35	35	34.181	35	35
1077	placebo	0months	35	35	34.948	35	35	35	35	29.23	35	35	24.664	35	24.939	35	35	35	27.923	35	35
1078	placebo	0months	35	35	28.8	35	35	35	35	35	35	35	21.959	35	21.904	35	35	35	25.962	35	35
1079	nonstudy	24months	34.046	35	24.529	35	35	35	35	33.121	35	35	24.608	35	21.623	35	35	35	25.026	35	35
1080	placebo	12months	35	35	29.553	35	35	35	35	24.937	35	35	26.817	35	24.26	35	35	35	26.34	35	35
1081	nonstudy	12months	35	22.55	25.99	35	35	35	35	35	35	35	23.015	35	24.831	35	35	35	26.616	35	35
1082	nonstudy	0months	35	35	35	35	35	35	35	27.591	35	35	35	35	20.888	35	35	35	21.696	35	35
1083	azithro	24months	35	35	32.056	35	35	35	35	34.228	35	35	21.177	35	24.287	35	35	35	23.653	35	35
1084	nonstudy	24months	30.849	35	35	35	35	35	35	18.714	35	35	21.081	35	20.704	35	35	27.284	27.256	35	35
1085	nonstudy	12months	35	35	35	35	35	35	35	35	35	35	35	35	35	35	35	35	35	35	35
1086	placebo	0months	32.967	35	35	35	35	35	18.84	35	35	34.808	34.589	35	25.399	35	35	35	34.38	35	35
1087	azithro	0months	35	35	35	35	35	35	35	35	35	34.162	25.235	35	26.993	35	35	35	26.381	35	35
1088	placebo	0months	35	35	32.872	35	35	14.423	35	35	35	29.791	27.058	35	19.95	35	35	35	22.074	35	35
1089	placebo	0months	35	35	35	35	35	35	35	35	35	35	19.037	35	20.945	35	35	35	35	35	35
1090	nonstudy	24months	35	35	35	35	35	35	35	25.957	35	35	28.692	35	30.179	35	35	35	29.287	35	35
1091	nonstudy	0months	35	35	28.229	35	35	35	35	35	35	35	27.344	35	20.675	35	35	35	26.094	35	35
1092	placebo	0months	35	35	35	35	35	35	30.144	35	32.252	33.906	22.32	35	25.495	35	35	35	30.741	35	35
1093	azithro	12months	27.533	35	30.251	35	35	35	35	29.186	35	35	27.455	35	21.879	35	35	35	32.447	35	35
1094	placebo	12months	34.04	35	28.2	35	35	35	35	26.331	35	35	25.023	35	25.027	35	35	35	24.508	35	35
1095	nonstudy	12months	35	35	35	35	35	35	35	35	35	35	33.521	35	19.762	35	35	35	31.788	35	35
1096	azithro	24months	35	35	27.701	35	35	35	35	35	35	35	23.957	35	22.829	35	35	35	27.671	35	35
1097	nonstudy	24months	35	35	27.353	35	35	35	35	26.579	35	35	18.079	35	22.572	35	35	35	21.576	35	35
1098	placebo	12months	35	35	35	35	35	35	35	19.208	35	34.996	35	35	27.065	35	35	35	31.614	35	35
1099	azithro	0months	35	35	35	35	35	35	35	35	35	35	21.554	20.758	22.334	35	35	35	28.645	35	35
1100	placebo	0months	35	35	27.228	35	35	35	35	25.615	35	35	23.729	35	24.712	35	35	35	27.974	35	35
1101	nonstudy	0months	35	25.713	27.772	35	35	35	35	32.592	35	35	24.854	35	22.479	35	35	35	24.859	35	35
1102	azithro	0months	35	35	27.553	35	35	35	35	35	35	29.998	22.838	35	23.777	35	35	35	22.923	35	35
1103	azithro	24months	35	35	35	35	35	35	35	35	35	35	35	35	20.93	35	35	35	28.898	35	35
1104	nonstudy	24months	35	35	23.388	35	35	35	35	35	35	34.88	23.783	35	19.748	35	35	35	26.547	35	35
1105	nonstudy	0months	35	35	35	35	35	35	35	35	35	35	20.037	35	35	35	35	35	20.855	35	35
1106	nonstudy	12months	35	35	24.384	35	35	35	35	24.338	35	35	35	35	20.787	35	35	35	22.741	35	35
1107	nonstudy	24months	35	35	34.119	35	35	35	35	35	35	35	20.623	35	20.424	35	35	35	23.971	35	35
1108	nonstudy	12months	35	35	29.538	35	35	35	35	33.314	35	35	25.393	35	22.792	35	35	35	25.408	35	35
1109	azithro	24months	35	35	26.865	35	35	35	35	35	35	35	28.115	35	22.966	35	35	35	29.365	35	35
1110	placebo	0months	35	35	35	35	35	35	35	24.657	35	35	19.52	35	22.37	35	35	35	23.138	35	35
1111	placebo	24months	35	35	31.217	35	35	35	35	35	35	35	22.437	35	19.669	35	35	35	31.491	35	35
1112	nonstudy	24months	35	35	29.375	35	35	35	35	35	19.213	35	20.005	35	22.428	35	35	35	24.147	35	35
1113	azithro	12months	35	35	35	35	35	35	35	30.085	35	35	22.968	35	24.877	35	35	35	29.002	35	35
1114	nonstudy	24months	35	35	35	35	35	35	35	35	35	35	35	35	35	35	35	27.965	35	35	35
1115	nonstudy	24months	35	35	24.49	35	35	35	35	21.145	35	34.439	20.936	35	22.235	35	35	35	25.721	35	35
1116	placebo	12months	35	35	29.378	35	35	35	35	35	35	35	22.824	35	26.36	35	35	35	23.407	35	35
1117	azithro	24months	35	35	27.072	35	35	35	35	29.58	35	35	20.09	35	22.416	35	35	35	24.188	34.803	35
1118	azithro	0months	35	35	35	35	35	35	35	35	34.571	35	31.134	35	19.573	35	35	35	26.698	35	35
1119	placebo	0months	35	35	35	35	35	33.679	35	35	35	32.969	35	35	35	35	35	35	35	35	35
1120	placebo	0months	35	35	34.03	35	35	35	35	28.948	35	9.762	19.44	35	22.065	35	35	27.478	23.035	35	35

1121	nonstudy	0months	35	35	28.553	35	35	35	24.453	35	35	35	19.616	35	21.112	35	35	35	21.347	35	35
1122	placebo	0months	35	35	35	35	21.15	35	35	35	35	33.579	25.749	35	26.7	35	35	35	22.718	35	35
1123	nonstudy	0months	32.095	35	26.381	35	35	35	35	35	35	30.612	26.809	35	21.502	35	35	34.455	26.951	35	35
1124	azithro	0months	33.017	35	28.489	35	35	35	32.269	25.55	35	35	24.974	35	24.871	35	35	35	25.79	35	35
1125	nonstudy	24months	35	35	35	35	35	35	20.897	35	35	35	21.488	35	22.248	35	35	35	23.302	35	35
1126	placebo	24months	35	35	29.997	35	35	35	35	35	16.933	35	27.37	35	22.612	35	35	35	29.297	35	35
1127	azithro	12months	21.319	35	35	35	35	35	35	35	35	35	35	35	35	35	35	35	35	35	34.084
1128	placebo	0months	26.728	35	35	35	35	35	35	35	35	35	15.507	35	20.145	35	35	35	22.88	35	35
1129	nonstudy	0months	35	35	35	35	35	35	35	35	35	35	18.583	35	19.245	35	35	35	23.26	35	35
1130	nonstudy	0months	35	35	28.416	35	35	35	35	35	35	35	20.186	35	20.014	35	35	34.716	24.039	35	35
1131	nonstudy	0months	35	32.662	25.25	33.879	24.239	35	35	35	33.215	35	30.905	35	22.921	35	35	35	25.364	33.676	35
1132	azithro	24months	35	35	31.352	35	35	35	35	35	35	35	35	35	35	35	35	31.902	35	35	35
1133	nonstudy	24months	35	29.307	24.101	35	35	35	35	35	33.377	35	23.293	35	35	35	35	35	25.728	35	35
1134	nonstudy	0months	35	27.35	33.123	35	35	35	35	35	35	35	19.549	35	26.824	35	35	35	23.024	35	35
1135	nonstudy	24months	35	35	25.083	35	35	35	35	35	32.185	35	19.77	35	25.184	35	35	35	23.676	35	35
1136	nonstudy	24months	35	35	23.393	35	35	35	35	21.098	35	35	25.752	35	22.972	35	30.586	35	35	35	35
1137	azithro	0months	31.234	33.12	26.039	35	35	35	33.07	28.548	35	35	23.767	35	21.952	35	35	35	27.93	35	35
1138	placebo	24months	35	35	35	35	35	35	35	35	35	34.376	35	35	22.835	35	35	29.397	27.78	35	35
1139	placebo	0months	33.78	35	32.546	35	35	35	35	28.934	35	35	21.699	35	23.691	35	35	35	25.127	35	35
1140	azithro	0months	27.628	35	35	35	35	35	22.181	35	35	35	19.944	35	25.933	35	35	35	23.934	35	35
1141	nonstudy	0months	35	35	35	35	35	35	35	24.322	35	35	18.846	35	18.386	35	35	35	20.584	35	35
1142	nonstudy	24months	35	35	35	35	35	35	35	35	35	35	35	35	31.622	35	35	35	35	35	35
1143	placebo	0months	35	35	29.244	35	35	35	23.508	35	35	35	22.972	35	25.369	35	35	35	23.966	35	35
1144	azithro	24months	35	34.284	35	35	35	35	35	24.459	35	35	35	35	35	35	35	35	35	35	34.134
1145	placebo	12months	35	34.122	35	35	35	35	35	31.397	35	33.236	32.563	35	25.139	35	35	35	28.776	35	35
1146	placebo	0months	33.079	35	25.349	35	35	35	35	31.719	35	35	23.525	35	23.118	35	35	35	26.371	35	35
1147	nonstudy	0months	35	35	25.085	35	35	35	26.917	35	35	35	21.836	35	19.633	35	35	35	22.98	35	35
1148	azithro	12months	25.69	35	32.304	35	35	35	35	25.785	35	35	21.535	35	22.816	35	35	35	25.752	35	35
1149	placebo	0months	35	35	35	35	35	35	33.009	32.514	35	35	22.314	35	25.976	35	35	35	24.53	35	35
1150	placebo	24months	35	35	34.243	35	35	35	35	35	35	35	32.891	35	18.408	35	35	35	24.752	35	35
1151	nonstudy	24months	30.078	35	35	35	35	35	27.676	35	35	35	22.485	35	23.915	35	35	35	23.833	35	35
1152	azithro	12months	35	31.776	29.216	35	35	35	35	35	35	35	18.471	35	17.979	35	35	35	21.722	35	35
1153	nonstudy	24months	35	35	33.852	35	35	35	34.908	35	35	34.611	18.842	35	26.086	35	35	35	25.688	35	35
1154	nonstudy	24months	35	35	35	35	35	35	35	35	35	35	35	35	35	35	35	28.561	35	35	35
1155	nonstudy	24months	35	35	35	35	35	35	35	34.211	35	35	35	35	30.421	35	35	35	22.882	35	35
1156	azithro	12months	30.377	35	24.297	35	35	35	35	35	35	35	25.984	35	35	35	35	32.925	23.617	35	35
1157	nonstudy	12months	35	35	35	35	35	35	35	35	35	35	20.003	35	19.855	35	35	32.755	25.728	35	32.919
1158	azithro	24months	35	35	23.406	35	35	35	35	35	35	35	22.046	35	20.926	35	35	35	28.421	35	35
1159	azithro	12months	34.394	35	24.137	35	35	35	35	25.843	35	35	21.334	35	22.161	35	35	35	23.359	35	35
1160	azithro	12months	34.416	35	30.5	35	35	35	35	29.736	35	35	23.587	35	28.478	35	35	35	35	35	35
1161	nonstudy	0months	27.891	35	28.428	35	35	35	28.707	35	35	35	22	35	20.267	35	35	35	23.855	35	35
1162	nonstudy	0months	35	35	35	23.995	35	35	35	35	35	35	27.057	35	19.546	35	35	33.592	22.873	35	35
1163	nonstudy	0months	13.667	35	35	35	35	35	34.288	32.386	35	35	22.535	35	24.883	35	35	35	26.127	35	35
1164	placebo	0months	35	34.107	29.936	35	35	35	35	35	17.486	35	18.499	35	25.578	35	35	35	20.704	35	35
1165	placebo	24months	35	35	28.939	35	35	35	35	35	35	35	21.323	35	22.543	35	35	35	27.64	35	35
1166	azithro	24months	35	35	35	35	35	35	35	26.538	35	35	20.585	35	24.405	35	35	35	35	35	35
1167	placebo	0months	33.328	35	33.604	35	35	35	35	35	35	35	25.471	35	22.425	35	35	35	24.98	35	35
1168	placebo	0months	35	35	35	35	35	17.063	35	35	35	35	26.176	35	22.025	35	35	35	27.27	35	35
1169	placebo	0months	35	29.691	30.126	35	35	35	35	35	35	35	22.948	35	22.789	35	35	35	35	35	35
1170	nonstudy	0months	33.764	35	35	35	35	35	35	32.092	35	35	25.06	35	31.444	35	35	35	26.564	35	35
1171	nonstudy	24months	35	29.178	28.202	35	35	35	35	26.851	35	35	21.327	35	22.71	35	35	35	22.848	35	35
1172	azithro	0months	35	35	35	35	35	35	35	35	35	35	35	35	21.794	35	31.542	33.594	35	35	35
1173	nonstudy	12months	35	35	35	35	35	35	35	25.837	35	35	35	35	21.973	35	31.929	35	35	35	35
1174	azithro	12months	35	35	31.509	35	35	35	35	35	35	35	22.835	35	21.775	35	33.258	35	27.116	35	35
1175	nonstudy	24months	35	35	23.766	35	35	35	35	30.689	35	35	22.242	35	21.662	35	35	35	23.427	35	35
1176	azithro	0months	26.949	35	35	35	35	35	35	35	35	35	31.992	35	26.822	35	35	35	35	35	35
1177	nonstudy	24months	35	35	35	35	35	35	35	25.068	35	35	21.233	35	35	35	35	35	26.03	35	35
1178	placebo	0months	35	35	31.618	35	35	35	35	26.024	35	29.488	31.736	35	30.238	35	35	35	31.346	35	35
1179	azithro	12months	32.876	35	29.882	35	35	35	26.509	30.697	35	35	29.758	35	21.269	35	32.264	35	25.233	31.47	35
1180	azithro	0months	35	35	35	35	35	35	35	35	35	35	35	35	23.841	35	35	35	35	21.178	35
1181	placebo	0months	33.1	35	27.938	35	35	35	35	35	35	35	16.768	35	20.183	35	35	35	23.366	35	35
1182	placebo	12months	35	35	35	35	35	35	35	35	35	33.612	35	35	31.661	35	35	35	35	35	35
1183	placebo	0months	35	35	30.201	35	35	35	31.526	19.16	35	35	23.58	35	19.177	35	35	35	33.433	35	35
1184	nonstudy	24months	34.363	35	35	35	35	35	35	23.208	35	35	21.883	35	22.88	35	35	35	26.764	35	35
1185	azithro	12months	35	35	29.387	35	35	35	35	27.78	35	35	21.026	35	25.214	35	35	35	29.443	35	35
1186	nonstudy	24months	35	32.697	29.801	35	35	35	35	28.724	35	34.532	29.121	35	21.574	35	35	35	31.381	32.867	35

1187	nonstudy	12months	35	35	35	35	35	35	35	26.177	35	35	35	35	35	35	35	35	25.801	35	35
1188	nonstudy	0months	35	35	35	35	35	35	26.441	35	35	35	24.047	35	22.891	35	35	35	24.297	33.387	35
1189	nonstudy	24months	35	35	35	35	35	35	35	35	35	35	35	35	35	35	35	27.098	35	35	35
1190	nonstudy	12months	35	35	34.901	35	35	35	35	27.708	35	35	21.799	35	23.041	35	35	35	26.907	35	35
1191	nonstudy	0months	35	35	29.776	35	35	35	30.47	35	35	35	21.494	35	25.869	35	35	35	28.081	35	35
1192	placebo	0months	34.388	35	25.21	35	35	35	35	29.417	35	35	19.387	35	18.935	35	22.803	35	21.142	35	35
1193	placebo	24months	23.166	35	35	35	35	35	21.381	35	35	35	19.834	35	21.418	35	35	35	35	35	35
1194	azithro	12months	35	35	29.999	35	35	35	35	32.591	35	35	23.931	35	22.158	35	28.375	35	24.697	35	35
1195	nonstudy	0months	29.862	35	35	35	35	35	35	35	35	35	18.765	24.197	18.997	35	35	35	27.136	35	35
1196	azithro	12months	35	35	35	35	35	35	35	30.199	35	35	19.718	35	22.347	35	35	35	27.368	35	35
1197	azithro	12months	30.976	35	35	35	35	35	35	26.256	35	35	26.505	35	34.208	35	35	35	35	35	35
1198	placebo	0months	29.753	35	29.841	35	18.265	35	35	35	35	35	29.05	35	25.94	35	35	35	19.768	35	35
1199	placebo	12months	35	35	32.351	35	35	35	35	35	35	35	35	35	35	35	35	35	35	35	35
1200	nonstudy	0months	35	35	26.832	35	35	35	35	35	35	35	28.295	35	23.046	35	35	35	25.473	35	35
1201	placebo	12months	35	35	35	35	35	35	35	33.754	35	35	29.29	35	28.153	35	35	35	27.96	35	35
1202	azithro	12months	35	35	35	35	35	35	35	31.518	35	35	22.941	35	19.893	35	35	35	27.263	35	35
1203	azithro	24months	30.718	35	35	35	35	35	35	35	35	35	35	35	35	35	35	35	29.01	35	35
1204	nonstudy	24months	35	35	35	35	35	35	35	18.305	35	35	35	35	35	35	35	35	23.974	35	35
1205	nonstudy	0months	35	35	33.441	35	35	35	35	19.14	35	35	23.452	35	20.514	35	34.946	35	22.731	35	35
1206	placebo	0months	35	35	35	35	35	35	35	29.344	35	32.709	16.581	35	22.799	35	35	35	19.705	35	35
1207	nonstudy	0months	35	35	35	35	35	35	34.448	26.806	35	35	20.36	35	22.813	35	35	35	26.146	31.82	35
1208	placebo	12months	35	35	35	35	35	35	32.981	35	35	35	23.734	35	21.367	35	35	35	26.374	35	35
1209	azithro	0months	35	35	35	35	35	35	35	35	35	29.786	35	35	35	35	35	35	35	35	35
1210	nonstudy	24months	35	35	31.364	35	35	35	35	31.514	35	35	20.068	35	18.915	35	21.894	35	23.303	35	35
1211	placebo	12months	28.189	35	34.14	27.602	35	35	35	35	35	35	15.752	35	16.958	35	35	35	20.96	33.786	35
1212	nonstudy	12months	35	35	35	35	35	35	35	33.201	35	35	31.377	33.731	31.394	35	35	35	30.942	35	35
1213	nonstudy	0months	35	35	28.839	35	35	35	35	27.519	35	35	21.372	35	24.749	35	35	35	25.174	28.856	35
1214	nonstudy	0months	32.31	35	31.265	35	35	35	35	27.813	35	35	18.279	35	20.907	35	35	35	22.289	35	35
1215	placebo	24months	34.459	35	35	35	35	35	21.469	35	35	35	23.478	35	23.014	35	35	35	24.774	35	35
1216	azithro	12months	35	35	27.925	35	35	35	24.037	30.832	35	35	30.145	35	35	35	35	29.505	35	35	35
1217	azithro	24months	33.859	35	27.987	35	35	35	35	35	35	35	20.831	35	22.785	35	35	NA	35	35	35
1218	placebo	0months	33.117	35	26.631	35	35	35	35	20.937	35	35	26.451	35	23.642	35	35	35	25.415	35	35
1219	nonstudy	24months	20.125	32.522	33.923	35	35	35	30.917	33.612	35	35	22.304	35	29.832	35	35	35	22.423	35	35
1220	azithro	12months	35	35	35	35	35	35	35	35	35	32.463	35	35	35	35	35	35	35	35	35
1221	nonstudy	0months	35	35	31.618	35	35	35	35	27.067	35	34.917	26.704	35	35	35	35	35	30.536	35	35
1222	placebo	0months	35	35	21.623	35	35	35	31.897	35	35	35	20.92	35	26.261	35	35	35	26.429	35	35
1223	nonstudy	12months	35	35	27.649	35	35	35	35	27.989	35	35	21.458	35	24.305	35	35	35	26.468	35	35
1224	nonstudy	12months	35	35	23.514	35	35	35	35	29.68	35	35	21.926	35	19.779	35	35	35	24.957	35	35
1225	azithro	0months	35	35	24.782	22.147	35	35	35	35	35	35	18.175	35	18.124	35	35	35	20.616	35	35
1226	nonstudy	0months	35	35	28.513	35	35	35	35	35	35	35	21.498	35	21.878	35	35	35	27.951	35	35
1227	nonstudy	24months	32.394	35	26.551	35	35	35	35	35	35	35	20.256	35	21.605	35	35	35	23.253	35	35
1228	azithro	12months	35	35	35	35	35	35	35	33.444	35	35	25.453	27.962	30.287	35	35	35	30.158	35	35
1229	nonstudy	12months	34.803	35	24.669	35	35	35	35	28.677	35	35	21.142	35	25.336	35	35	35	24.624	35	35
1230	placebo	24months	32.603	35	29.544	35	35	35	34.09	35	35	35	20.588	35	25.145	35	35	35	21.974	35	35
1231	nonstudy	24months	35	35	35	35	35	35	35	22.644	35	35	20.828	35	22.682	35	35	35	25.574	35	35
1232	placebo	0months	29.906	35	31.22	35	35	35	35	27.661	28.079	35	19.238	35	24.748	35	35	35	21.57	35	35
1233	placebo	24months	23.125	35	34.01	35	35	35	35	35	35	35	21.865	35	21.607	35	35	35	35	35	35
1234	azithro	0months	35	35	29.966	35	35	35	35	35	35	35	22.756	35	23.709	35	35	35	26.122	35	35
1235	azithro	0months	35	35	35	35	35	29.317	35	35	35	29.928	35	35	35	35	35	35	35	35	35
1236	placebo	24months	35	35	35	35	35	35	35	20.336	35	35	22.219	35	21.669	35	35	35	26.622	35	35
1237	nonstudy	24months	35	35	28.946	35	35	35	35	27.173	35	35	20.926	35	23.79	35	35	35	23.355	30.93	35
1238	nonstudy	12months	35	35	26.954	35	35	35	34.726	27.028	35	35	20.375	35	35	35	35	35	23.431	35	35
1239	placebo	12months	35	35	27.713	35	35	35	35	20.375	35	35	20.155	35	22.165	35	35	35	26.305	35	35
1240	placebo	0months	35	35	24.337	35	35	35	35	35	35	10.767	18.302	35	19.194	35	32.852	35	22.652	35	28.855
1241	azithro	0months	28.868	35	26.301	35	35	35	35	30.561	35	35	18.1	35	27.386	35	35	35	32.685	35	35
1242	azithro	24months	35	35	27.574	35	35	35	35	35	35	35	20.712	35	26.91	35	31.811	35	23.708	35	35
1243	nonstudy	12months	35	35	35	35	35	35	35	35	35	35	35	35	35	35	35	35	35	35	35
1244	placebo	24months	35	29.561	29.702	35	35	35	35	35	19.169	35	28.401	35	35	35	35	35	35	35	35
1245	nonstudy	24months	35	32.896	28.978	35	35	35	35	34.752	19.974	35	21.469	35	26.215	35	20.267	35	25.769	35	35
1246	nonstudy	24months	34.839	35	27.469	35	35	35	35	35	35	35	34.869	35	23.001	35	35	32.047	25.49	30.008	35
1247	nonstudy	0months	25.417	35	27.992	35	35	35	35	30.893	35	35	24.888	35	21.049	35	35	35	21.437	35	35
1248	nonstudy	12months	35	35	35	35	35	35	35	22.887	35	35	27.494	35	35	35	35	35	35	35	35
1249	azithro	0months	28.007	35	31.456	35	35	35	35	35	35	35	15.488	35	16.837	35	35	35	19.229	35	30.089
1250	nonstudy	0months	35	35	28.937	35	35	17.247	35	34.733	35	35	33.361	35	35	35	35	35	27.554	35	35
1251	nonstudy	0months	35	35	29.487	35	35	35	35	27.143	35	35	17.082	23.859	17.019	35	35	35	20.74	35	35
1252	nonstudy	12months	35	35	35	35	35	35	35	35	35	35	35	35	32.988	35	35	35	30.796	35	35

1253	placebo	12months	35	35	27.605	35	35	35	35	18.281	35	35	35	35	25.259	35	35	35	26.345	35	35
1254	placebo	0months	35	32.148	35	35	35	35	33.635	35	35	35	27.764	35	20.648	35	35	35	26.932	35	35
1255	nonstudy	0months	35	35	35	35	35	29.768	35	35	35	29.898	34.473	35	35	35	35	35	35	35	35
1256	nonstudy	12months	35	35	35	35	35	35	35	35	35	35	24.606	35	23.334	35	35	35	27.29	35	35
1257	nonstudy	12months	35	35	25.864	35	35	35	35	24.907	35	35	21.022	35	24.04	35	35	35	22.769	35	35
1258	azithro	0months	33.105	35	27.928	35	35	35	35	27.308	35	35	24.857	35	29.896	35	35	35	23.95	35	35
1259	azithro	24months	35	35	31.65	35	35	35	35	35	35	35	32.145	35	23.967	35	35	35	30.112	35	35
1260	placebo	24months	33.7	35	31.588	35	35	35	35	28.897	35	35	35	35	21.668	35	35	33.6	35	31.012	35
1261	placebo	0months	30.401	35	35	35	35	35	27.735	35	35	35	20.236	35	20.915	35	33.777	35	25.052	35	35
1262	placebo	24months	35	35	35	35	35	35	35	33.366	35	35	20.097	35	35	35	35	35	25.577	35	35
1263	azithro	24months	35	35	27.004	35	35	35	35	35	35	28.103	23.226	35	23.219	35	22.828	33.905	27.951	35	35
1264	azithro	0months	35	35	35	35	35	35	35	35	33.9	35	25.359	35	25.574	35	35	35	23.702	35	35
1265	azithro	0months	35	35	31.175	35	35	35	33.75	35	35	35	29.776	35	22.596	35	35	35	25.785	35	35
1266	azithro	0months	35	35	35	35	35	35	35	35	19.364	35	21.309	35	24.362	35	35	35	23.503	35	35
1267	nonstudy	0months	35	35	35	35	35	35	35	33.785	35	35	26.674	35	35	35	35	35	21.61	35	35
1268	nonstudy	24months	35	35	31.828	35	35	35	35	35	27.305	35	24.415	35	19.889	35	34.345	35	27.712	35	35
1269	nonstudy	0months	31.34	35	34.332	32.972	35	35	35	34.17	35	35	22.635	35	24.337	35	35	35	25.594	35	35
1270	azithro	24months	35	35	35	35	35	35	35	35	17.164	35	32.26	35	35	35	35	26.515	35	35	35
1271	nonstudy	24months	35	30.698	20.159	35	35	35	35	35	35	35	23.017	35	21.124	35	35	35	24.39	35	35
1272	nonstudy	12months	27.127	33.587	22.842	35	35	35	35	35	35	35	17.023	35	35	35	35	35	19.291	35	33.137
1273	azithro	12months	35	35	31.43	35	35	35	35	35	35	35	16.658	35	20.506	35	35	35	20.884	35	35
1274	nonstudy	0months	35	35	35	35	35	33.338	35	35	35	28.513	35	35	35	35	35	35	35	35	35
1275	nonstudy	12months	35	35	35	35	35	35	35	25.881	35	35	35	35	25.96	35	33.533	28.894	22.921	35	35
1276	nonstudy	0months	29.233	35	35	35	20.742	35	35	35	35	35	35	35	21.913	35	35	35	22.836	35	35
1277	nonstudy	0months	35	35	30.741	35	35	35	35	35	30.153	35	21.77	35	20.606	35	35	35	27.835	35	35
1278	nonstudy	24months	35	30.145	26.026	35	35	35	35	35	35	35	26.854	35	27.956	35	35	35	26.111	35	35
1279	azithro	24months	35	35	25.754	35	35	35	35	35	19.214	35	27.637	35	32.015	35	35	35	32.439	35	35
1280	nonstudy	24months	35	35	35	35	35	35	27.07	35	35	35	20.454	35	20.786	35	21.979	35	23.614	35	35
1281	azithro	24months	35	35	33.241	35	35	35	35	35	35	35	21.655	35	22.942	35	29.755	35	26.555	35	35
1282	nonstudy	12months	35	35	28.703	35	35	35	35	30.029	35	35	22.776	35	21.836	35	35	35	28.63	35	35
1283	nonstudy	12months	35	35	35	35	35	35	20.215	35	35	35	21.569	35	20.538	35	35	35	23.933	35	35
1284	placebo	24months	35	35	35	35	35	35	35	35	35	35	35	35	21.567	35	35	31.899	23.913	35	35
1285	nonstudy	0months	35	35	35	35	35	35	35	35	35	35	21.092	35	21.285	35	35	35	23.044	35	35
1286	azithro	0months	35	35	33.865	35	35	35	24.539	35	35	35	16.949	35	19.48	35	35	35	22.471	35	35
1287	azithro	0months	35	35	29.494	35	35	35	35	26.199	35	35	18.662	35	20.57	35	32.709	35	29.126	35	35
1288	nonstudy	0months	35	35	35	35	35	35	35	35	35	35	21.258	35	22.047	35	23.758	35	24.671	35	35
1289	azithro	0months	35	35	28.067	NA	NA	NA	NA	NA	NA	35	21.473	35	20.933	35	35	35	25.05	32.905	35
1290	nonstudy	24months	35	35	23.787	35	35	35	35	28.123	35	35	21.741	35	23.909	35	35	35	24.728	35	35
1291	placebo	0months	35	35	26.4	35	35	35	35	35	35	35	22.782	35	21.483	35	35	35	26.409	35	35
1292	nonstudy	24months	35	35	28.619	35	35	35	35	35	19.24	35	19.234	35	26.06	35	35	35	27.018	35	35
1293	placebo	24months	35	35	23.535	35	35	35	35	35	35	35	19.295	35	20.247	35	35	35	24.575	35	35
1294	azithro	24months	35	35	21.776	35	35	35	32.37	25.976	35	35	26.933	35	22.402	35	25.521	35	23.421	35	35
1295	azithro	0months	35	35	22.229	35	35	35	35	35	35	35	22.824	35	21.812	35	35	35	27.118	35	35
1296	nonstudy	0months	35	35	35	35	35	35	35	35	35	35	24.305	35	21.88	35	32.068	35	26.263	32.328	35
1297	placebo	0months	35	35	35	35	35	35	35	31.386	35	35	35	35	23.059	35	21.851	35	26.804	35	35
1298	placebo	0months	35	34.097	35	35	16.964	35	35	35	35	35	23.491	35	33.415	35	35	35	29.948	35	35
1299	placebo	12months	35	34.132	35	35	35	35	35	29.793	35	35	23.448	35	21.825	35	35	35	27.383	35	35
1300	azithro	24months	24.693	35	35	35	35	35	35	35	35	35	19.435	35	35	35	35	35	35	35	35
1301	placebo	0months	35	35	32.048	35	35	35	35	35	35	33.163	21.381	35	20.177	35	35	35	29.019	35	35
1302	placebo	0months	23.103	35	27.888	35	35	35	35	35	35	35	19.636	35	21.651	35	35	35	26.808	35	35
1303	nonstudy	0months	34.273	35	26.869	35	35	35	35	35	35	34.169	26.638	30.817	20.2	35	34.817	35	24.943	35	35
1304	nonstudy	0months	35	35	35	35	35	35	35	35	35	35	18.815	19.195	20.599	35	35	35	26.344	35	35
1305	placebo	0months	30.507	35	25.977	35	35	35	29.207	35	35	35	15.527	35	21.187	35	35	35	24.836	35	35
1306	azithro	12months	32.365	35	29.544	35	35	35	35	24.955	35	35	21.79	35	22.983	35	28.803	35	24.55	35	35
1307	nonstudy	24months	35	35	35	35	35	35	35	35	35	35	35	35	19.833	35	35	35	26.852	35	35
1308	azithro	12months	35	35	26.487	35	35	35	35	35	35	35	26.979	35	21.644	35	35	35	32.898	35	35
1309	azithro	24months	35	35	32.376	27.5	35	35	35	29.551	35	35	19.774	35	23.067	35	25.821	35	24.232	35	35
1310	azithro	0months	31.827	35	26.928	35	35	35	35	26.956	35	35	20.011	35	20.139	35	35	35	23.363	35	35
1311	azithro	24months	32.355	35	35	35	35	35	35	35	35	35	22.521	35	27.958	35	22.966	35	23.223	35	35
1312	azithro	12months	33.427	35	35	35	35	35	35	35	35	35	23.009	35	35	35	35	35	27.977	35	35
1313	azithro	12months	35	35	35	35	35	35	17.804	35	35	35	35	35	19.763	35	35	33.382	24.275	35	35
1314	azithro	24months	35	35	35	35	35	35	34.247	35	16.149	35	20.072	35	30.56	35	35	35	24.555	35	35
1315	nonstudy	24months	35	35	26.184	35	35	35	35	26.91	35	35	33.967	35	35	35	35	35	22.615	35	35
1316	nonstudy	24months	23.275	35	35	35	16.091	35	35	35	35	35	25.223	35	19.773	35	27.621	35	25.946	35	35
1317	nonstudy	0months	35	35	35	35	35	35	35	31.148	35	35	29.836	35	21.911	35	35	35	29.098	35	35
1318	nonstudy	24months	31.009	33.956	24.524	35	35	35	35	23.866	35	34.915	20.356	35	21.091	35	35	35	25.294	35	34.192

1319	nonstudy	24months	35	35	35	35	35	35	35	35	35	35	35	35	21.011	35	35	34.444	26.488	35	35
1320	placebo	0months	35	35	35	35	35	35	35	35	35	35	33.472	35	21.785	35	35	35	22.44	35	35
1321	nonstudy	0months	34.135	35	35	35	35	35	35	35	35	35	16.722	35	17.649	35	35	35	21.997	35	35
1322	nonstudy	0months	35	28.657	23.729	35	35	35	35	26.196	34.377	35	18.657	35	21.718	35	30.921	31.14	22.213	35	34.185
1323	azithro	0months	35	35	31.166	35	31.259	35	35	35	35	35	18.1	35	22.205	35	33.492	35	21.163	35	35
1324	azithro	24months	35	35	35	35	35	35	35	35	34.561	35	21.41	35	21.907	35	35	35	24.313	35	35
1325	nonstudy	24months	35	35	35	35	35	35	35	35	16.058	35	35	35	28.478	35	35	35	24.74	35	35
1326	nonstudy	24months	35	35	32.485	35	35	21.559	35	35	35	35	22.64	35	25.265	35	35	35	24.605	35	35
1327	nonstudy	0months	35	35	32.642	35	35	35	35	26.077	35	35	22.983	35	32.772	35	35	35	24.453	35	35
1328	azithro	0months	23.424	35	28.271	35	35	35	20.228	35	35	35	22.603	35	29.084	35	35	35	18.993	35	35
1329	placebo	24months	35	35	19.27	35	35	35	35	35	35	35	24.818	35	27.911	35	35	35	34.326	35	35
1330	placebo	0months	35	35	27.767	35	35	35	35	35	35	35	21.638	35	19.201	35	35	35	22.129	35	35
1331	placebo	24months	35	35	31.139	35	35	35	24.238	35	35	35	20.078	35	18.885	35	35	35	21.585	35	35
1332	nonstudy	0months	33.227	35	29.285	35	35	35	28.632	35	35	35	19.183	35	23.051	35	35	35	21.59	35	35
1333	azithro	0months	35	35	22.451	35	35	35	35	31.967	35	35	25.763	35	27.46	35	35	35	24.334	35	35
1334	azithro	24months	35	35	35	35	35	35	34.548	35	35	35	35	35	35	35	35	35	35	35	35
1335	azithro	24months	32.495	35	25.684	35	35	35	34.472	28.149	35	35	23.44	35	21.786	35	35	35	21.376	35	35
1336	nonstudy	0months	35	35	35	35	35	35	35	35	35	35	19.459	19.221	21.506	35	35	35	26.409	35	35
1337	nonstudy	0months	35	35	32.677	35	35	35	35	35	35	34.011	19.422	35	21.796	35	35	35	29.279	35	35
1338	placebo	12months	35	35	29.885	35	35	35	35	35	35	35	22.262	35	23.193	35	30.725	35	24.751	35	35
1339	nonstudy	24months	35	35	35	35	20.151	35	35	35	35	35	35	35	19.913	35	35	35	27.287	35	35
1340	azithro	0months	35	35	31.039	35	35	35	35	35	35	35	35	35	26.892	35	35	35	35	35	35
1341	nonstudy	0months	35	35	26.803	35	35	35	35	35	35	35	21.696	35	18.734	35	35	35	21.991	35	35
1342	azithro	0months	35	35	35	35	35	31.958	35	35	35	31.874	35	35	33.316	35	35	35	35	35	35
1343	nonstudy	24months	35	35	35	35	35	35	35	27.817	35	35	35	35	35	35	35	27.806	35	35	35
1344	azithro	0months	35	35	27.528	35	35	35	35	35	35	35	26.893	35	20.378	35	35	35	27.617	35	35
1345	nonstudy	24months	35	35	23.396	35	35	35	35	35	35	35	27.371	35	24.716	35	35	35	27.292	35	35
1346	nonstudy	24months	35	35	35	35	35	24.8	35	35	35	35	30.183	35	21.553	35	35	35	22.739	35	35
1347	azithro	0months	34.974	35	31.723	35	23.304	35	35	35	35	35	15.47	35	29.641	35	35	35	25.399	35	35
1348	azithro	24months	35	35	35	35	35	35	23.867	35	35	35	24.742	35	35	35	35	35	28.361	35	35
1349	nonstudy	0months	35	35	35	29.994	24.627	35	35	35	35	35	18.49	35	21.792	35	35	35	25.895	35	35
1350	placebo	24months	21.114	35	28.514	35	35	35	35	35	35	35	26.354	35	32.577	35	35	35	35	35	35
1351	nonstudy	0months	35	35	26.638	31.972	35	35	35	35	35	35	22.776	35	20.554	35	35	35	23.723	35	35
1352	placebo	0months	32.672	35	28.373	35	35	35	35	35	35	35	16.365	35	20.006	35	35	35	21.993	35	35
1353	placebo	24months	29	35	35	35	35	35	20.468	35	35	35	19.297	35	20.539	35	35	35	24.698	35	35
1354	nonstudy	24months	32.829	35	26.981	35	35	35	35	35	20.681	35	25.216	35	24.567	35	21.445	35	24.121	35	35
1355	nonstudy	0months	35	35	26.296	35	35	35	35	35	35	35	24.54	35	21.71	35	35	35	23.85	35	35
1356	nonstudy	24months	35	35	35	35	35	35	35	35	35	35	34.936	35	23.749	35	35	34.86	22.889	35	35
1357	nonstudy	24months	35	35	30.266	35	35	35	32.671	35	35	35	20.793	35	25.274	35	35	35	22.67	35	35
1358	placebo	0months	35	35	35	35	35	35	35	35	35	35	27.188	35	25.273	35	35	35	26.496	35	35
1359	azithro	0months	35	35	35	35	35	35	35	35	35	35	35	35	24.657	35	35	32.773	20.463	35	35
1360	nonstudy	0months	35	35	35	35	35	35	35	35	35	35	35	35	35	35	35	35	20.553	35	35
1361	nonstudy	12months	35	35	30.943	35	35	35	35	35	35	35	28.857	35	35	35	35	35	26.985	35	35
1362	nonstudy	0months	35	34.832	31.894	35	35	35	35	35	35	35	19.168	35	26.998	35	35	35	35	35	35
1363	placebo	12months	35	35	35	35	35	35	35	35	35	35	35	35	35	35	35	35	29.969	35	35
1364	nonstudy	24months	35	35	27.044	35	35	35	35	28.478	35	35	22.859	35	22.075	35	32.139	35	23.625	35	35
1365	azithro	0months	31.288	32.671	33.553	35	35	33.619	35	35	35	35	25.085	35	22.888	35	35	34.504	28.378	35	35
1366	placebo	24months	24.842	35	28.05	35	35	35	35	35	35	35	29.442	35	17.802	35	35	35	22.703	35	35
1367	nonstudy	0months	35	35	30.646	35	35	35	31.588	35	34.562	35	25.82	35	23.925	35	35	35	35	35	35
1368	nonstudy	24months	35	31.353	35	35	35	35	35	27.01	35	31.117	22.628	35	22.352	35	35	35	22.429	35	35
1369	nonstudy	12months	35	35	24.952	35	35	35	35	22.284	35	35	25.048	35	25.917	35	35	35	22.72	35	35
1370	placebo	0months	35	35	33.926	35	35	32.683	19.046	35	35	35	18.75	35	26.843	35	35	35	22.614	35	35
1371	azithro	0months	35	35	23.321	35	35	35	35	35	35	35	20.113	35	28.757	35	19.076	35	21.711	35	35
1372	azithro	24months	35	35	28.929	35	35	35	35	35	35	35	35	35	29.304	35	35	35	27.467	35	35
1373	nonstudy	12months	35	35	31.04	35	35	35	35	35	35	35	35	35	19.875	35	35	35	17.979	35	35
1374	azithro	0months	34.241	35	24.804	35	35	35	35	35	35	35	23.189	35	20.21	35	35	35	24.38	32.851	35
1375	nonstudy	12months	35	35	32.323	35	34.769	35	35	35	35	35	19.932	35	24.793	35	35	35	25.398	35	35
1376	nonstudy	24months	35	35	35	35	35	35	35	35	35	35	35	35	35	35	35	29.645	35	35	35
1377	nonstudy	24months	35	35	35	35	35	35	35	35	35	32.547	22.46	35	26.464	35	29.969	35	22.849	35	35
1378	nonstudy	24months	35	35	34.236	35	35	35	35	35	19.738	35	20.838	35	24.17	35	35	35	27.613	35	35
1379	nonstudy	0months	35	35	29.361	35	35	35	35	32.16	35	35	35	33.455	35	35	35	35	27.957	35	35
1380	placebo	24months	35	35	24.999	35	35	35	35	25.04	35	35	24.314	35	22.245	35	35	35	33.594	35	35
1381	placebo	0months	35	35	35	35	35	35	35	32.633	35	35	35	35	19.145	35	31.041	35	25.133	35	35
1382	azithro	12months	35	35	35	35	35	35	31.34	27.779	35	35	22.728	35	23.129	35	35	35	26.575	35	35
1383	placebo	12months	35	35	35	35	35	35	35	28.961	35	35	35	35	28.55	35	35	35	35	35	35
1384	azithro	0months	35	35	35	35	35	35	35	35	35	35	19.168	35	21.526	35	35	35	33.545	35	35

1385	azithro	12months	21.834	35	26.306	35	35	35	35	25.225	35	35	21.037	35	20.702	35	33.387	35	24.878	35	35
1386	azithro	0months	35	35	35	35	35	29.84	35	35	35	29.387	35	35	35	35	35	35	35	35	35
1387	azithro	0months	35	35	30.773	35	24.144	35	35	35	35	35	21.215	35	22.648	35	35	35	24.7	35	35
1388	nonstudy	0months	35	35	35	35	35	35	35	30.667	35	35	19.767	35	19.929	35	21.091	35	22.809	35	35
1389	azithro	0months	30.052	35	33.094	35	18.415	35	35	35	35	35	21.852	35	26.865	35	35	35	28.834	35	35
1390	azithro	24months	26.102	35	31.594	35	35	35	35	27.051	35	35	22.165	35	22.473	35	35	35	25.444	35	35
1391	azithro	24months	35	35	35	35	35	35	35	35	35	35	35	35	35	35	35	35	35	35	35
1392	nonstudy	24months	35	31.766	35	35	35	35	35	25.943	35	35	22.988	35	22.158	35	35	35	28.599	35	35
1393	nonstudy	24months	35	35	34.005	35	35	35	35	35	35	35	33.338	35	35	35	35	35	20.188	35	35
1394	placebo	24months	35	35	26.311	35	35	35	35	35	35	20.324	24.171	35	20.44	35	35	29.531	28.152	35	35
1395	placebo	24months	35	35	35	35	35	35	35	35	35	35	35	35	25.331	35	35	35	33.464	35	35
1396	azithro	0months	35	35	30.474	35	35	35	35	35	35	35	31.989	35	25.143	35	35	35	26.862	35	35
1397	azithro	24months	35	35	26.403	35	35	35	34.372	35	35	35	17.053	35	20.376	35	35	35	35	35	35
1398	azithro	12months	35	35	35	35	35	35	35	26.058	35	35	21.283	35	19.334	35	35	35	26.351	35	35
1399	azithro	24months	35	35	32.32	35	35	35	35	35	35	35	20.507	35	18.947	35	35	35	21.237	35	35
1400	placebo	24months	35	35	24.348	35	35	35	35	35	35	35	20.695	35	19.441	35	35	35	27.387	35	35
1401	placebo	24months	35	35	34.173	35	35	35	35	35	35	32.236	21.88	35	21.82	35	35	35	27.16	35	35
1402	nonstudy	0months	35	35	28.639	35	35	35	35	35	35	35	20.654	35	20.019	35	35	35	25.441	35	35
1403	nonstudy	12months	35	35	35	35	35	35	35	35	35	35	35	35	32.097	35	35	35	31.091	35	35
1404	nonstudy	0months	35	35	35	35	35	35	35	32.894	35	35	35	35	23.126	35	32.987	35	23.882	35	35
1405	nonstudy	24months	21.747	35	32.293	35	35	35	35	35	35	35	19.364	35	19.957	35	35	32.078	23.045	35	35
1406	azithro	24months	35	35	32.459	35	35	35	35	26.047	35	35	30.115	35	23.451	35	35	32.628	26.492	35	35
1407	nonstudy	0months	35	35	35	35	22.096	35	35	35	35	35	24.816	35	19.875	35	33.822	35	21.792	35	35
1408	nonstudy	12months	35	35	35	21.305	35	35	35	31.136	35	35	27.635	35	19.032	35	35	35	26.829	35	35
1409	azithro	0months	35	35	35	35	35	35	35	35	35	33.858	18.499	18.222	20.776	35	35	35	25.975	35	35
1410	azithro	0months	35	35	30.749	35	18.944	35	35	35	35	35	20.054	35	25.438	35	35	35	26.332	35	35
1411	nonstudy	0months	35	35	25.442	35	35	35	35	22.863	35	34.357	35	35	19.24	35	35	35	22.931	31.492	35
1412	nonstudy	24months	31.982	35	32.02	35	35	35	28.588	35	35	33.899	20.903	35	17.718	35	35	35	26.258	35	35
1413	nonstudy	12months	35	35	26.25	35	35	35	35	35	35	35	25.187	35	19.305	35	35	35	26.661	35	35
1414	placebo	0months	35	30.261	35	35	35	35	35	31.129	35	35	19.295	35	27.656	35	35	35	23.521	35	35
1415	placebo	0months	35	35	35	35	35	35	35	26.901	35	35	29.572	35	23.65	35	35	35	20.368	32.715	35
1416	placebo	12months	35	35	31.281	35	35	35	35	28.924	35	35	32.692	35	22.889	35	35	34.464	30.405	35	35
1417	nonstudy	24months	35	35	27.986	35	35	31.973	35	35	35	35	24.449	35	21.21	35	33.514	35	23.744	35	35
1418	placebo	0months	30.77	35	25.756	35	35	35	35	28.349	35	35	26.401	35	25.827	35	35	34.371	27.373	35	35
1419	placebo	0months	35	35	24.437	35	35	35	35	35	35	35	16.136	35	19.254	35	20.721	35	20.118	35	35
1420	nonstudy	0months	35	35	27.533	35	35	35	35	35	35	35	23.393	35	19.967	35	21.615	35	24.152	35	35
1421	azithro	0months	35	35	32.85	35	35	35	35	31.245	35	35	26.342	35	25.146	35	35	35	25.993	35	35
1422	nonstudy	24months	35	35	32.326	35	35	35	35	27.168	35	35	16.787	35	18.72	35	35	35	22.138	35	35
1423	placebo	24months	35	35	35	35	35	35	35	35	35	22.971	24.264	35	22.494	35	35	35	27.987	35	35
1424	nonstudy	24months	35	35	35	35	35	35	19.196	35	35	35	20.614	35	21.925	35	35	35	20.775	35	35
1425	nonstudy	12months	35	35	35	35	35	35	35	35	35	32.192	35	35	35	35	35	35	28.707	35	35
1426	placebo	24months	35	35	27.571	35	35	35	35	25.891	35	35	20.459	35	22.465	35	35	35	28.446	35	35
1427	azithro	0months	35	35	35	35	35	35	26.996	35	35	35	19.497	35	22.289	35	35	35	21.02	35	35
1428	placebo	24months	35	35	27.309	35	35	35	35	35	35	35	22.507	35	24.009	35	35	35	25.67	35	35
1429	azithro	12months	35	35	30.472	35	35	35	19.507	35	35	35	22.998	35	23.371	35	35	35	26.545	35	35
1430	placebo	24months	35	35	30.846	35	35	35	35	35	35	35	35	35	22.504	35	35	35	24.617	35	35
1431	azithro	24months	35	35	35	35	35	35	35	31.883	35	35	23.622	35	23.809	35	35	35	26.124	35	35
1432	nonstudy	24months	30.821	34.026	33.309	35	35	35	35	35	35	35	19.137	35	18.805	35	35	35	19.346	35	35
1433	nonstudy	24months	35	35	35	35	35	35	21.888	34.202	35	35	30.749	35	35	35	35	35	26.588	35	35
1434	placebo	0months	35	35	29.982	35	35	35	35	35	35	35	20.399	35	35	35	35	35	21.14	35	35
1435	placebo	24months	35	35	29.392	35	35	35	34.369	35	35	35	22.661	35	22.863	35	24.872	35	21.815	35	35
1436	nonstudy	0months	35	35	29.483	35	35	35	35	33.556	35	35	23.408	35	22.249	35	35	35	22.936	35	35
1437	placebo	24months	35	35	35	35	35	35	31.031	23.955	35	35	21.055	35	35	35	35	35	23.943	35	35
1438	placebo	0months	35	35	35	35	35	35	35	35	35	35	19.149	35	21.207	35	34.807	35	26.009	35	35
1439	azithro	24months	33.317	21.725	21.551	35	35	35	35	24.601	35	35	21.373	35	28.217	35	35	35	22.328	35	35
1440	azithro	0months	35	32.736	35	35	35	35	35	35	35	35	27.32	35	25.779	35	35	35	26.07	35	35
1441	azithro	0months	35	33.12	30.788	35	35	35	30.613	31.971	35	35	21.619	35	26.315	35	34.625	35	24.988	35	35
1442	nonstudy	0months	35	35	31.189	35	35	35	35	35	35	34.92	23.703	35	21.126	35	35	35	23.957	35	35
1443	nonstudy	24months	35	35	25.692	35	35	35	35	28.862	35	35	20.998	35	21.974	35	35	35	24.576	35	35
1444	nonstudy	24months	32.149	35	35	35	35	35	30.228	33.892	35	35	21.096	35	27.478	35	35	35	26.799	35	35
1445	nonstudy	0months	35	35	35	35	22.263	35	35	35	35	35	21.432	35	25.153	35	35	35	26.577	35	35
1446	nonstudy	0months	35	35	23.074	35	35	35	35	20.768	35	35	25.573	35	18.903	35	35	27.839	26.309	35	35
1447	nonstudy	0months	13.109	35	25.156	35	35	35	35	35	35	35	30.669	35	19.433	35	35	35	24.91	35	35
1448	nonstudy	24months	35	35	30.357	35	35	35	35	32.555	35	35	24.093	35	32.422	35	35	35	28.335	35	35
1449	nonstudy	0months	35	35	35	35	35	35	35	27.948	35	35	19.88	35	27.828	35	35	35	24.671	35	35
1450	placebo	12months	35	35	35	35	35	35	35	27.033	35	35	19.968	35	25.149	35	35	35	26.116	35	35

1451	nonstudy	0months	35	35	35	35	35	35	35	35	35	35	35	35	24.612	35	35	35	32.564	35	35
1452	placebo	24months	35	35	33.085	35	35	35	35	29.869	35	35	30.042	35	23.649	35	35	33.525	23.505	35	35
1453	nonstudy	0months	35	35	35	35	35	24.208	34.979	33.996	33.219	35	24.467	24.519	21.55	35	35	35	24.638	35	35
1454	nonstudy	0months	35	35	35	35	35	32.462	35	35	35	30.963	35	35	35	35	35	35	35	35	35
1455	placebo	12months	35	35	35	35	35	35	35	35	35	35	35	35	27.292	35	35	28.144	35	35	35
1456	azithro	0months	30.498	35	35	24.278	35	35	35	35	20.587	35	20.169	35	22.264	35	35	35	24.265	29.486	35
1457	placebo	12months	35	35	35	35	35	35	35	35	35	35	21.996	35	23.152	35	35	35	29.522	35	35
1458	nonstudy	0months	35	35	30.833	35	35	22.188	35	35	35	35	22.285	35	21.727	35	27.52	35	25.495	35	35
1459	placebo	0months	35	35	35	35	35	35	35	35	35	35	18.558	18.75	20.266	35	35	35	25.74	35	35
1460	azithro	0months	35	35	35	35	35	35	29.196	35	35	35	18.222	35	21.457	35	26.256	35	23.084	35	35
1461	nonstudy	0months	33.798	35	23.345	35	35	35	35	35	35	35	21.685	35	18.605	35	35	35	26.487	35	35
1462	nonstudy	0months	21.262	35	28.299	35	35	35	35	27.471	35	35	23.513	35	23.331	35	35	35	25.08	35	35
1463	nonstudy	24months	35	35	31.976	35	35	35	35	28.243	35	35	21.785	35	31.077	35	35	35	25.252	35	35
1464	nonstudy	0months	35	35	32.297	35	35	35	31.721	30.915	35	35	20.009	35	21.391	35	35	35	24.087	35	35
1465	azithro	24months	17.506	35	27.801	35	35	35	35	30.056	35	35	18.225	35	25.34	35	35	35	24.599	35	35
1466	placebo	0months	18.895	35	35	35	35	35	35	35	35	35	21.411	23.751	21.14	35	35	35	21.566	35	35
1467	nonstudy	12months	35	35	33.502	35	35	35	35	35	35	35	28.386	35	24.351	35	35	33.428	28.248	35	35
1468	nonstudy	12months	35	35	35	35	35	35	35	35	35	35	21.544	35	18.715	35	35	35	21.729	35	35
